# The origin of sheep settlement in Western Mediterranean

**DOI:** 10.1038/s41598-020-67246-5

**Published:** 2020-06-23

**Authors:** Asmae Kandoussi, Ismaïl Boujenane, Clément Auger, Bruno Serranito, Agnès Germot, Mohammed Piro, Abderrahman Maftah, Bouabid Badaoui, Daniel Petit

**Affiliations:** 1 Department of Animal Production and Biotechnology, Institut Agronomique et Vétérinaire Hassan II, PO Box 6202 Rabat-Instituts, 10101 Rabat, Morocco; 2 Glycosylation et différenciation cellulaire, EA 7500, Laboratoire Peirene, Université de Limoges, 123 av. A. Thomas, 87060 Limoges, Cedex France; 3 Department of Medicine, Surgery and Reproduction, Institut Agronomique et Vétérinaire Hassan II, PO Box 6202 Rabat-Instituts, 10101 Rabat, Morocco; 4 Laboratory of Biodiversity, Ecology and Genome, Mohammed V University, 4 Avenue Ibn Battouta, B.P, 1014 RP Rabat, Morocco

**Keywords:** Ecology, Evolution

## Abstract

The arrival of Neolithic culture in North Africa, especially domestic animals has been essentially documented from archaeological records. As the data relative to sheep are scarce, we studied the genetic relationship between Moroccan sheep breeds and Mediterranean ones using the sequencing of 628 bp of the mitochondrial DNA control region in 193 Moroccan individuals, belonging to six breeds, and 652 sequences from other breeds in Europe and Middle East. Through Network analysis and an original phylogenetically derived method, the connection proportions of each Moroccan breed to foreign ones were estimated, highlighting the strong links between Moroccan and Iberian breeds. The first founders of Moroccan sheep population were issued at 79% from Iberia and 21% from a territory between Middle East and Africa. Their calculated expansion times were respectively 7,100 and 8,600 years B.P. This suggests that Neolithization was introduced by a double influence, from Iberia and from another route, maybe Oriental or Sub-Saharan. The consequence of the environmental changes encountered by founders from Iberia was tested using different neutrality tests. There are significant selection signatures at the level of Moroccan and European breeds settled in elevated altitudes, and an erosion of nucleotide diversity in Moroccan breeds living in arid areas.

## Introduction

The introduction of domesticated mammals in Northwest Africa was linked to a change in food production from hunting-gathering to farming^1^. These changes were the result of cultural exchanges, followed by population movements well documented in Western Mediterranean^2^. Recent researches conducted by several archaeological teams proposed, with a high accuracy, the different steps of the diffusion of Neolithic, since about 120 sites have been studied and dated in the range of 12,000 (Epipalaeolithic) to 4,000 years B.P. (late Neolithic)^3^. Indices of cereal and legume cultivation were attested at around 7,460 to 7,200 years B.P. and of sheep domestication at around 7,460 years B.P., at Kaf-That-el-Ghar in the Mediterranean side of the Tangier Peninsula^4^. It has been questioned whether the route of the migration was from south to north across the Gibraltar strait or the reverse^2,5^. Beside the slight delay relatively to south Spain Neolithic culture recorded at around 7,700 years B.P.^1^, the common pottery production in either sides of Gibraltar strait argue for the European source of this culture found in Northwest Africa^6,7^. The progressive development of agriculture was associated with a local warming deduced from the measurement of δ^18^Ο on shell margin of *Phorcus turbinatus* specimens collected and consumed by humans in Ifri Oudadane site^8^.

The origin of local sheep populations in Morocco is still obscure. The different scenarios reported in the literature^9^ are controversial, and many of them are not based on convincing evidence. The majority of these authors reported that except the Mountain population that has existed in the country for a very long time, the other local breeds were introduced into the country by Phoenicians, Romans, Arabs or brought from the sub-Saharan regions. The main breeds recognized by the public authorities with a well-defined standard are the Sardi, Timahdite, Boujaad, D’man, Beni-Guil, Blanche de Montagne and the Noire de Siroua, whereas the remaining breeds are composed by non-standardized populations. At the national level, characterization work on sheep breeds has been based on morphological characters^10^ and blood protein polymorphism^11^. Recently, a study based on SNPs markers highlighted the differentiation between most of Moroccan and Algerian breeds, with a special focus on inter-population exchanges^12^. However, the information about the link between Moroccan and European breeds is still lacking. The use of mitochondrial DNA (mtDNA) makes it possible to disentangle the origin, evolutionary history and phylogeny of many populations. So far, five maternal lineages, or haplogroups, have been identified in domestic sheep around the world: haplogroups A of Asian origin, B of European origin, C encountered in South Asia, and D and E, considered as rare, encountered in Eurasia^13–17^. According to Tapio *et al*.^16^ and Sanna *et al*.^18^, the haplogroup B expanded around 6,400 years ago and reached Western Europe before the haplogroup A. As a result, many breeds represent an admixture of two to three haplogroups.

The aim of this work was to assess the relationships between Moroccan and Mediterranean breeds by using the control region (CR) of the mtDNA in order to reveal the history of sheep settlement in western Mediterranean, including their periods of expansion. Moreover, it was interesting to compare the genetic variability of such characterized breeds to foreign ones, and to highlight the selective pressure that might have affected the Moroccan breeds relatively to Mediterranean ones. Among the different sources of selection, we focused on the climatic constraints, taking advantage of the highly contrasted areas of Morocco described in a previous work^19^.

## Results

### Database and conventional phylogenetic analysis

A 628 bp-long sequence of the control region of mtDNA were sequenced from 193 Moroccan individuals, belonging to 6 breeds (access numbers in GenBank: MN229085-MN229277). To enrich the database, the use of these sequences as seeds allowed retrieving from NCBI Blastn tool 652 foreign sequences, distributed in 9 Iberian, 7 Italian, and 10 Oriental breeds. There are two non-native breeds: the Lacaune of Italy and the German Merino. The number of individuals per breed, their origin and their access number are indicated in Suppl. S1. 1.

**Figure 1:**
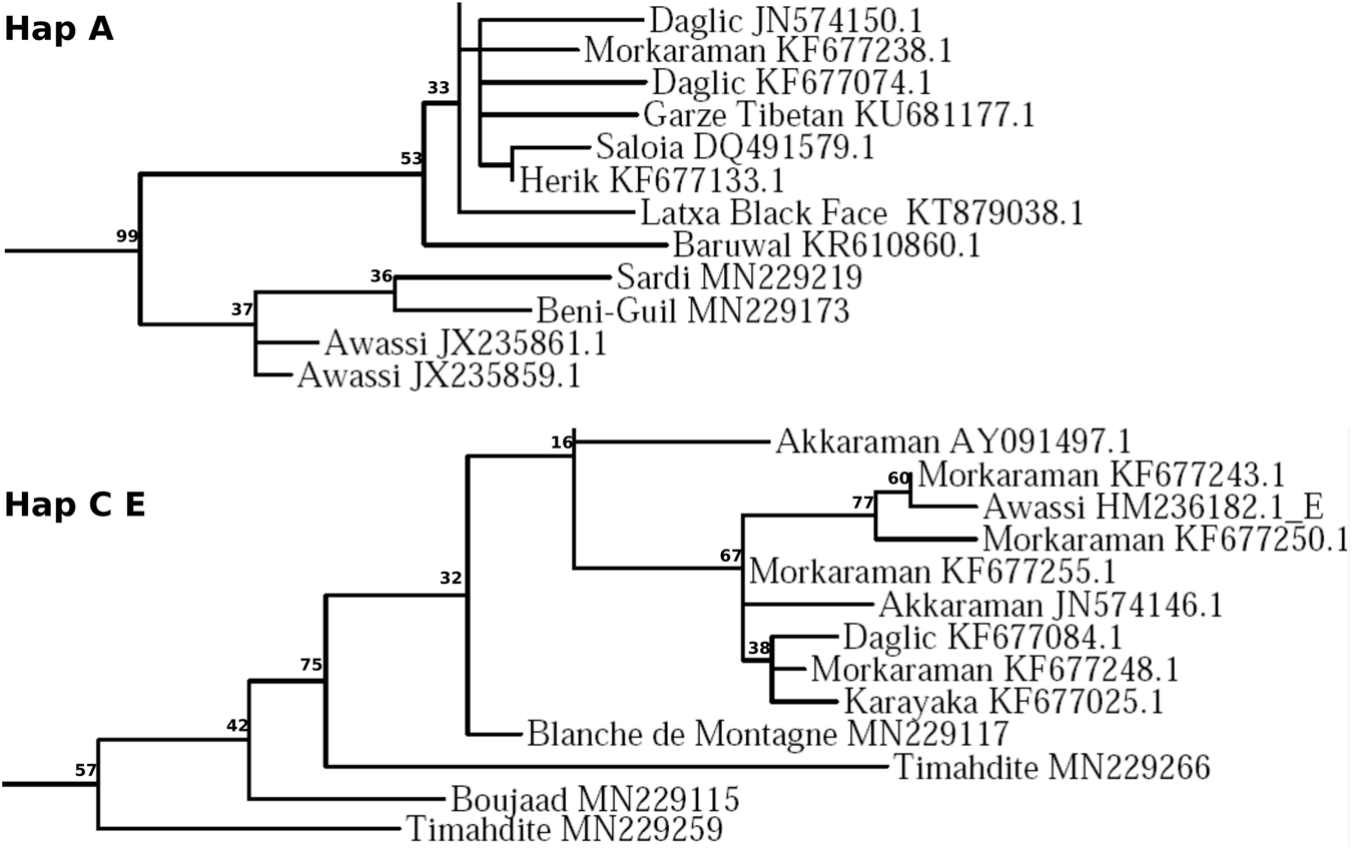
Position of the Moroccan breeds at the base of haplogroups A and C E. The bootstrap supports were calculated from 500 iterations.

The phylogenetic analysis generated a tree encompassing a total of 845 individuals, rooted by individuals of C and E haplogroups according to Meadows *et al*.^20^, and displayed in Suppl. S2. Among the 193 Moroccan individuals, 185 were found to belong to haplogroup B, i.e. 96% (Suppl S1. 2). It is worth noting that at the base of the C and E haplogroup clade, there is a series of four embedded Moroccan individuals (GenBank access numbers: MN229117, MN229266, MN229115, MN229259). Similarly, there are two sequences of Moroccan breeds also at the base of the A haplogroup clade (Fig. 1) (GenBank access numbers: MN229219, MN229173). Conversely, at the root of the B haplogroup clade, there are three Moroccan individuals associated to Portuguese Churra Badana breed, two others to Italian Merinizzata Italiana breed, and the last one to the Turkish Herik breed.

### Variation in the composition and connectivity among regions and breeds

**Figure 2:**
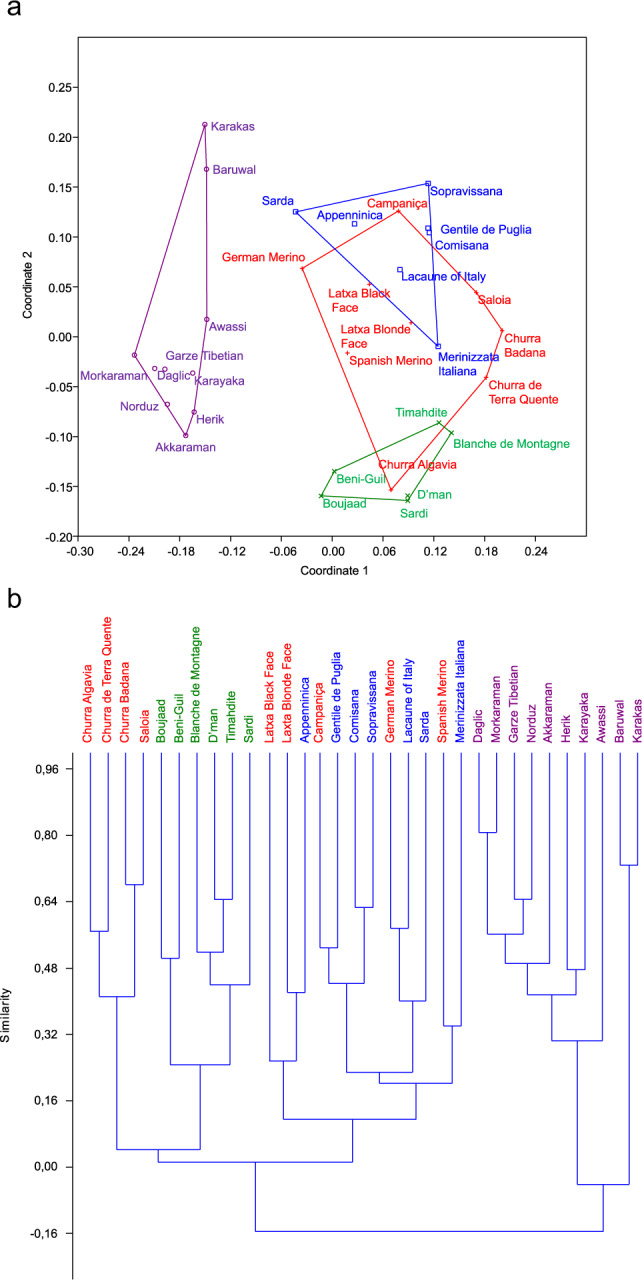
Relationship between breeds according to their geographic origin, from Newick-Extra program. (**a**) N-MDS analysis showing the intermediate position of Iberian breeds among western Mediterranean ones. (**b**) Cluster analysis highlighting the proximity between Moroccan and Portuguese breeds. Violet = Oriental breeds, Blue = Italian breeds, Red = Iberian breeds, Green = Moroccan breeds. The figure was drawn using PAST software v. 2.97, available at https://folk.uio.no/ohammer/past/.

The treatment of the topology information at the level 1, i.e. terminal branches, using Newick-Extra produced the table of affinities between breeds (Suppl. S1. 3), and then their proportions for each breed. As a first approach, the non-metric multidimensional scaling (N-MDS) analysis (Fig. 2a) revealed the isolated position of Oriental breeds, and the connections between Moroccan and Iberian breeds on one hand, and between Iberian and Italian breeds on the other hand. A more detailed view was given by the Cluster analysis (Fig. 2b), which highlighted three groups: (i) Oriental breeds, (ii) Moroccan breeds associated to four Portuguese breeds, and (iii) a mix of Iberian and Italian breeds.

In the same perspective of studying connectivity and kinship between populations, the genetic distances D_xy_ and D_a_ between breeds (Suppl. S1. 4) were visualized by NMDS. In both graphs (Suppl. S3. A-B), the Moroccan, Iberian and Italian breed ellipses were strongly superposed, while the Oriental breeds were scattered for D_xy_ genetic distance and relatively grouped for the D_a_ genetic distance. These results were confirmed by Cluster Analyses (Suppl. S3. C-D).

**Figure 3:**
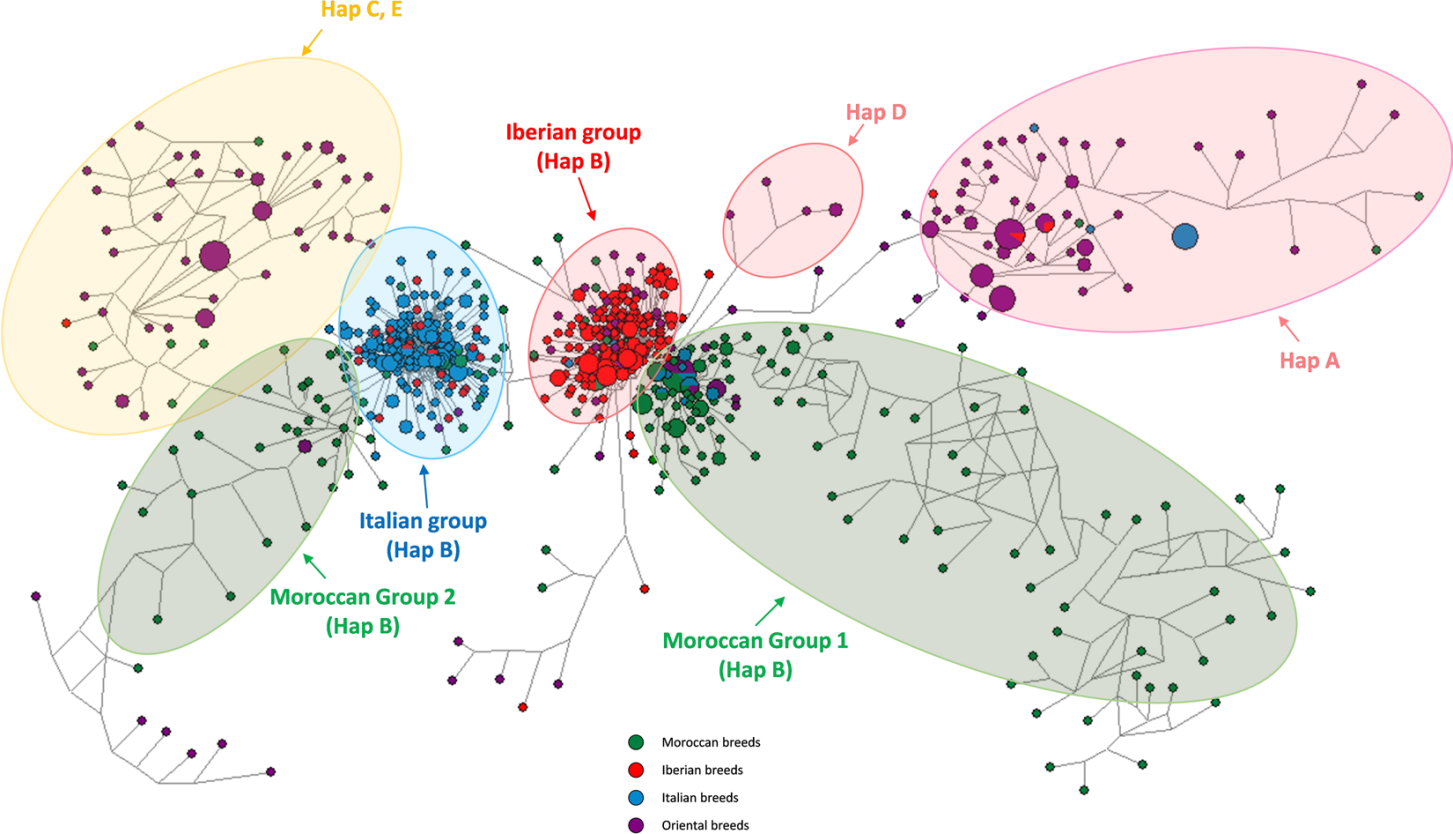
The individuals are organized in four haplogroup ensembles. The haplogroup B is dominant and comprises the Italian, the Iberian and the two Moroccan groups. The figure was drawn using Network software v.5.0.0.1, available on the website: http://www.fluxus-engineering.com/.

The use of Network software v.5.0.0.1 showed seven groups. On the left and the right sides, two groups gather individuals of haplogroups C-E and A respectively, dominated by animals of Oriental breeds (Fig. 3). The situation concerning haplogroup B is more complex as the individuals were organized in four groups: one Italian, one Iberian and two Moroccan ones, named 1 and 2, represented by 119 and 32 individuals respectively, making 62% and 17% of the actual Moroccan genetic background. The Iberian one was localized in the center and closer to the Moroccan group 1 than to the Italian one. The Moroccan group 2 was linked to the Italian one and the ensemble composed of individuals of haplogroups C and E. At a closer look, it appears that (i) the Italian and Iberian groups included 20 and 14 Moroccan individuals respectively, corresponding to c. 10% and 7% each of the total from Morocco; (ii) moreover, the Italian group also contained several Iberian (Spanish Merinos only) and Oriental individuals); (iii) the Iberian group itself hosted individuals of Oriental, Italian, and of the Moroccan group 1. As regard haplogroup D, composed of a few oriental individuals, its connections concern haplogroup A group and two ensembles belonging to haplogroup B, i.e. Iberian group and Moroccan group 1.

To test whether the genetic composition of the three western Mediterranean areas (Moroccan, Iberian, and Italian) was different, the AMOVA revealed significant molecular differences among breeds and within breeds for the 3 groups. Regardless of the compared groups, the main source of variation lay within populations, exceeding 96%. More interestingly, it appeared that there were significant differences between all the combinations of groups, except between Iberian and Italian ones. At a closer look, the difference between Moroccan and Iberian breeds was lower than between Moroccan and Italian ones (Suppl. S4. A).

In summary, the structuration of the western Mediterranean populations was relatively concordant with the results of procedures involving the Network, the AMOVA and Newick-Extra program, while the D_a_ and D_xy_ genetic distances provided a confusing view. To test specifically the level of compatibility between the Newick-Extra program and the Network methods, we generated 2 matrices that presented the percentages of connections between each Moroccan breed and the other breeds according to their geographical origin (Moroccan, Iberian, Italian or Oriental). In contrary to the Newick program for which this counting was straightforward, the count of connections from the network needed to be done manually. For each Moroccan breed, the numbers of individuals belonging to Italian, Iberian, Moroccan (1, 2 and 3) or Oriental (Haplogroups A, C, D, and E) were summed (Suppl. S1. 5). The correlation between the two matrices was highly significant (N = 24, r = 0.93, p < 0.001).

**Figure 4:**
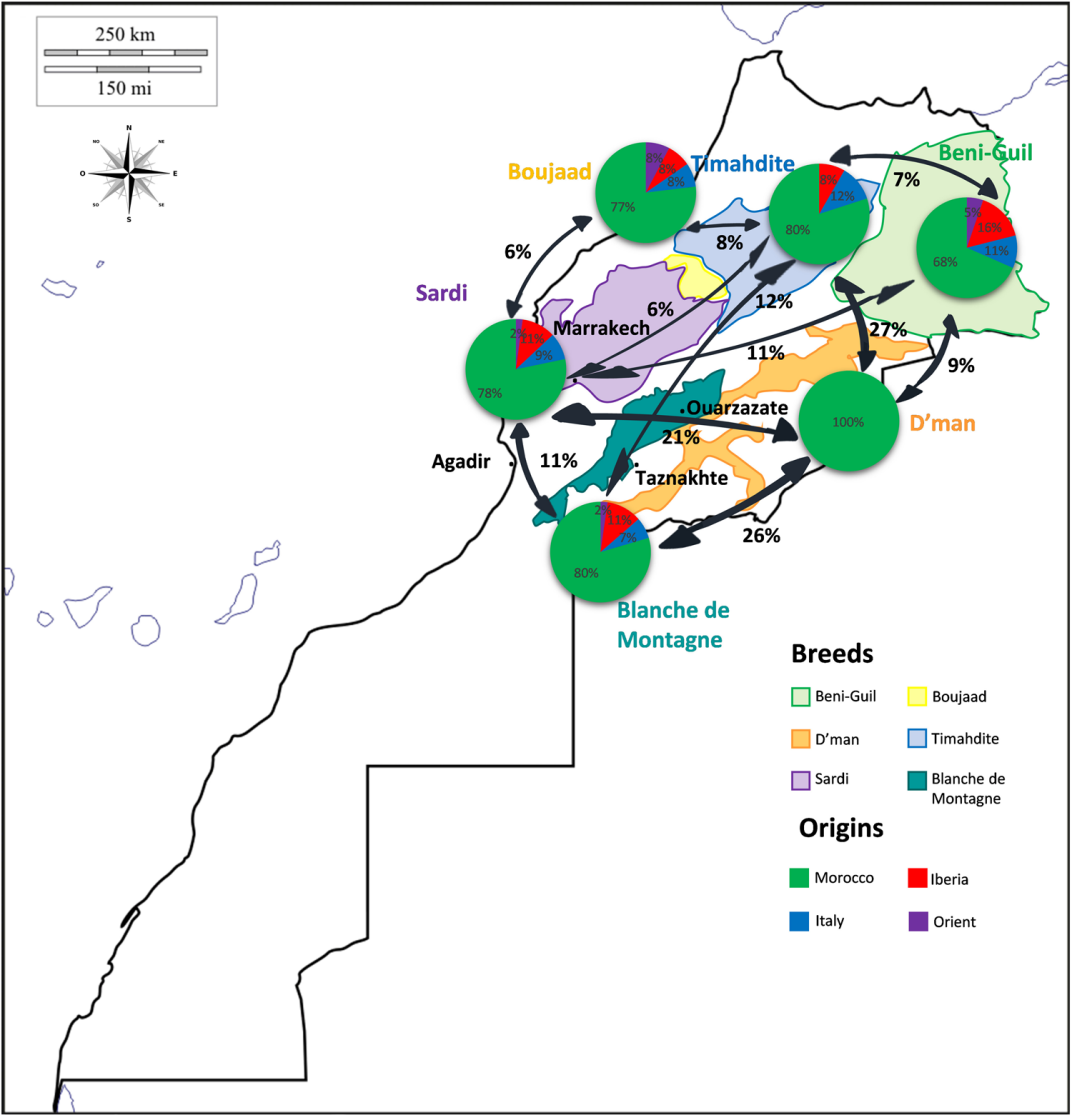
Map of connections between each Moroccan breed and (i) other Moroccan ones, and (ii) breeds of other Mediterranean areas.

For each Moroccan breed, the percentages of affinity (Fig. 4) showed contrasted situations toward Italian, Iberian and Oriental breeds. Beni-Guil breed had the highest proportion of influence from foreign countries (about a third), especially from Iberia, while D’man breed affinity was specifically Moroccan. The remaining Moroccan breeds had about a fifth relationship with foreign breeds, the Timahdite being the only breed devoid of connection with oriental breeds. There were moderate connections between Moroccan breeds themselves, except for the D’man, which showed more than 20% of relationship with the Timahdite, the Blanche de Montagne and the Sardi breeds. The analysis of the phylogenetic tree (Suppl. S2) indicated that all these connections were situated in the terminal branches of haplogroup B clade, and thus corresponded to recent exchange events.

### Climatic parameters

As it seems that the Italian, Iberian and Moroccan regions are centers of differentiation of sheep populations, it is useful to characterize the climatic constraints that exist in these three regions. The ANOVA conducted on the sum of localities recorded for each breed within each region lead to significant differences for most parameters (Suppl. S1. 6 and S3. E). As the altitudes recorded in the breeding areas in Morocco are higher than in Italy and Iberia, the winter temperatures expressed by m are colder. Given the south position of Morocco, the summer temperatures expressed by M are higher, leading to a decrease in rainfall (R) and vegetal productivity (Q3).

With regard to the breeds themselves, the mean values for climatic parameters were gathered in S1. 7.

### Genetic variability parameters

Among the groups defined from haplogroup B, it appeared that the Moroccan group 1 had a significant lower haplotype diversity H_d_ than the Iberian and Italian ones, but a higher nucleotide diversity π in relation to numerous singletons. At the level of breeds, it was expected that the variations in genetic diversity between Moroccan breeds followed the degree of affinity to foreign ones showed in Fig. 4. Effectively, the haplotype diversity H_d_ had its highest and lowest values in Beni-Guil and D’man, respectively (Suppl. S4. B., Suppl S1. 2 for all breeds).

### Neutrality tests and climatic constraints

**Figure 5:**
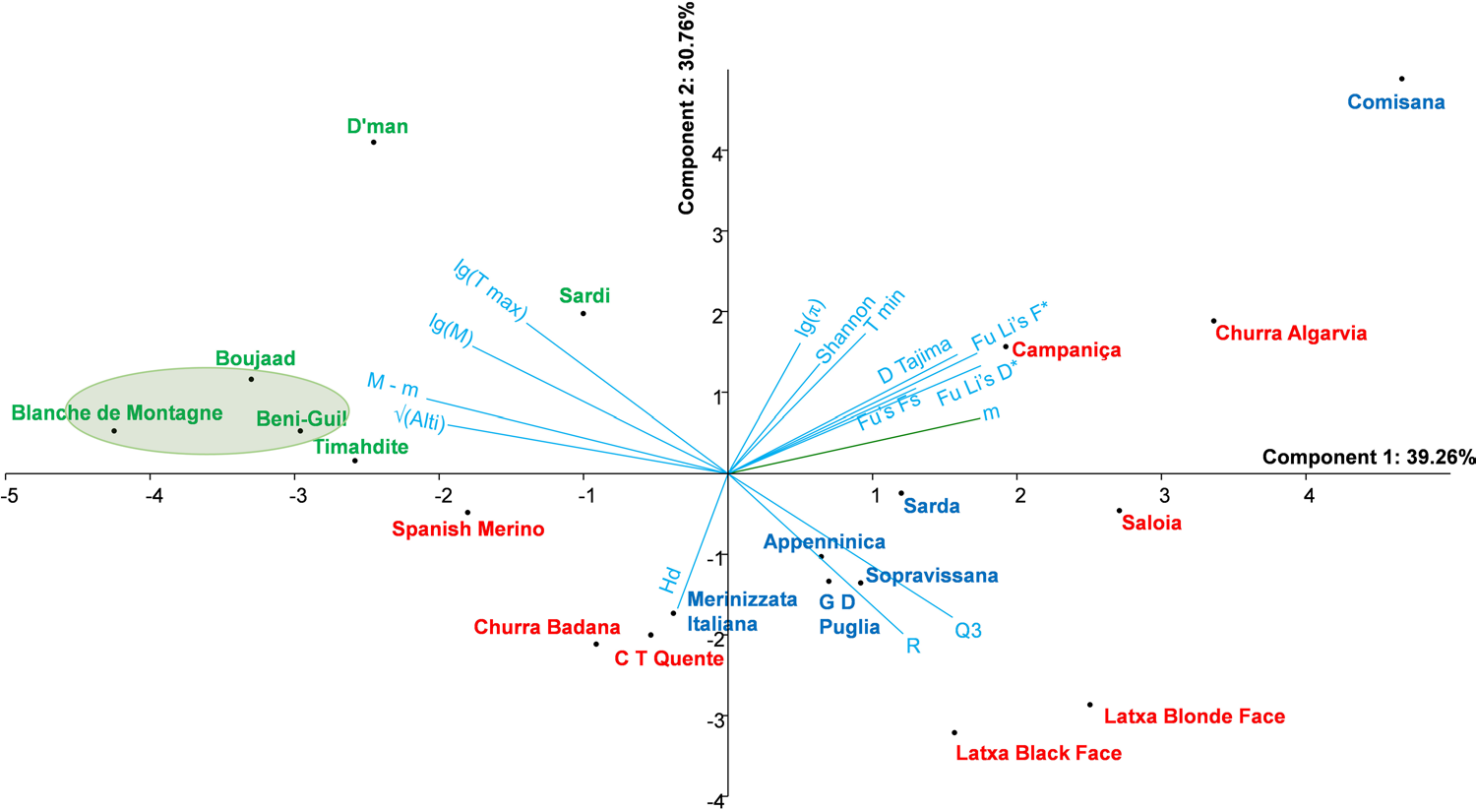
PCA of the climatic and genetic parameters. The ellipse gathers the breed significantly under selection (Fu and Li D* and F* parameters). Color code: see Fig. 2A. The figure was drawn using PAST software v. 2.97, available at https://folk.uio.no/ohammer/past/.

The calculated neutrality tests Fu and Li F* and D* among the four ensembles defined from haplogroup B, indicate lower significant values in Moroccan group 2 and Italian group than in the two other ones. These same tests applied to Moroccan breeds highlighted the Boujaad, the Blanche de Montagne and the Beni-Guil breeds, partially retrieved using Tajima D test. In contrast, Fu’s F_s_ revealed that all the Moroccan breeds were significantly out of neutrality. As it is difficult to quantify a direct influence of human selection by neutrality tests, it seems easier to investigate the effects of environmental constraints, especially the climatic parameters. For this purpose, a synthetic database was constructed by including both climatic and genetic data (Suppl. S1. 7). The relationships among all the climatic and genetic variables were well represented on the PCA first plan (70.02% of variance) (Fig. 5). The pluviometric coefficient Q_3_ and annual rainfall R varied in opposite to the temperature variables (M, T max, M – m) and altitude. In contrast, the variables m and T min seems to vary in the same direction as most genetic variables except H_d_. With regard to breeds, the Moroccan ones and the Iberian Spanish Merino and Churra Badana breeds were located towards high temperature and low rainfall variables. Another set of breeds was clearly associated to high rainfall, e.g. Sarda, Sopravissana, Saloia, Latxa Blonde and Black face. There is another set of three breeds (Campaniça, Churra Algarvia and Comisana) sharing high values of neutrality test variables. The result of correlation indicated that the variables T min and m were positively and significantly associated to π, Fu and Li F* and D*, and to some extent to D of Tajima (Suppl. S1. 7, Suppl. S3. F–H). If the breeds for which the significant neutrality tests Fu and Li F* and D* were considered (Blanche de Montagne, Boujaad and Beni-Guil), it appears that they were located in the PCA plan towards higher altitudes, low minimal temperatures (m and T min) and higher maximal temperatures (M and T max).

### Expansion time

To assess the prospective expansion events of western Mediterranean native breeds, the calculation of mismatch distribution revealed that the four groups of haplogroup B and breeds followed the coalescent simulation (p-value of SSD test >0.1, Suppl. S3. I,J), except the limit case of Comisana (p-value of SSD test = 0.08). The τ variations did not correlate to any climatic parameters (Suppl. S1. 7). From the first method of expansion dating, the yearly mutation rate could be estimated at 3.4 × 10^−5^ and 2.3 × 10^−5^ using 1.26 and 1.72 Myrs ancestors, respectively, resulting in expansion times at 140,000 and 207,000 years for the D’man breed for example. From the second method using archaeological records of Italy and Iberia, it appears that the Moroccan group 1 seems to have expanded later than Iberian one around 7,100 years B.P., whereas the Moroccan 2 group showed an earlier expansion than the Italian one itself around 8,600 years B.P. Due to the low population size of Moroccan individuals recorded in Italian and Iberian groups, no mismatch analysis could be done. With regard to the Moroccan breeds, the northern ones (Timahdite, Sardi, Beni-Guil and Boujaad) are in the range of c. 6,700–7,400 years B.P., while the southern ones are associated to 7,700 years B.P. for the Blanche de Montagne and 8,400 years B.P. for the D’man (Suppl. S1. 8).

## Discussion

The central issue of the study was to understand the extension of Neolithic culture associated with sheep domestication in western Mediterranean. The results obtained provide a new vision of the subject, and exclude the hypotheses emitted by historians who did not think to movements of sheep populations earlier than a few hundred years B.C.E.^9^

The different approaches conducted in the present study brought complementary information on the origin of Moroccan sheep breeds. If the Cluster Analysis generated by phylogeny and Newick-Extra method were considered, all the Iberian, Italian and Moroccan breeds are clearly separated from Oriental ones. This suggests that the western Mediterranean populations have undergone a long period of evolution during which numerous mutations have been accumulated. Moreover, it appears that the Moroccan sheep breeds are closer to Portuguese than to Spanish ones and even more so the Italian ones. This view is supported by the network results, where Iberian group is situated between the Italian and Moroccan groups. However, there are two Moroccan groups, a majority group 1 representing around 62% of the genetic background and linked to Iberian populations, and a minority group 2 around 17% having relationships with Italian and Oriental breeds. The individuals situated at the base of A and CE haplogroups, related to Oriental breeds, could be assign to the Moroccan group 2 in reason of their link and ancient origin, making this group up to c. 20% of the total genetic background. The question raised is to determine the significance of these 2 groups in the light of archaeological and expansion time evidences. For this purpose, the method based on mutation rate of control region lead to unrealistic dates, an issue previously discussed by Guo *et al*.^14^ who found a mutation rate of the same order of ours. It can be hypothesized a low accumulation of mutations in the genus *Ovis* (about a tenth) or an over-estimation for the dates of the two calibration ancestors. Using the second method based on the dating of early sheep remains in Italy and Iberia, the expansion date of Moroccan group 1 for example at 7,100 years B.P. is close to archaeological records at c. 7,400. As a result, the data provided by this second method were retained.

There is a progressive consensus among archaeologists for an Iberian origin of most features of Neolithic culture in Northwest Africa^21^, although Sánchez *et al*.^22^ argued for a reverse way for some aspects. This view was globally supported by the molecular dating and network approach of the present study, since the Moroccan group 1 expanded later than Iberian populations around 7,100 years B.P. This estimation is close to the indices of domestic sheep remains mentioned at around 7,460 years B.P., at Kaf-That-el-Ghar near Tangier Peninsula^4^. Concerning human populations, a work dealing with Ifri n’Amr or Moussa (at around 7,000 years B.P., early Neolithic) and Kelif el Boroud (at around 5,000 years B.P., upper Neolithic) near the Atlantic coast, showed that the early Neolithic populations were isolated, in the continuity of the upper Paleolithic ones in relation to Berber settlement, while the upper Neolithic individuals were strongly influenced by Iberian populations^21^.

About the origin of Moroccan group 2, the possibility of an ancient settlement of population from a route different from the Gibraltar strait was explored as for goat populations^23^. The absence of any relationship with Iberian breeds of haplogroup B strongly suggests that these individuals came to Morocco from Middle East via Africa, maybe from a south Italian – Tunisia route, or from an entirely African route. The connection between Italian and Moroccan individuals of group 2 support the 1^st^ idea. In contrast, the existence of several Moroccan individuals at the root of A and C E haplogroup clades argue for the second solution. Moreover, the dating of Moroccan group 2 expansion around 8,600 years B.P., more ancient than for Italian group, seems to favor this second solution. Unfortunately, the archaeological works are useless until now as the earliest sheep remains out of Morocco are dated around 7,000–7,500 years B.P. in Sinai Peninsula^24^. If our calculations were not overestimated due to calibration bias, we think that new investigations should be conducted in the south of Morocco, and of course in other areas of Africa, in order to detect very early Neolithic sheep remains. This idea does not appear to be unrealistic, since there is a lack of archaeological sites for the period around 8,000 years B.P., especially in the south of Atlas region^3^.

It is interesting to draw a parallel with the genetics of ancient humans. Van de Loosdrecht *et al*.^25^ established that between 15,000 and 14,000 years B.P. (upper Paleolithic), the Taforalt’s population, in the Mediterranean coast of Morocco, presented affinities with the population of Levant and to sub-Saharan Africans. However, the studies on the genetics of Sahelian sheep are still scarce^26–28^.

Besides the early founders of the two Moroccan groups, there were 20 individuals (around 10%) and 14 (around 7%) showing very similar sequences to Italian and Iberian groups, respectively, suggesting late introduction events southward. Conversely, the Moroccan group 1 contained 9 Italian individuals that are interpreted as a late event of importation from Morocco to Italy, maybe at the Roman’s epoch: these individuals had stayed a long time in the Moroccan territory, acquiring their genetic identity. Similarly, the Italian group included all the individuals of Spanish Merinos, a view supported by the text of 1^st^ century Roman Columella in chapter 7^29^, who indicated that the selection of fine wool sheep was conducted using individuals from Tarento region in South East Italy. Moreover, the 13 Oriental individuals found in the Moroccan group 1 could be due to recent trade events toward Turkey and beyond. Following the successive population movements of the founders on Moroccan territory, the animals have progressively differentiated into local populations and then breeds. As shown previously, the four breeds of the North, between 6,700 and 7,400 years B.P. have expanded later than the two breeds settled on south of Atlas, between 7,700 years B.P. (Blanche de Montagne) and 8,400 years B.P. (D’man).This dating suggests that some D’man ancestors would have reach Morocco via another route than Gibraltar strait, maybe from Africa. However, the origin of this last breed remains still obscure and new data on Sahelian breed could enlighten the situation. The four northern ones from Sardi in the West to Beni-Guil in the East were well genetically differentiated with less than 9% of connection. In contrast, the two southern ones, Blanche de Montagne and D’man had strong and recent links with Sardi and Timahdite breeds. The strong proportion of connections between the D’man and the other breeds were also found from the study based on SNPs over Northwest African sheep breeds^12^. It can be assumed that the D’man and the Blanche de Montagne, being small sized breeds^10^, were crossed with the biggest Sardi and Timahdite individuals to increase their meat production. Otherwise, the D’man being well known for its high prolificacy^30^, was crossed to Sardi, Timahdite and Blanche de Montagne breeds to improve their reproductive characteristics.

The European genetic component of Moroccan breeds, mostly Iberian, is actually highly dominant compared to the native “Berber” one. Given the climatic contrast between the two areas from each side of Gibraltar strait, it was expected that the drought and the high temperatures observed in Morocco would induce a selection pressure on the sheep flocks. Effectively, the shortage of rainfall and low vegetal productivity expressed by Q_3_ decreased the nucleotide diversity at the level of Moroccan breeds. At the level of European and Moroccan breeds, this correlation is not true, but the low temperatures (T min and m) induced a selection signature (Fu and Li F* and D* statistics) for the breeds settled in high lands above 650 m A.S.L (Blanche de Montagne, Boujaad, Beni-Guil, and Timahdite to some extent). The influence of these altitudes on low temperatures had also suggested a decrease of nucleotide diversity.

## Conclusion

Much effort has been made in the recent years to document the transition between the Paleolithic and Neolithic cultures in Morocco. The results presented here evidenced two successive waves of domestic sheep arrival on the Moroccan territory, corresponding to this transition: the most ancient one could be associated to the first Berbers around 8,600 years B.P., still representing 20% for the Moroccan genetic background, maybe as a response to the aridification of Northern Sahara at the end of the “African humid period”. The second one was the most important with 62% of this same background, and reached Morocco around 7100 years B.P. from Iberia. Two further independent importations of individuals from Iberia and Italy were recorded, less or equal to 10% of the genetic diversity each, but hardly datable due to their weak numbers. Archaeologists have shown an increase in exchanges with Iberia since 7,500 years B.P., in good agreement with the dating conducted on the expansion of most Moroccan breeds. However, for the special case of the D’man breed, the calculated expansion exceeded the period of human movements between Morocco and European continent, suggesting non-European route for the introduction of this breed ancestors. The human genetic researches argued for a continuity of local populations from broadly 15,000 to 7,000 years B.P., more or less related to Middle East, South and East African ones, and a sudden change of population since 5,000 years B.P., corresponding to a transition from hunting-gathering to agricultural practices.

## Material and methods

### Sample collection, DNA isolation and sequencing

Blood sampling was performed by veterinarians of the ANOC (Association Nationale Ovine et Caprine) during routine medical care or follow up, such that no ethical authorization was required. Breeders and ANOC consented to the blood samplings and their utilization for scientific purpose.

A total of 193 blood samples were taken from sheep belonging to six Moroccan sheep breeds: Sardi (n = 33), Timahdite (n = 37), Boujaad (n = 31), Beni-Guil (n = 31), D’man (n = 29), and Blanche de Montagne (n = 32). The blood samples were taken by an authorized person. The individuals were collected from farm breeders in the breeding areas of different breeds and supervised by the “Association Nationale Ovine et Caprine” ANOC, in order to ensure that they correspond to native breeds without introgression with foreign commercial or other local breeds.

Specimens were chosen according to their genealogy so that they are unrelated. Blood samples were taken from the jugular vein and collected in ethylene diamine tetra acetic (EDTA) acid tubes and frozen at −20 °C until extraction. Total genomic DNA was extracted from blood samples using an alkaline lysis method^31^. Details about PCR amplification and sequencing are reported in Suppl. S4. C. The sheep breeds sequences were recorded in GenBank (accession numbers in Suppl. S1. 1).

### Mitochondrial sequence analysis

#### Construction of database

**Figure 6:**
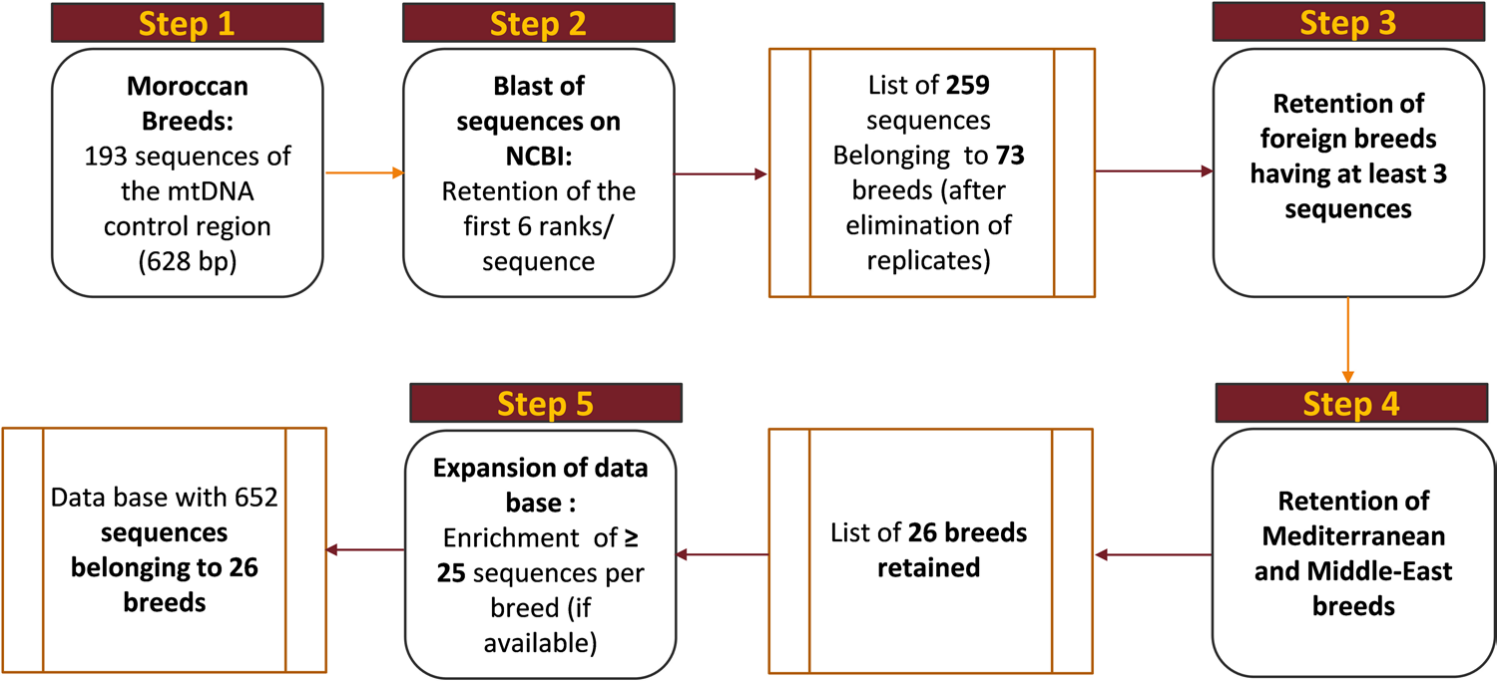
Workflow for data base construction.

We built a database of control region (CR) sequences including 193 from Moroccan and 652 from non-Moroccan breeds. This database was constructed by exploring the NCBI gene bank using BLASTn algorithm. Taking each Moroccan sequence as seed, we consistently selected at least the top six ranks per blasted sequence. Then, we looked at the following ranks to possibly retain those who had the same score as the sixth. Among all the breeds obtained, we retained those that showed more than three individuals, belonging to Mediterranean, African, and Near East regions. Then, we expanded the database by including 25 sequences or more per breed if available. The sequences selected were then recorded with their access number and the reference to their breed (Fig. 6). Furthermore, the data matrix included 5 mtDNA control region sequences from sheep representing the five mitochondrial haplogroups (A, B, C, D, E), in order to correctly assign each sequence to its proper haplogroup.

#### Analytical methodologies

The sequences were corrected and aligned using MEGA software v.10.0^32^, resulting in 628 pb available for further treatments.

In order to determine the genetic variation within populations, several diversity parameters were calculated with the DNAsp v5 program^33^: the number of haplotypes (H), the haplotype diversity (H_d_), the nucleotide diversity (π)^34^, the number of segregation sites (S), the number of singletons (S_g_) and the number of parsimoniously informative sites (P). In addition, four neutrality tests were performed: Fu and Li F*, Fu and Li D*^35^, Fu Fs^36^ and D of Tajima^37^ using DNAsp for each Moroccan, Iberian and Italian breed. The number of segregation sites (S), the singletons (S_g_) as well as the parsimoniously informative sites (P) were calculated for each breed by the “Polymorphic sites” option, while the remaining diversity indices were calculated by the “DNA Polymorphism” option. To calculate these previous parameters for each of the four groups within haplogroup B, the sequence numbers of Iberian and Italian ones were adjusted by a random method to the number recorded in Moroccan group 1, i.e. 119, to avoid a sampling size effect. The haplogroup frequencies were deduced from the phylogenetic analysis, and the haplogroup diversity from Shannon index calculated using PAST software v.2.97^38^.

To estimate the divergence between populations (breeds), the genetic distances D_a_ (the corrected average pair-wise differences) and D_xy_ (the average number of pair-wise differences)^34^ were calculated by DNAsp v5^33^. The obtained results were highlighted with the help of “Non-Metric Multidimensional Scaling Plot” and Cluster Analysis with User distance using PAST software v.2.97^38^. Connectivities as well as evolutionary relationships between different individuals were also analyzed through the construction of a phylogenetic tree by the Neighbor-Joining method, using the NETWORK software v.5.0.0.1 (available on the website: http://www.fluxus-engineering.com/). The Median-Joining Network^39^ is an haplotype network of all individuals. To reduce the large data set, the “Star Contraction” option was used. In order to simplify the graphical representation of the constructed network, the optional “MP calculation” procedure according to Neighbor-Joining method was used to remove unnecessary vectors and median links and to avoid reticulations. In addition, the length of the branches of some individuals were also modified manually, when necessary, to improve the visualization of results.

A phylogenetic analysis was carried out in order to reveal the affinities between Moroccan breeds and themselves and also to other breeds in the world. The phylogenetic tree was constructed using the Maximum Likelihood (ML) method implemented in MEGA software v.10.0^32^. The best model according to the lowest Bayesian Information Criterion corresponded to Hasegawa-Kishino-Yano model^40^, including Gamma distribution (5 discrete categories) and Invariant sites (G + I). The gap treatment was done by eliminating segments that had less than 95% presence (Partial Deletion). The robustness of branches was tested using 500 bootstrap replicates. The tree was redrawn using Seaview v.4^41^.

As expected, the phylogenetic tree produced several sets of individuals corresponding to each haplogroup, preventing to evidence the relationships between breeds. The use of the conventional genetic distance indices produced the same effects. To overcome this problem, we developed a new phylogeny-derived method called Newick-Extra (Suppl. S4. D).

Relationships between the studied breeds (asymmetric matrix generated by Newick-Extra) were visualized through the Non-Metric Dimensional Scaling Plot (NMDS) and Cluster analysis using Spearman’s Rho as distance metrics, using PAST software v.2.97^38^.

The Analysis of MOlecular VAriance (AMOVA) was computed to test significant differences between geographical breed groups, using ARLEQUIN v.3.5^42^. Sources of variation were considered in a single model: among groups, among breeds – within groups – and within breeds. Mismatch distribution was used for each European and Moroccan breed in order to address eventual demographic expansion events, by computing the τ parameter. This last one has shown to be useful to date historical expansion of human^43,44^, wild^45^, and domestic animals, e.g. goats^46^. To test the validity of an expansion hypothesis, the observed mismatch distribution was compared to the simulation values generated by coalescent algorithm using the probability associated to the sum of square deviation (SSD)^44^. In order to calculate the expansion time of breeds, two strategies were conducted. The first one used the formula τ = 2μt, where μ is the mutation rate for the whole considered segment and t is time in generations^47^. To calculate the mutation rate (μ) of the 628 pb segment, a data set was built, including two individuals of *Ovis aries*, *O. ammon*, *O. vignei*, per haplogroup and one for *O. orientalis*, *O. dalli* and *O. nivicola*. *nivicola* (access numbers in Suppl. S1. 1). After manual alignment, the topology given by ML with the same options as previously was saved as a user tree to run DNApars implemented in PHYLIP software v.3.695^48^, giving the possibility to get an exact count of mutations in the different branches. The ancestor of *O. vignei*, *O. orientalis*, and *O. aries* was calibrated at 1.26 Myrs, and the ancestor of this one with *O. ammon* at 1.72 Myrs^49^. The second method was based on a calibration using τ values for Iberian and Italian groups, defined in the network analysis, and archaeological records. According to Rowley-Conwy *et al*.^50^ and Sánchez *et al*.^22^, the earliest domestic sheep remains were found in South-East Italy around 8000 years B.P. and near Malaga in South East Spain around 7500 years B.P., respectively. The regression equation deduced was used to calculate the dates corresponding to τ values with 95% credible intervals for each group and breed.

### Climatologic data

The aim was to define the optimal climatic conditions for the breeds in order to detect eventual sources of natural selection on genetic variations. The methods developed by Petit and Boujenane^19^ was extended to the Moroccan Blanche de Montagne, Iberian and Italian studied breeds. The Oriental animals were not considered because they were distantly related to Moroccan breeds. Briefly, the geographic perimeter of the breeding area of a given breed was obtained through bibliographic data: anoc.ma, agraria.org, sprega.com and confelac.org for Moroccan, Italian, Portuguese and Spanish breeds, respectively. Concerning the Spanish Merino breed, information was taken from Lancioni *et al*.^51^. The following climatic parameters of at least 10 localities distributed in the breeding area were recorded from climate-data.org: Alti: altitude (m), m: minima mean of the coldest month (°C), M: maxima mean of the hottest month (°C), R: annual rainfall (mm), T max: annual maxima mean (°C), T min: annual minima mean (°C) for the period 1984 to 2014, and Q_3_ = $\frac{3.43{R}}{M-m}$ or pluviometric coefficient^52^. For each breed, the means of these parameters recorded for the different localities were calculated.

To test whether the regions showed significant differences in the climatic parameters, a one-way ANOVA conducted using SYSTAT vers. 12^53^ was considered with three modalities (Iberia, Italy and Morocco). The non-autochthon breeds (German Merino and Lacaune) were removed from the lists of Iberian and Italian breeds, respectively.

The relationships between genetic and climatic data were studied using principal component analysis and correlation analysis implemented in PAST software v.2.97^38^. Prior to the calculations, non-normal distributions were transformed (square root for Alti, Log for T max, M, and π). However, D of Tajima and the Shannon index were non-transformable.

## Supplementary information

Supplementary information.

## Data Availability

Newly-reported sequences in GenBank: MN229085-MN229277. The program written in R Neuwick-extra is available from BS on request. All data generated or analyzed during this study are included in this published article (and its Supplementary Information files).
