# Supplementary material for: The origin of sheep settlement in Western Mediterranean
Source: Sci Rep. 2020 Jun 23;10:10225. doi: 10.1038/s41598-020-67246-5 (PMC7311441; doi:10.1038/s41598-020-67246-5)
Supplement: Supplementary file 1 — Supplementary information. [file 41598_2020_67246_MOESM1_ESM.pdf]

SUPPLEMENTARY INFORMATION

The origin of sheep settlement in Western Mediterranean

Asmae Kandoussi, Ismaïl Boujenane, Clément Auger, Bruno Serranito, Agnès Germot, Mohammed Piro, Abderrahman Maftah, Bouabid Badaoui, and Daniel Petit

Supplementary Data S1: Supplementary tables

1. Access Numbers of sequences

Access numbers for phylogenetic analysis					
Breeds (Origin)	Population size	Access numbers	Breeds (Origin)	Population size	Access numbers
Akkaraman (Turkey)	18	AY091497.1	Lacaune of Italy (Italy)	30	KF228745.1
		JN574132.1			KF228746.1
		JN574133.1			KF228763.1
		JN574134.1			KF228766.1
		JN574135.1			KF228769.1
		JN574136.1			KF228770.1
		JN574137.1			KF228771.1
		JN574138.1			KF228772.1
		JN574139.1			KF228773.1
		JN574140.1			KF228774.1
		JN574141.1			KF228775.1
		JN574142.1			KF228776.1
		JN574143.1			KF228777.1
		JN574144.1			KF228779.1
		JN574145.1			KF228785.1
		JN574146.1			KF228786.1
		JN574147.1			KF228787.1
		KF677049.1			KF228788.1
Churra Algarvia (Portugal)	30	DQ491580.1			KF228789.1
		DQ491589.1			KF228790.1
		DQ491591.1			KF228791.1
		DQ491646.1			KF228792.1
		DQ491649.1			KF228793.1
		DQ491652.1			KF228794.1
		DQ491654.1			KF228795.1
		DQ491656.1			KF228796.1
		DQ491658.1			KF228797.1
		DQ491664.1			KF302447.1
		DQ491678.1			KF302453.1
		DQ491687.1			KF302460.1
		DQ491699.1			KF228640.1
		DQ491706.1			KF228641.1
		DQ491708.1			KF228642.1
		DQ491712.1			KF228643.1
		DQ491713.1			KF228644.1
		DQ491714.1			KF228645.1
		DQ491719.1			KF228646.1
		DQ491720.1			KF228647.1
		DQ491722.1			KF228648.1
		DQ491723.1			KF228649.1
		DQ491724.1			KF228650.1
		DQ491725.1			KF228651.1

		DQ491728.1			KF228652.1
		DQ491730.1			KF228653.1
		DQ491732.1	Merinizzata Italiana (Italy)	30	KF228654.1
		DQ491733.1			KF228655.1
		DQ491735.1			KF228656.1
		DQ491736.1			KF228657.1
		KF228617.1			KF228658.1
		KF228618.1			KF228659.1
		KF228612.1			KF228660.1
		KF228613.1			KF228661.1
		KF228614.1			KF228662.1
		KF228615.1			KF228663.1
		KF228616.1			KF228664.1
		KF228619.1			KF228665.1
		KF228620.1			KF228666.1
		KF228621.1			KF228667.1
		KF228622.1			KF302446.1
		KF228623.1			KF302459.1
		KF228624.1			HM236179.1
		KF228625.1			HM236181.1
Appenninica (Italy)	29	KF228626.1			JN574160.1
		KF228627.1			JN574161.1
		KF228628.1			JN574162.1
		KF228629.1			JN574163.1
		KF228630.1			JN574164.1
		KF228631.1			JN574165.1
		KF228632.1			JN574166.1
		KF228633.1			JN574167.1
		KF228634.1			KF677238.1
		KF228636.1			KF677239.1
		KF228637.1			KF677240.1
		KF228638.1			KF677241.1
		KF228639.1			KF677242.1
		KF302450.1			KF677243.1
		KF302451.1			KF677244.1
		HM236182.1	Morkaraman (Turkey)	36	KF677245.1
Awassi (Pakistan)	3	JX235859.1			KF677246.1
		JX235861.1			KF677247.1
		DQ491625.1			KF677248.1
		DQ491626.1			KF677249.1
		DQ491629.1			KF677250.1
		DQ491647.1			KF677251.1
		DQ491653.1			KF677252.1
		DQ491660.1			KF677253.1
		DQ491661.1			KF677254.1
		DQ491663.1			KF677255.1
Churra Badana (Portugal)	18	DQ491671.1			KF677256.1
		DQ491683.1			KF677257.1
		DQ491684.1			KF677258.1
		DQ491685.1			KF677259.1

		DQ491688.1			KF677260.1
		DQ491690.1			KF677261.1
		DQ491692.1			KF677262.1
		DQ491704.1			KF677263.1
		DQ491721.1	Norduz (Turkey)	13	KF677193.1
		DQ491726.1			KF677199.1
Baruwal (Nepal)	27	KR610850.1			KF677200.1
		KR610851.1			KF677201.1
		KR610852.1			KF677202.1
		KR610853.1			KF677203.1
		KR610854.1			KF677204.1
		KR610855.1			KF677205.1
		KR610856.1			KF677206.1
		KR610857.1			KF677207.1
		KR610858.1			KF677208.1
		KR610859.1			KF677209.1
		KR610860.1			KF677210.1
		KR610861.1	Saloia (Portugal)	30	DQ491579.1
		KR610862.1			DQ491597.1
		KR610863.1			DQ491599.1
		KR610864.1			DQ491604.1
		KR610865.1			DQ491608.1
		KR610866.1			DQ491610.1
		KR610867.1			DQ491615.1
		KR610868.1			DQ491617.1
		KR610869.1			DQ491622.1
		KR610870.1			DQ491623.1
		KR610871.1			DQ491627.1
		KR610872.1			DQ491628.1
		KR610873.1			DQ491630.1
		KR610874.1			DQ491632.1
		KR610875.1			DQ491634.1
		KR610876.1			DQ491637.1
Latxa Blonde Face (Spain)	31	KT879022.1			DQ491644.1
		KT879030.1			DQ491648.1
		KT879036.1			DQ491669.1
		KT879040.1			DQ491670.1
		KT879042.1			DQ491676.1
		KT879043.1			DQ491677.1
		KT879046.1			DQ491679.1
		KT879083.1			DQ491691.1
		KT879085.1			DQ491693.1
		KT879095.1			DQ491694.1
		KT879101.1			DQ491697.1
		KT879118.1			DQ491703.1
		KT879126.1			DQ491710.1
		KT879134.1			DQ491715.1
		KT879138.1			KF228824.1
		KT879141.1			KF228825.1
		KT879147.1			KF228826.1

		KT879148.1	Sarda (Italy)	30	KF228827.1
		KT879149.1			KF228828.1
		KT879150.1			KF228829.1
		KT879151.1			KF228830.1
		KT879152.1			KF228831.1
		KT879153.1			KF228832.1
		KT879155.1			KF228833.1
		KT879169.1			KF228834.1
		KT879170.1			KF228835.1
		KT879171.1			KF228836.1
		KT879172.1			KF228837.1
		KT879173.1			KF228838.1
		KT879184.1			KF228839.1
		KT879185.1			KF228840.1
Latxa Black Face (Spain)	31	KT879022.1			KF228841.1
		KT879038.1			KF228842.1
		KT879073.1			KF228843.1
		KT879099.1			KF228844.1
		KT879134.1			KF228845.1
		KT879136.1			KF228846.1
		KT879146.1			KF228847.1
		KT879154.1			KF228848.1
		KT879156.1			KF228849.1
		KT879157.1			KF228850.1
		KT879158.1			KF228851.1
		KT879159.1			KF228852.1
		KT879160.1			KF228853.1
		KT879161.1	Sopravissana (Italy)	30	KF228586.1
		KT879162.1			KF228587.1
		KT879163.1			KF228588.1
		KT879164.1			KF228589.1
		KT879165.1			KF228590.1
		KT879166.1			KF228591.1
		KT879167.1			KF228592.1
		KT879168.1			KF228593.1
		KT879174.1			KF228594.1
		KT879175.1			KF228595.1
		KT879176.1			KF228596.1
		KT879178.1			KF228597.1
		KT879179.1			KF228598.1
		KT879180.1			KF228599.1
		KT879181.1			KF228600.1
		KT879182.1			KF228601.1
		KT879183.1			KF228602.1
		KT879186.1			KF228603.1
		DQ491706.1			KF228604.1
		DQ491581.1			KF228605.1
		DQ491583.1			KF228606.1
		DQ491584.1			KF228607.1
		DQ491588.1			KF228608.1

		DQ491596.1			KF228609.1
		DQ491609.1			KF228610.1
		DQ491614.1			KF228611.1
		DQ491620.1			KF302449.1
		DQ491624.1			KF302454.1
		DQ491631.1			KF302456.1
		DQ491636.1			KF302458.1
Churra de Terra Quente (Portugal)	25	DQ491638.1			KF228823.1
		DQ491640.1			KF228822.1
		DQ491645.1			KF228821.1
		DQ491650.1			KF228820.1
		DQ491666.1			KF228819.1
		DQ491672.1			KF228818.1
		DQ491673.1			KF228817.1
		DQ491674.1			KF228816.1
		DQ491696.1			KF228815.1
		DQ491701.1			KF228814.1
		DQ491705.1			KF228813.1
		DQ491707.1			KF228812.1
		DQ491716.1	Spanish Merino (Spain)	26	KF228811.1
		DQ491595.1			KF228810.1
		DQ491602.1			KF228809.1
		DQ491611.1			KF228808.1
		DQ491612.1			KF228807.1
		DQ491618.1			KF228806.1
		DQ491619.1			KF228805.1
		DQ491621.1			KF228804.1
		DQ491655.1			KF228803.1
		DQ491657.1			KF228802.1
Campaniça (Portugal)	20	DQ491659.1			KF228801.1
		DQ491662.1			KF228800.1
		DQ491667.1			KF228799.1
		DQ491668.1			KF228798.1
		DQ491680.1			MN229085
		DQ491681.1			MN229086
		DQ491695.1			MN229087
		DQ491698.1			MN229088
		DQ491709.1			MN229089
		DQ491729.1			MN229090
		DQ491731.1			MN229091
		KF228724.1			MN229092
		KF302445.1			MN229093
		KF302452.1			MN229094
		KF302462.1			MN229095
		KF228695.1			MN229096
		KF228696.1			MN229097
		KF228698.1			MN229098
		KF228699.1			MN229099
		KF228700.1	Boujaad (Morocco)	31	MN229100
		KF228703.1			MN229101

		KF228704.1			MN229102
		KF228705.1			MN229103
		KF228706.1			MN229104
		KF228709.1			MN229105
Comisana (Italy)	30	KF228710.1			MN229106
		KF228711.1			MN229107
		KF228713.1			MN229108
		KF228714.1			MN229109
		KF228715.1			MN229110
		KF228717.1			MN229111
		KF228718.1			MN229112
		KF228719.1			MN229113
		KF228720.1			MN229114
		KF228721.1			MN229115
		KF228722.1			MN229116
		KF228723.1			MN229117
		KF228725.1			MN229118
		KF228726.1			MN229119
		KF228727.1			MN229120
		KF228730.1			MN229121
		JN574148.1			MN229122
		JN574149.1			MN229123
		JN574150.1			MN229124
		JN574151.1			MN229125
		JN574152.1			MN229126
		JN574153.1			MN229127
		JN574262.1			MN229128
		JN574263.1			MN229129
		JN574264.1			MN229130
		JN574285.1			MN229131
		JN574286.1	Blanche de Montagne (Morocco)	32	MN229132
		KF677071.1			MN229133
		KF677072.1			MN229134
		KF677073.1			MN229135
		KF677074.1			MN229136
		KF677075.1			MN229137
Daglic (Turkey)	33	KF677076.1			MN229138
		KF677077.1			MN229139
		KF677078.1			MN229140
		KF677079.1			MN229141
		KF677080.1			MN229142
		KF677081.1			MN229143
		KF677082.1			MN229144
		KF677083.1			MN229145
		KF677084.1			MN229146
		KF677085.1			MN229147
		KF677086.1			MN229148
		KF677087.1			MN229149
		KF677088.1			MN229150
		KF677089.1			MN229151

		KF677090.1	Beni-Guil (Morocco)	31	MN229152
		KF677091.1			MN229153
		KF677092.1			MN229154
Garze Tibetan (Tibet)	17	KU681175.1			MN229155
		KU681176.1			MN229156
		KU681177.1			MN229157
		KU681179.1			MN229158
		KU681180.1			MN229159
		KU681181.1			MN229160
		KU681182.1			MN229161
		KU681183.1			MN229162
		KU681184.1			MN229163
		KU681185.1			MN229164
		KU681186.1			MN229165
		KU681187.1			MN229166
		KU681188.1			MN229167
		KU681189.1			MN229168
		KU681190.1			MN229169
		KU681191.1			MN229170
		KU681192.1			MN229171
Gentile De Puglia (Italy)	30	KF228668.1			MN229172
		KF228669.1			MN229173
		KF228670.1			MN229174
		KF228671.1			MN229175
		KF228672.1			MN229176
		KF228673.1			MN229177
		KF228674.1			MN229178
		KF228675.1	D'man (Morocco)	29	MN229179
		KF228676.1			MN229180
		KF228677.1			MN229181
		KF228678.1			MN229182
		KF228679.1			MN229183
		KF228680.1			MN229184
		KF228681.1			MN229185
		KF228682.1			MN229186
		KF228683.1			MN229187
		KF228684.1			MN229188
		KF228685.1			MN229189
		KF228686.1			MN229190
		KF228687.1			MN229191
		KF228688.1			MN229192
		KF228689.1			MN229193
		KF228690.1			MN229194
		KF228691.1			MN229195
		KF228692.1			MN229196
		KF228693.1			MN229197
		KF228694.1			MN229198
		KF302448.1			MN229199
		KF302455.1			MN229200
		KF302457.1			MN229201

		JN573922.1			MN229202
		JN573923.1			MN229203
		JN573924.1			MN229204
		JN573925.1			MN229205
		JN573926.1			MN229206
		JN573927.1			MN229207
		JN573928.1			MN229208
		JN573929.1			MN229209
		JN573930.1			MN229210
		JN573931.1			MN229211
		JN573932.1			MN229212
		JN573933.1			MN229213
		JN573934.1			MN229214
		JN573935.1			MN229215
German Merino (Germany)	29	JN573936.1			MN229216
		JN573937.1			MN229217
		JN573938.1			MN229218
		JN573939.1			MN229219
		JN573940.1			MN229220
		JN573941.1			MN229221
		JN573942.1			MN229222
		JN573943.1			MN229223
		JN573944.1	Sardi (Morocco)	33	MN229224
		JN573945.1			MN229225
		JN573946.1			MN229226
		JN573947.1			MN229227
		JN573948.1			MN229228
		JN574174.1			MN229229
		JN574209.1			MN229230
		KF677119.1			MN229231
		KF677120.1			MN229232
		KF677121.1			MN229233
		KF677122.1			MN229234
		KF677123.1			MN229235
		KF677124.1			MN229236
		KF677125.1			MN229237
Herik (Turkey)	15	KF677126.1			MN229238
		KF677127.1			MN229239
		KF677128.1			MN229240
		KF677129.1			MN229241
		KF677130.1			MN229242
		KF677131.1			MN229243
		KF677132.1			MN229244
		KF677133.1			MN229245
		DQ852282.1			MN229246
		DQ852283.1			MN229247
		DQ852284.1			MN229248
		DQ852285.1			MN229249
		DQ852286.1			MN229250
Karakas (Turkey)	11	DQ852287.1			MN229251

		DQ852288.1			MN229252
		DQ852289.1			MN229253
		HM236176.1			MN229254
		HM236177.1			MN229255
		HM236178.1			MN229256
		JN574154.1			MN229257
		JN574155.1			MN229258
		JN574156.1	Timahdite (Morocco)	37	MN229259
		JN574157.1			MN229260
		JN574158.1			MN229261
		JN574159.1			MN229262
		JN574265.1			MN229263
		JN574266.1			MN229264
		JN574267.1			MN229265
		JN574268.1			MN229266
		JN574269.1			MN229267
		KF677024.1			MN229268
		KF677025.1			MN229269
		KF677026.1			MN229270
Karayaka (Turkey)	30	KF677028.1			MN229271
		KF677029.1			MN229272
		KF677030.1			MN229273
		KF677031.1			MN229274
		KF677032.1			MN229275
		KF677033.1			MN229276
		KF677034.1			MN229277
		KF677035.1			
		KF677036.1			
		KF677037.1			
		KF677038.1			
		MG209458.1			
		MG209461.1			
		MG209462.1			
		MG209465.1			
		MG209466.1			

Access numbers for mutation rate calculation	
Individuals	Access numbers
Hap A 1	HM236174.1
Hap A 2	HM236175.1
Hap B 1	HM236176.1
Hap B 2	HM236177.1
Hap C 1	HM236178.1
Hap C 2	HM236179.1
Hap D 1	HM236180.1
Hap D 2	HM236181.1
Hap E 1	HM236182.1
Hap E 2	HM236183.1
Ovis musimon	HM236184.1
Ovis ammon ammon	HM236188.1
Ovis ammon darwini	AF242347.1
Ovis vignei 1	NC026064.1
Ovis vignei 2	HM236186.1
Ovis dalli	NC039432.1
Ovis nivicola	NC039431.1
Ovis orientalis	HM042854.1

2. Indices of genetic diversity

	N	S	P	Sg	H	Hd	v(Hd)	Std (Hd)	π	v(π)	Std (π)	Fu-Li's F*	p	Fu-Li's D*	p	Fu's Fs	p	Tajima D	p	Shannon	Haplogroup membership				
																					A	B	C	D	E
Blanche de Montagne	32	44	16	28	24	0.97	0.0004	0.0190	0.0133	0.000003	0.0016	-3.016	*	-2.943	*	-13.24	***	-1.766	NS	0.139	0.000	96.875	3.125	0.000	0.000
Boujaad	31	91	30	61	29	0.994	0.0001	0.0110	0.0226	0.000005	0.0023	-3.280	**	-3.105	*	-18.224	***	-2.111	*	0.239	0.000	93.548	6.452	0.000	0.000
Beni-Guil	31	66	24	42	29	0.996	0.0001	0.0090	0.0164	0.000007	0.0027	-2.931	*	-2.741	*	-22.713	***	-1.953	*	0.143	3.226	96.774	0.000	0.000	0.000
D'man	29	41	14	27	21	0.963	0.0005	0.0230	0.0151	0.000001	0.0012	-0.880	NS	-0.634	NS	-7.424	*	-1.744	NS	0.000	0.000	100.000	0.000	0.000	0.000
Sardi	33	76	42	34	30	0.994	0.0001	0.0090	0.0203	0.000010	0.0032	-1.821	NS	-1.457	NS	-18.582	***	-1.692	NS	0.229	6.060	93.940	0.000	0.000	0.000
Timahdite	37	56	27	29	32	0.988	0.0001	0.0110	0.0215	0.000008	0.0028	-2.502	NS	-2.242	NS	-25.845	***	-1.855	*	0.281	0.000	91.892	8.108	0.000	0.000
Churra Algarvia	30	76	27	49	20	0.968	0.0003	0.0160	0.0207	0.000012	0.0035	-1.046	NS	-0.679	NS	-2.651	NS	-1.300	NS	0.389	3.333	90.000	6.667	0.000	0.000
Churra Badana	18	39	24	15	15	0.98	0.0006	0.0240	0.0142	0.000002	0.0013	-0.671	NS	-0.417	NS	-4.627	*	-0.970	NS	0.000	0.000	1.000	0.000	0.000	0.000
Latxa Blonde face	31	44	19	25	24	0.972	0.0004	0.0200	0.0092	0.000001	0.0181	-2.420	NS	-2.175	NS	-15.157	***	-1.800	NS	0.000	0.000	1.000	0.000	0.000	0.000
Latxa Black face	31	69	31	38	28	0.994	0.0010	0.0100	0.0147	0.000006	0.0024	-2.393	NS	-2.126	NS	-18.069	***	-1.817	*	0.143	3.226	96.774	0.000	0.000	0.000
Churra de Terra Quente	25	78	28	50	23	0.993	0.0002	0.0130	0.0178	0.000012	0.0035	-2.570	NS	-2.315	NS	-11.269	***	-1.909	*	0.168	4.000	96.000	0.000	0.000	0.000
Campaniça	20	46	21	25	18	0.989	0.0004	0.0190	0.0133	0.000001	0.0012	-1.766	NS	-1.483	NS	-8.782	***	-1.590	NS	0.000	0.000	100.000	0.000	0.000	0.000
German Merino	29	70	25	45	29	1	0.0001	0.0090	0.0181	0.000016	0.0040	-1.198	NS	-0.660	NS	-23.981	***	-1.738	NS	0.000	0.000	100.000	0.000	0.000	0.000
Saloia	30	64	39	25	28	0.995	0.0001	0.0100	0.0174	0.000014	0.0037	-1.357	NS	-0.924	NS	-18.953	***	-1.604	NS	0.245	6.667	93.333	0.000	0.000	0.000
Spanish Merino	26	62	24	38	26	1	0.0001	0.0110	0.0139	0.000001	0.0010	-2.528	NS	-2.267	NS	-22.036	***	-1.898	NS	0.000	0.000	100.000	0.000	0.000	0.000
Appenninica	29	58	30	28	22	0.98	0.0002	0.0140	0.0144	0.000001	0.0011	-1.828	NS	-1.527	NS	-8.197	**	-1.594	NS	0.000	0.000	100.000	0.000	0.000	0.000
Comisana	30	68	51	17	20	0.94	0.0010	0.0320	0.0328	0.000012	0.0035	0.176	NS	0.002	NS	-0.655	NS	0.446	NS	0.580	26.667	73.333	0.000	0.000	0.000
Gentile de Puglia	30	61	29	32	28	0.995	0.0001	0.0100	0.0147	0.000001	0.0011	-2.178	NS	-1.930	NS	-19.863	***	-1.669	NS	0.000	0.000	100.000	0.000	0.000	0.000
Lacaune of Italy	30	58	28	30	30	1	0.0001	0.0090	0.0125	0.000001	0.0009	-2.158	NS	-1.814	NS	-29.76	***	-1.854	*	0.000	0.000	100.000	0.000	0.000	0.000
Merinizzata Italiana	30	79	39	40	26	0.989	0.0002	0.0130	0.0172	0.000008	0.0028	-2.125	NS	-1.773	NS	-12.742	***	-1.847	*	0.146	3.333	96.667	0.000	0.000	0.000
Sarda	30	48	25	23	22	0.977	0.0002	0.0140	0.0126	0.000001	0.0010	-1.723	NS	-1.455	NS	-8.867	***	-1.470	NS	0.000	0.000	100.000	0.000	0.000	0.000
Sopravissana	30	67	29	38	29	0.998	0.0001	0.0090	0.0149	0.000001	0.0008	-2.436	NS	-2.189	NS	-22.828	***	-1.802	NS	0.000	0.000	100.000	0.000	0.000	0.000
Akkaraman	18	86	57	29	18	1	0.0003	0.0190	0.0450	0.000009	0.0029	0.000	NS	-0.166	NS	-4.665	*	0.399	NS	-	-	-	-	-	-
Baruwal	27	67	25	42	11	0.883	0.0013	0.0360	0.0251	0.000023	0.0048	-0.721	NS	-0.707	NS	4.523	NS	-0.414	NS	-	-	-	-	-	-
Daglic	33	92	56	36	31	0.996	0.0001	0.0090	0.0500	0.000004	0.0020	-0.901	NS	-1.147	NS	-10.721	***	-0.011	NS	-	-	-	-	-	-
Garze Tibetan	17	66	48	18	11	0.912	0.0026	0.0510	0.0350	0.000026	0.0321	0.265	NS	0.167	NS	2.256	NS	0.379	NS	-	-	-	-	-	-
Herik	15	69	48	21	14	0.99	0.0008	0.0280	0.0365	0.000014	0.0350	0.167	NS	0.129	NS	-2.089	NS	0.187	NS	-	-	-	-	-	-
Karakas	11	60	54	6	5	0.818	0.0068	0.0830	0.0412	0.000026	0.0051	1.255	NS	1.204	NS	8.269	NS	0.831	NS	-	-	-	-	-	-
Karayaka	30	73	44	29	27	0.993	0.0001	0.0110	0.0510	0.000006	0.0025	-0.831	NS	-1.039	NS	-8.552	*	-0.026	NS	-	-	-	-	-	-
Morkaraman	36	89	58	31	32	0.992	0.0001	0.0090	0.0493	0.000003	0.0018	-0.532	NS	-0.765	NS	-9.298	*	0.150	NS	-	-	-	-	-	-
Norduz	13	78	52	26	13	1	0.0009	0.0300	0.0399	0.000030	0.0055	-0.036	NS	0.035	NS	-2.707	NS	-0.212	NS	-	-	-	-	-	-
Moroccan group 1	119	107	56	51	87	0.99	0.00001	0.003	0.01682	0.0000008	0.00088	-3.77092	**	-3.9896	**	-34.052	***	-2.05071	*	-	-	-	-	-	-
Moroccan group 2	32	68	29	39	32	1	0.00006	0.008	0.02005	0.0000018	0.00135	-2.73125	*	-2.48107	.1>p>0.0	-30.699	***	-1.96395	*	-	-	-	-	-	-
Iberian group	119	134	73	61	105	0.997	0.000003	0.0017	0.01372	0.0000008	0.00091	-3.61814	**	-3.66164	**	-152.842	***	-2.19728	**	-	-	-	-	-	-
Italian group	119	139	94	45	104	0.998	0.000002	0.0014	0.01643	0.0000013	0.00112	-2.4585	*	-2.02597	.1>p>0.0	-146.043	***	-2.07911	*	-	-	-	-	-	-

3. Newik-Extra results

Matrix of Numbers of Similarities between breeds

	Boujaad	ch de Mont	Beni-Guil	D'man	Sardi	Timahdite	hurra Algarv	hurra Badan	xa Blonde F	txa Black Fa	Campaniça	a de Terra Q	erman Meri	Saloia	panish Merin	Appenninica	Comisana	ntile de Pug	caune of Ita	inizzata Ital	Sarda	Sopravissana	Akkaraman	Awassi	Baruwal	Daglic	arze Tibeta	Herik	Karakas	Karayaka	Morkaraman	Norduz
Boujaad	14	0	1	0	2	3	2	0	0	0	0	0	0	0	0	0	0	0	1	1	0	0	1	0	0	0	0	1	0	0	0	0
Blanch de Montagne	0	8	0	16	5	6	0	1	0	2	0	1	0	1	0	0	0	2	0	1	0	0	0	0	0	0	0	1	0	0	0	0
Beni-Guil	1	0	4	3	3	2	0	0	1	0	0	0	2	0	0	0	0	0	1	0	1	0	1	0	0	0	0	0	0	0	0	0
D'man	0	16	3	46	13	20	0	0	0	0	0	0	0	0	0	0	0	0	0	0	0	0	0	0	0	0	0	0	0	0	0	0
Sardi	2	5	3	13	10	3	1	0	0	0	0	2	1	0	1	1	1	0	0	2	0	0	0	0	0	0	0	0	0	1	0	0
Timahdite	3	6	2	20	3	14	1	0	2	1	0	0	0	1	0	0	2	1	2	1	0	1	0	0	0	0	0	0	0	0	0	0
Churra Algarvia	2	0	0	0	1	1	32	1	0	0	0	1	0	1	0	0	0	0	0	0	0	0	0	0	0	0	0	0	0	0	0	1
Churra Badana	0	1	0	0	0	0	1	6	1	0	0	2	0	2	0	0	0	0	0	0	0	1	0	0	0	0	0	0	0	0	0	0
Latxa Blonde Face	0	0	1	0	0	2	0	1	6	3	0	0	0	3	0	2	0	0	2	0	0	0	0	0	0	0	1	0	0	0	0	0
Latxa Black Face	0	2	0	0	0	1	0	0	3	8	1	0	0	0	1	1	0	1	0	0	0	0	0	0	0	0	1	0	0	0	0	1
Campaniça	0	0	0	0	0	0	0	0	0	1	4	1	1	1	0	0	1	1	1	0	0	1	0	0	0	0	0	0	0	2	0	0
Churra de Terra Quente	0	1	0	0	2	0	1	2	0	0	1	6	0	2	1	0	0	0	0	1	0	0	0	0	0	0	0	0	0	0	0	1
German Merino	0	0	2	0	1	0	0	0	0	0	1	0	6	0	1	1	0	1	2	1	1	0	0	0	0	2	0	0	0	0	1	0
Saloia	0	1	0	0	0	1	1	2	3	0	1	2	0	4	0	0	1	1	2	2	0	1	0	0	0	0	0	1	0	0	0	0
Spanish Merino	0	0	0	0	1	0	0	0	0	1	0	1	1	0	0	0	0	2	1	0	0	0	0	0	0	0	0	1	0	0	0	0
Appenninica	0	0	0	0	1	0	0	0	2	1	0	0	1	0	0	12	0	0	2	2	1	1	0	0	0	0	1	0	0	0	0	0
Comisana	0	0	0	0	1	2	0	0	0	0	1	0	0	1	0	0	54	1	2	0	1	1	0	0	0	0	0	0	0	0	0	0
Gentile de Puglia	0	2	0	0	0	1	0	0	0	1	1	0	1	1	2	0	1	6	2	1	1	4	0	0	0	0	0	0	0	0	0	0
Lacaune of Italy	1	0	1	0	0	2	0	0	2	0	1	0	2	2	1	2	2	2	4	1	0	0	0	0	0	0	0	0	0	0	1	0
Merinizzata Italiana	1	1	0	0	2	1	0	0	0	0	0	1	1	2	0	2	0	1	1	12	1	0	0	0	0	0	0	0	0	0	0	0
Sarda	0	0	1	0	0	0	0	0	0	0	0	0	1	0	0	1	1	1	0	1	14	1	0	0	0	1	0	0	0	0	1	0
Sopravissana	0	0	0	0	0	1	0	1	0	0	1	0	0	1	0	1	1	4	0	0	1	6	0	0	1	0	0	0	0	0	0	0
Akkaraman	1	0	1	0	0	0	0	0	0	0	0	0	0	0	0	0	0	0	0	0	0	0	2	0	0	1	1	0	0	0	2	1
Awassi	0	0	0	0	0	0	0	0	0	0	0	0	0	0	0	0	0	0	0	0	0	0	0	0	0	0	0	0	0	0	1	0
Baruwal	0	0	0	0	0	0	0	0	0	0	0	0	0	0	0	0	0	0	0	0	0	1	0	0	48	0	0	0	1	0	1	0
Daglic	0	0	0	0	0	0	0	0	0	0	0	0	2	0	0	0	0	0	0	0	1	0	1	0	0	2	4	1	0	6	6	4
Garze Tibetan	0	0	0	0	0	0	0	0	1	1	0	0	0	0	0	1	0	0	0	0	0	0	1	0	0	4	14	4	0	0	13	4
Herik	1	1	0	0	0	0	0	0	0	0	0	0	0	1	1	0	0	0	0	0	0	0	0	0	0	1	4	2	0	1	3	1
Karakas	0	0	0	0	0	0	0	0	0	0	0	0	0	0	0	0	0	0	0	0	0	0	0	0	1	0	0	0	8	0	0	0
Karayaka	0	0	0	0	1	0	0	0	0	0	2	0	0	0	0	0	0	0	0	0	0	0	0	0	0	6	0	1	0	6	2	1
Morkaraman	0	0	0	0	0	0	0	0	0	0	0	0	1	0	0	0	0	0	1	0	1	0	2	1	1	6	13	3	0	2	8	4
Norduz	0	0	0	0	0	0	1	0	0	1	0	1	0	0	0	0	0	0	0	0	0	0	1	0	0	4	4	1	0	1	4	0

Matrix of proportions of Similarities between breeds

	Boujaad	ch de Mont	Beni-Guil	D'man	Sardi	Timahdite	hurra Algarv	hurra Badan	xa Blonde F	txa Black Fa	Campaniça	a de Terra Q	erman Meri	Saloia	panish Merin	Appenninica	Comisana	ntile de Pug	caune of Ita	inizzata Ital	Sarda	Sopravissana	Akkaraman	Awassi	Baruwal	Daglic	arze Tibeta	Herik	Karakas	Karayaka	Morkaraman	Norduz
Boujaad	0.54	0.00	0.05	0.00	0.04	0.05	0.05	0.00	0.00	0.00	0.00	0.00	0.00	0.00	0.00	0.00	0.00	0.00	0.04	0.04	0.00	0.00	0.11	0.00	0.00	0.00	0.00	0.06	0.00	0.00	0.00	0.00
Blanch de Montagne	0.00	0.18	0.00	0.16	0.11	0.10	0.00	0.07	0.00	0.10	0.00	0.06	0.00	0.04	0.00	0.00	0.00	0.08	0.00	0.04	0.00	0.00	0.00	0.00	0.00	0.00	0.00	0.06	0.00	0.00	0.00	0.00
Beni-Guil	0.04	0.00	0.21	0.03	0.07	0.03	0.00	0.00	0.05	0.00	0.00	0.00	0.10	0.00	0.00	0.00	0.00	0.00	0.04	0.00	0.04	0.00	0.11	0.00	0.00	0.00	0.00	0.00	0.00	0.00	0.00	0.00
D'man	0.00	0.36	0.16	0.47	0.28	0.33	0.00	0.00	0.00	0.00	0.00	0.00	0.00	0.00	0.00	0.00	0.00	0.00	0.00	0.00	0.00	0.00	0.00	0.00	0.00	0.00	0.00	0.00	0.00	0.00	0.00	0.00
Sardi	0.08	0.11	0.16	0.13	0.22	0.05	0.03	0.00	0.00	0.00	0.00	0.11	0.05	0.00	0.13	0.04	0.02	0.00	0.00	0.08	0.00	0.00	0.00	0.00	0.00	0.00	0.00	0.00	0.00	0.05	0.00	0.00
Timahdite	0.12	0.14	0.11	0.20	0.07	0.23	0.03	0.00	0.10	0.05	0.00	0.00	0.00	0.04	0.00	0.00	0.03	0.04	0.08	0.04	0.00	0.06	0.00	0.00	0.00	0.00	0.00	0.00	0.00	0.00	0.00	0.00
Churra Algarvia	0.08	0.00	0.00	0.00	0.02	0.02	0.80	0.07	0.00	0.00	0.00	0.06	0.00	0.04	0.00	0.00	0.00	0.00	0.00	0.00	0.00	0.00	0.00	0.00	0.00	0.00	0.00	0.00	0.00	0.00	0.00	0.06
Churra Badana	0.00	0.02	0.00	0.00	0.00	0.00	0.03	0.43	0.05	0.00	0.00	0.11	0.00	0.09	0.00	0.00	0.00	0.00	0.00	0.00	0.00	0.06	0.00	0.00	0.00	0.00	0.00	0.00	0.00	0.00	0.00	0.00
Latxa Blonde Face	0.00	0.00	0.05	0.00	0.00	0.03	0.00	0.07	0.29	0.15	0.00	0.00	0.00	0.13	0.00	0.08	0.00	0.00	0.08	0.00	0.00	0.00	0.00	0.00	0.00	0.00	0.02	0.00	0.00	0.00	0.00	0.00
Latxa Black Face	0.00	0.05	0.00	0.00	0.00	0.02	0.00	0.00	0.14	0.40	0.07	0.00	0.00	0.00	0.13	0.04	0.00	0.04	0.00	0.00	0.00	0.00	0.00	0.00	0.00	0.00	0.02	0.00	0.00	0.00	0.00	0.06
Campaniça	0.00	0.00	0.00	0.00	0.00	0.00	0.00	0.00	0.00	0.05	0.29	0.06	0.05	0.04	0.00	0.00	0.02	0.04	0.04	0.00	0.00	0.06	0.00	0.00	0.00	0.00	0.00	0.00	0.00	0.11	0.00	0.00
Churra de Terra Quente	0.00	0.02	0.00	0.00	0.04	0.00	0.03	0.14	0.00	0.00	0.07	0.33	0.00	0.09	0.13	0.00	0.00	0.00	0.00	0.04	0.00	0.00	0.00	0.00	0.00	0.00	0.00	0.00	0.00	0.00	0.00	0.06
German Merino	0.00	0.00	0.11	0.00	0.02	0.00	0.00	0.00	0.00	0.00	0.07	0.00	0.30	0.00	0.13	0.04	0.00	0.04	0.08	0.04	0.04	0.00	0.00	0.00	0.00	0.07	0.00	0.00	0.00	0.00	0.02	0.00
Saloia	0.00	0.02	0.00	0.00	0.00	0.02	0.03	0.14	0.14	0.00	0.07	0.11	0.00	0.17	0.00	0.00	0.02	0.04	0.08	0.08	0.00	0.06	0.00	0.00	0.00	0.00	0.00	0.06	0.00	0.00	0.00	0.00
Spanish Merino	0.00	0.00	0.00	0.00	0.02	0.00	0.00	0.00	0.00	0.05	0.00	0.06	0.05	0.00	0.00	0.00	0.00	0.08	0.04	0.00	0.00	0.00	0.00	0.00	0.00	0.00	0.00	0.06	0.00	0.00	0.00	0.00
Appenninica	0.00	0.00	0.00	0.00	0.02	0.00	0.00	0.00	0.10	0.05	0.00	0.00	0.05	0.00	0.00	0.50	0.00	0.00	0.08	0.08	0.04	0.06	0.00	0.00	0.00	0.00	0.02	0.00	0.00	0.00	0.00	0.00
Comisana	0.00	0.00	0.00	0.00	0.02	0.03	0.00	0.00	0.00	0.00	0.07	0.00	0.00	0.04	0.00	0.00	0.84	0.04	0.08	0.00	0.04	0.06	0.00	0.00	0.00	0.00	0.00	0.00	0.00	0.00	0.00	0.00
Gentile de Puglia	0.00	0.05	0.00	0.00	0.00	0.02	0.00	0.00	0.00	0.05	0.07	0.00	0.05	0.04	0.25	0.00	0.02	0.25	0.08	0.04	0.04	0.22	0.00	0.00	0.00	0.00	0.00	0.00	0.00	0.00	0.00	0.00
Lacaune of Italy	0.04	0.00	0.05	0.00	0.00	0.03	0.00	0.00	0.10	0.00	0.07	0.00	0.10	0.09	0.13	0.08	0.03	0.08	0.17	0.04	0.00	0.00	0.00	0.00	0.00	0.00	0.00	0.00	0.00	0.00	0.02	0.00
Merinizzata Italiana	0.04	0.02	0.00	0.00	0.04	0.02	0.00	0.00	0.00	0.00	0.00	0.06	0.05	0.09	0.00	0.08	0.00	0.04	0.04	0.46	0.04	0.00	0.00	0.00	0.00	0.00	0.00	0.00	0.00	0.00	0.00	0.00
Sarda	0.00	0.00	0.05	0.00	0.00	0.00	0.00	0.00	0.00	0.00	0.00	0.00	0.05	0.00	0.00	0.04	0.02	0.04	0.00	0.04	0.61	0.06	0.00	0.00	0.00	0.04	0.00	0.00	0.00	0.00	0.02	0.00
Sopravissana	0.00	0.00	0.00	0.00	0.00	0.02	0.00	0.07	0.00	0.00	0.07	0.00	0.00	0.04	0.00	0.04	0.02	0.17	0.00	0.00	0.04	0.33	0.00	0.00	0.02	0.00	0.00	0.00	0.00	0.00	0.00	0.00
Akkaraman	0.04	0.00	0.05	0.00	0.00	0.00	0.00	0.00	0.00	0.00	0.00	0.00	0.00	0.00	0.00	0.00	0.00	0.00	0.00	0.00	0.00	0.00	0.22	0.00	0.00	0.04	0.02	0.00	0.00	0.00	0.05	0.06
Awassi	0.00	0.00	0.00	0.00	0.00	0.00	0.00	0.00	0.00	0.00	0.00	0.00	0.00	0.00	0.00	0.00	0.00	0.00	0.00	0.00	0.00	0.00	0.00	0.00	0.00	0.00	0.00	0.00	0.00	0.00	0.02	0.00
Baruwal	0.00	0.00	0.00	0.00	0.00	0.00	0.00	0.00	0.00	0.00	0.00	0.00	0.00	0.00	0.00	0.00	0.00	0.00	0.00	0.00	0.00	0.06	0.00	0.00	0.94	0.00	0.00	0.00	0.11	0.00	0.02	0.00
Daglic	0.00	0.00	0.00	0.00	0.00	0.00	0.00	0.00	0.00	0.00	0.00	0.00	0.10	0.00	0.00	0.00	0.00	0.00	0.00	0.00	0.04	0.00	0.11	0.00	0.00	0.07	0.09	0.06	0.00	0.32	0.14	0.22
Garze Tibetan	0.00	0.00	0.00	0.00	0.00	0.00	0.00	0.00	0.05	0.05	0.00	0.00	0.00	0.00	0.00	0.04	0.00	0.00	0.00	0.00	0.00	0.00	0.11	0.00	0.00	0.15	0.33	0.25	0.00	0.00	0.30	0.22
Herik	0.04	0.02	0.00	0.00	0.00	0.00	0.00	0.00	0.00	0.00	0.00	0.00	0.00	0.04	0.13	0.00	0.00	0.00	0.00	0.00	0.00	0.00	0.00	0.00	0.00	0.04	0.09	0.13	0.00	0.05	0.07	0.06
Karakas	0.00	0.00	0.00	0.00	0.00	0.00	0.00	0.00	0.00	0.00	0.00	0.00	0.00	0.00	0.00	0.00	0.00	0.00	0.00	0.00	0.00	0.00	0.00	0.00	0.02	0.00	0.00	0.00	0.89	0.00	0.00	0.00
Karayaka	0.00	0.00	0.00	0.00	0.02	0.00	0.00	0.00	0.00	0.00	0.14	0.00	0.00	0.00	0.00	0.00	0.00	0.00	0.00	0.00	0.00	0.00	0.00	0.00	0.00	0.22	0.00	0.06	0.00	0.32	0.05	0.06
Morkaraman	0.00	0.00	0.00	0.00	0.00	0.00	0.00	0.00	0.00	0.00	0.00	0.00	0.05	0.00	0.00	0.00	0.00	0.00	0.04	0.00	0.04	0.00	0.22	1.00	0.02	0.22	0.30	0.19	0.00	0.11	0.19	0.22
Norduz	0.00	0.00	0.00	0.00	0.00	0.00	0.03	0.00	0.00	0.05	0.00	0.06	0.00	0.00	0.00	0.00	0.00	0.00	0.00	0.00	0.00	0.00	0.11	0.00	0.00	0.15	0.09	0.06	0.00	0.05	0.09	0.00

	Boujaad	che de Mont	Beni-Guil	D'man	Sardi	Timahdite
Moroccan	0.769	0.795	0.684	1.000	0.783	0.800
Iberian	0.077	0.114	0.158	0.000	0.109	0.083
Italian	0.077	0.068	0.105	0.000	0.087	0.117
Oriental	0.077	0.023	0.053	0.000	0.022	0.000
Total	1.000	1.000	1.000	1.000	1.000	1.000

4. Genetic distances

NB: Values of the corrected average pair-wise differences between populations (Da) are below the diagonal, and the values of the average number of pair-wise differences between populations, Dxy are above.

Da Dxy	BOUJAAD	CHE DE MONT	BENI-GUIL	DMAN	SARDI	TIMAHDITE	URRA ALGARV	RRA BADA	A BLONDE	XTA BLACK FA	CAMPANICA	A DE TERRA Q	RMAN MERIN	SALOIA	NISH MER	APPENNINICA
BOUJAAD	0	0.01701	0.01898	0.02048	0.02232	0.02051	0.00061	0.0008	0.00022	0.00005	0.0004	0.00042	0.00039	0.00009	0.00012	0.00037
CHE DE MONT	0.0002	0	0.01433	0.01251	0.01659	0.02039	0.00048	0.00054	0.00056	0.00017	0.00043	0.00068	0.00078	0.00026	0.00073	0.0006
BENI-GUIL	0.00017	0.00036	0	0.0148	0.01774	0.0193	0.00092	0.00038	0.00014	0.0002	0.00031	0.00048	-0.00009	0.00008	0.00015	0.00063
DMAN	0.00104	0.00059	0.00007	0	0.01879	0.01702	0.00213	0.00146	0.00111	0.00104	0.00109	0.00152	0.00044	0.00083	0.00078	0.00133
SARDI	0.0004	0.00065	0.00017	0.00106	0	0.02143	0.00055	0.00009	0.0004	0.00009	0.00016	-0.00008	0.00002	0.00013	0.00026	0.0005
TIMAHDITE	0.00004	0.00052	0.00016	0.0004	0.00042	0	0.00062	0.00017	0.00021	0.00029	0.00047	0.00041	0.00039	0.00011	0.00042	0.00011
URRA ALGARV	0.02283	0.01671	0.01926	0.02074	0.02206	0.02266	0	0.018	0.01587	0.01829	0.01757	0.01989	0.01962	0.01887	0.01864	0.0188
HURRA BADAN	0.01983	0.01312	0.01495	0.01674	0.01776	0.01783	0.00058	0	0.01224	0.01475	0.01428	0.01614	0.01577	0.01533	0.01495	0.01516
XTA BLONDE FA	0.01662	0.01066	0.01313	0.0136	0.01544	0.01528	0.0009	0.00053	0	0.01202	0.01144	0.01387	0.0137	0.01277	0.01171	0.01219
XTA BLACK FA	0.01938	0.01221	0.01551	0.01635	0.01789	0.0176	0.00062	0.00034	0.00008	0	0.01397	0.01633	0.01614	0.01473	0.01455	0.01496
CAMPANICA	0.01915	0.01259	0.01566	0.01605	0.01783	0.01866	0.00051	0.0005	0.00016	-0.00003	0	0.01577	0.01565	0.01429	0.01381	0.01431
A DE TERRA Q	0.02123	0.01562	0.01755	0.01829	0.0194	0.02101	0.00063	0.00013	0.00033	0.00009	0.00016	0	0.01729	0.0167	0.01639	0.01674
ERMAN MERIN	0.01999	0.01476	0.01517	0.01591	0.01911	0.01872	0.00063	0.00055	0.00038	0.00019	0.00035	0.00019	0	0.01668	0.01633	0.01634
SALOIA	0.01932	0.01525	0.01469	0.01567	0.01847	0.02069	0.00045	0.00023	0.00029	-0.00012	0.00032	0.00002	-0.00001	0	0.0154	0.01509
PANISH MERIN	0.01876	0.01329	0.0148	0.01533	0.01752	0.01754	0.00106	0.00071	0.00006	0.00007	0.00012	0.00028	0.00024	0.00036	0	0.01437
APPENNINICA	0.0193	0.01336	0.01571	0.0164	0.01818	0.01781	0.001	0.00071	0.00032	0.00027	0.00028	0.00042	0.0008	0.00028	0.00022	0
COMISANA	0.03072	0.02518	0.02533	0.02772	0.02804	0.03031	0.0028	0.00308	0.00429	0.00713	0.00351	0.00267	0.00233	0.00203	0.00394	0.00405
s	0.01957	0.01325	0.01606	0.01633	0.01811	0.01905	0.00044	0.00037	0.0002	-0.00002	-0.00003	0.00014	0.00023	0.00018	0.00014	0.00047
ACAUNE D'Ital	0.01804	0.01255	0.01421	0.01486	0.01685	0.01699	0.00076	0.00042	0.00006	-0.00004	-0.00006	0.00016	0.0001	0.00016	-0.00015	0.00016
RINIZZTA ITALI	0.0205	0.01477	0.01656	0.01762	0.01864	0.01975	0.00045	0.00025	0.00013	-0.00002	0.00006	0.00001	0.00001	-0.00002	0.00008	0.00017
SARDA	0.01893	0.01426	0.01584	0.01635	0.01768	0.01996	0.00085	0.00031	0.00047	0.00042	0.00045	0.0002	0.00088	0.00043	0.0007	0.00067
SOPRAVISSANA	0.02044	0.01349	0.01615	0.01663	0.01827	0.0189	0.00075	0.00047	0.00032	0.00005	0.00002	0.00004	0.00016	0.00023	0.00009	0.00029
AKKARAMAN	0.04654	0.03946	0.0363	0.0444	0.04479	0.04002	0.043	0.0425	0.04215	0.04314	0.04363	0.04434	0.04419	0.04448	0.04485	0.04501
AWASSI	0.04217	0.03869	0.03738	0.04304	0.04184	0.04405	0.03892	0.0383	0.03731	0.03817	0.03896	0.03954	0.03924	0.03885	0.04049	0.04085
BARUWAL	0.04902	0.04088	0.03994	0.04586	0.04527	0.04701	0.0473	0.0452	0.04606	0.04577	0.0475	0.04709	0.04632	0.04566	0.04893	0.04938
DAGLIC	0.0457	0.04089	0.03667	0.04453	0.04637	0.03882	0.04585	0.0451	0.0443	0.04475	0.04552	0.04638	0.04552	0.04583	0.0464	0.04601
GARZE TIBETAN	0.03358	0.02681	0.02566	0.03092	0.0317	0.03045	0.03076	0.0288	0.02752	0.02923	0.0293	0.03062	0.03078	0.03084	0.03029	0.03092
HERIK	0.0347	0.02735	0.02752	0.03185	0.03256	0.03186	0.03226	0.03	0.02922	0.03025	0.03048	0.03189	0.03171	0.03087	0.03167	0.0321
KARAKAS	0.0379	0.03324	0.03013	0.03569	0.03673	0.03605	0.03595	0.035	0.03373	0.03537	0.0355	0.03682	0.03684	0.03723	0.03569	0.03631
KARAYAKA	0.04867	0.04498	0.03879	0.0476	0.04939	0.04238	0.04975	0.0472	0.0466	0.04662	0.04799	0.04947	0.04733	0.04753	0.04902	0.04895
MORKARAMAN	0.04959	0.04462	0.03838	0.04893	0.05086	0.04108	0.05083	0.0501	0.0498	0.05015	0.05135	0.05208	0.05117	0.05072	0.05178	0.05155
NORDUZ	0.04094	0.03432	0.03173	0.03856	0.03961	0.03594	0.03683	0.0364	0.03532	0.03705	0.03687	0.03817	0.03868	0.03861	0.03832	0.03861

Da Dxy	COMISANA	NTILE DE PUG	CAUNE D'Ital	NIZZTA ITAL	SARDA	OPRAVISSANA	KKARAMAN	AWASSI	BARUWAL	DAGLIC	RZE TIBET	HERIK	KARAKAS	KARAYAKA	MORKARAMAN	NORDUZ
BOUJAAD	0.00348	0.00023	0.0001	0.00031	0.00079	0.00035	0.01239	0.00975	0.02503	0.01142	0.0043	0.00489	0.00633	0.01182	0.01603	0.00916
CHE DE MONT	0.00339	0.00019	0.00054	0.0003	0.0008	0.00053	0.0121	0.01154	0.02241	0.01079	0.0037	0.00434	0.00635	0.01105	0.01531	0.00884
BENI-GUIL	0.00317	0.00021	0.00014	0.00033	0.00083	0.00042	0.00969	0.01649	0.02082	0.00981	0.00333	0.0042	0.00548	0.00947	0.01226	0.00706
DMAN	0.00469	0.00092	0.00073	0.00125	0.00183	0.00094	0.01458	0.01303	0.02582	0.01384	0.0058	0.00612	0.0082	0.01403	0.01874	0.01111
SARDI	0.00203	0.00015	0.00014	-0.00013	0.00045	0.00002	0.01203	0.00926	0.02229	0.01147	0.00349	0.00384	0.00607	0.01104	0.01665	0.00915
TIMAHDITE	0.00366	0.00031	0.00021	0.00016	0.00097	0.00031	0.01045	0.01536	0.02392	0.00914	0.00324	0.00456	0.00562	0.00966	0.01189	0.00671
URRA ALGARV	0.0298	0.01839	0.01761	0.01967	0.01776	0.0188	0.01011	0.00667	0.02422	0.00975	0.00268	0.00375	0.0046	0.0113	0.01482	0.00655
HURRA BADAN	0.02674	0.01497	0.01393	0.01614	0.01389	0.01518	0.01286	0.00927	0.02537	0.01267	0.00393	0.00467	0.00702	0.01232	0.01838	0.00933
XTA BLONDE FA	0.02537	0.01222	0.01098	0.01343	0.01145	0.01244	0.015	0.01079	0.0287	0.01506	0.00516	0.00635	0.0835	0.016	0.02074	0.01077
XTA BLACK FA	0.04645	0.01483	0.01371	0.0161	0.01422	0.015	0.01328	0.00896	0.02571	0.01271	0.00417	0.00467	0.00712	0.01289	0.0183	0.0098
CAMPANICA	0.02649	0.01416	0.01297	0.01554	0.0136	0.01431	0.01429	0.01026	0.02804	0.01416	0.00479	0.00556	0.0078	0.01477	0.02008	0.01016
A DE TERRA Q	0.0282	0.01661	0.01553	0.01776	0.01565	0.01663	0.01289	0.00873	0.02544	0.01259	0.00397	0.00471	0.00698	0.01284	0.01849	0.00933
ERMAN MERIN	0.02859	0.01623	0.0152	0.01773	0.0164	0.016	0.01226	0.00771	0.02458	0.0114	0.00332	0.00368	0.00635	0.01131	0.01682	0.0091
SALOIA	0.02746	0.01532	0.01431	0.01693	0.01519	0.01507	0.0129	0.00707	0.02382	0.01115	0.00326	0.00366	0.0062	0.01078	0.01654	0.00934
PANISH MERIN	0.02732	0.01444	0.01305	0.01566	0.01397	0.0145	0.01494	0.01105	0.02895	0.01461	0.00512	0.00596	0.00797	0.01531	0.02025	0.0109
APPENNINICA	0.02762	0.01498	0.01357	0.01597	0.01415	0.01491	0.01492	0.01122	0.02921	0.01449	0.00555	0.0062	0.0084	0.01512	0.02022	0.011
COMISANA	0	0.0271	0.02629	0.02776	0.02619	0.02717	0.00713	0.00121	0.0161	0.00566	0.00157	0.00014	0.00377	0.00334	0.00968	0.00743
s	0.00337	0	0.01353	0.01603	0.01401	0.01473	0.01379	0.00963	0.02769	0.0134	0.00452	0.00529	0.00711	0.01388	0.0192	0.00989
ACAUNE D'Ital	0.00366	-0.00004	0	0.0148	0.01304	0.01364	0.01457	0.0106	0.02606	0.01425	0.0049	0.00567	0.00776	0.01472	0.01984	0.01054
RINIZZTA ITALI	0.00276	0.00008	-0.00005	0	0.01533	0.016	0.01322	0.00878	0.02606	0.01283	0.00408	0.00466	0.00678	0.01309	0.0183	0.00968
SARDA	0.0035	0.00037	0.0005	0.00041	0	0.01424	0.01471	0.01038	0.0281	0.01416	0.00497	0.00573	0.00783	0.01453	0.02006	0.01053
SOPRAVISSANA	0.00334	-0.00004	-0.00003	-0.00006	0.0005	0	0.01416	0.01024	0.02795	0.01367	0.00478	0.00531	0.00768	0.01406	0.01973	0.01022
AKKARAMAN	0.04645	0.04404	0.04372	0.04475	0.04393	0.04451	0	0.04185	0.04571	0.0488	0.04153	0.04191	0.04357	0.05483	0.04833	0.04208
AWASSI	0.04005	0.03941	0.03928	0.03984	0.03913	0.04012	-0.0026	0	0.0473	0.0456	0.0378	0.03694	0.04068	0.04872	0.04595	0.04252
BARUWAL	0.042	0.04801	0.04758	0.04767	0.04739	0.04839	0.01043	0.02422	0	0.0493	0.04185	0.03871	0.04627	0.04566	0.04817	0.05034
DAGLIC	0.0479	0.04543	0.04517	0.04668	0.04577	0.04537	-0.00095	-0.00394	0.01002	0	0.04637	0.04531	0.04865	0.05317	0.04944	0.04802
GARZE TIBETAN	0.03615	0.03004	0.02932	0.03088	0.02946	0.03041	0.00124	-0.00183	0.01177	0.00036	0	0.03476	0.03791	0.05027	0.04808	0.03797
HERIK	0.03528	0.03134	0.03062	0.032	0.03074	0.03147	0.00149	-0.00271	0.00776	0.00047	-0.00095	0	0.03911	0.04751	0.0473	0.04024
KARAKAS	0.04039	0.03518	0.03471	0.0361	0.03485	0.03586	0.00023	-0.00219	0.01275	-0.00035	-0.00087	0.0002	0	0.05448	0.04935	0.03979
KARAYAKA	0.04868	0.04811	0.04772	0.04875	0.04841	0.04831	0.00201	-0.00515	0.00472	0.00064	0.00156	-0.0005	0.00253	0	0.05436	0.0558
MORKARAMAN	0.05176	0.05111	0.0504	0.05196	0.0512	0.05141	-0.00113	-0.00271	0.00965	-0.00049	0.00281	0.00281	0.00104	0.00179	0	0.04853
NORDUZ	0.04425	0.03765	0.03721	0.03872	0.03726	0.03809	-0.0004	0.00071	0.01768	0.00001	0.00031	0.00246	-0.00122	0.00555	0.00108	0

5. Network count

Morrocan Group 1+2						Iberian group						Italian Group						Moroccan Group 2						Oriental group (Haplogroups A, C D E)					
nche de Monta	Beni-Guil	D'man	Sardi	Timahdite	Boujaad	nche de Monta	Beni-Guil	D'man	Sardi	Timahdite	Boujaad	nche de Monta	Beni-Guil	D'man	Sardi	Timahdite	Boujaad	nche de Monta	Beni-Guil	D'man	Sardi	Timahdite	Boujaad	nche de Monta	Beni-Guil	D'man	Sardi	Timahdite	Boujaad
BLM01	BNG01	DMAN01	SRDI02	TIM01	BJD15	BLM28	0	0	SRDI01	TIM07	BJD02	0	BNG07	DMAN25	SRDI05	TIM14	BJD01	0	BNG02	DMAN05	SRDI21	TIM20	BJD03	BLM02	BNG26	0	SRDI04	TIM19	BJD17
BLM03	BNG03	DMAN02	SRDI03	TIM02	BJD22				SRDI14	TIM09	BJD05			DMAN26	SRDI06	TIM17	BJD04		BNG11	DMAN14	SRDI22	TIM22	BJD11				SRDI12	TIM34	BJD31
BLM04	BNG04	DMAN03	SRDI08	TIM03	BJD26				SRDI19	TIM12	BJD07				SRDI07	TIM35	BJD06		BNG15	DMAN15	SRDI23	TIM23	BJD13						
BLM05	BNG05	DMAN04	SRDI10	TIM04	BJD27						BJD08				SRDI09		BJD10		BNG29	DMAN16	SRDI24	TIM24	BJD23						
BLM06	BNG06	DMAN06	SRDI11	TIM05	BJD28						BJD09				SRDI32		BJD16			DMAN19	SRDI26	TIM25	BJD24						
BLM07	BNG08	DMAN07	SRDI13	TIM06	BJD29						BJD12						BJD18			DMAN21	SRDI27	TIM27	BJD25						
BLM08	BNG09	DMAN08	SRDI15	TIM08							BJD14						BJD19				SRDI29	TIM33	BJD30						
BLM09	BNG10	DMAN09	SRDI16	TIM10													BJD20				SRDI33								
BLM10	BNG12	DMAN10	SRDI17	TIM11													BJD21												
BLM11	BNG13	DMAN11	SRDI18	TIM13																									
BLM12	BNG14	DMAN12	SRDI20	TIM15																									
BLM13	BNG16	DMAN13	SRDI25	TIM16																									
BLM14	BNG17	DMAN17	SRDI28	TIM18																									
BLM15	BNG18	DMAN18	SRDI30	TIM21																									
BLM16	BNG19	DMAN20	SRDI31	TIM26																									
BLM17	BNG20	DMAN22		TIM28																									
BLM18	BNG21	DMAN23		TIM29																									
BLM19	BNG22	DMAN24		TIM30																									
BLM20	BNG23	DMAN27		TIM31																									
BLM21	BNG24	DMAN28		TIM32																									
BLM22	BNG25	DMAN29		TIM36																									
BLM23	BNG27			TIM37																									
BLM24	BNG28																												
BLM25	BNG30																												
BLM26	BNG31																												
BLM27																													
BLM29																													
BLM30																													
BLM31																													
BLM32																													

Proportion matrices

Network	Boujaad	nche de Monta	Beni-Guil	D'man	Sardi	Timahdite
Moroccan	0.419	0.969	0.935	0.931	0.697	0.784
Iberian	0.226	0.000	0.000	0.000	0.091	0.081
Italian	0.290	0.000	0.032	0.069	0.152	0.081
Oriental	0.065	0.031	0.032	0.000	0.061	0.054
Total	100%	100%	100%	100%	100%	100%

Newik-Etra	Boujaad	nche de Monta	Beni-Guil	D'man	Sardi	Timahdite
Moroccan	77%	80%	68%	100%	78%	80%
Iberian	8%	11%	16%	0%	11%	8%
Italian	8%	7%	11%	0%	9%	12%
Oriental	8%	2%	5%	0%	2%	0%
Total	100%	100%	100%	100%	100%	100%

Breeds	Individuals	Network correspondance	Breeds	Individuals	Network correspondance
Garze Tibetan	GARZE TIBETAN KU681175.1	GARZ01	Gentile de Puglia	GENTILE DE PUGLIA KF228668.1	GENT01
	GARZE TIBETAN KU681176.1	GARZ02		GENTILE DE PUGLIA KF228669.1	GENT02
	GARZE TIBETAN KU681177.1	GARZ03		GENTILE DE PUGLIA KF228670.1	GENT03
	GARZE TIBETAN KU681179.1	GARZ04		GENTILE DE PUGLIA KF228671.1	GENT04
	GARZE TIBETAN KU681180.1	GARZ05		GENTILE DE PUGLIA KF228672.1	GENT05
	GARZE TIBETAN KU681181.1	GARZ06		GENTILE DE PUGLIA KF228673.1	GENT06
	GARZE TIBETAN KU681182.1	GARZ07		GENTILE DE PUGLIA KF228674.1	GENT07
	GARZE TIBETAN KU681183.1	GARZ08		GENTILE DE PUGLIA KF228675.1	GENT08
	GARZE TIBETAN KU681184.1	GARZ09		GENTILE DE PUGLIA KF228676.1	GENT09
	GARZE TIBETAN KU681185.1	GARZ10		GENTILE DE PUGLIA KF228677.1	GENT10
	GARZE TIBETAN KU681186.1	GARZ11		GENTILE DE PUGLIA KF228678.1	GENT11
	GARZE TIBETAN KU681187.1	GARZ12		GENTILE DE PUGLIA KF228679.1	GENT12
	GARZE TIBETAN KU681188.1	GARZ13		GENTILE DE PUGLIA KF228680.1	GENT13
	GARZE TIBETAN KU681189.1	GARZ14		GENTILE DE PUGLIA KF228681.1	GENT14
	GARZE TIBETAN KU681190.1	GARZ15		GENTILE DE PUGLIA KF228682.1	GENT15
	GARZE TIBETAN KU681191.1	GARZ16		GENTILE DE PUGLIA KF228683.1	GENT16
	GARZE TIBETAN KU681192.1	GARZ17		GENTILE DE PUGLIA KF228684.1	GENT17
Akkaraman	AKKARAMAN AY091497.1	AKKA01		GENTILE DE PUGLIA KF228685.1	GENT18
	AKKARAMAN JN574132.1	AKKA02		GENTILE DE PUGLIA KF228686.1	GENT19
	AKKARAMAN JN574133.1	AKKA03		GENTILE DE PUGLIA KF228687.1	GENT20
	AKKARAMAN JN574134.1	AKKA04		GENTILE DE PUGLIA KF228688.1	GENT21
	AKKARAMAN JN574135.1	AKKA05		GENTILE DE PUGLIA KF228689.1	GENT22
	AKKARAMAN JN574136.1	AKKA06		GENTILE DE PUGLIA KF228690.1	GENT23
	AKKARAMAN JN574137.1	AKKA07		GENTILE DE PUGLIA KF228691.1	GENT24
	AKKARAMAN JN574138.1	AKKA08		GENTILE DE PUGLIA KF228692.1	GENT25
	AKKARAMAN JN574139.1	AKKA09		GENTILE DE PUGLIA KF228693.1	GENT26
	AKKARAMAN JN574140.1	AKKA10		GENTILE DE PUGLIA KF228694.1	GENT27
	AKKARAMAN JN574141.1	AKKA11		GENTILE DE PUGLIA KF302448.1	GENT28
	AKKARAMAN JN574142.1	AKKA12		GENTILE DE PUGLIA KF302455.1	GENT29
	AKKARAMAN JN574143.1	AKKA13		GENTILE DE PUGLIA KF302457.1	GENT30
	AKKARAMAN JN574144.1	AKKA14	German Merino	GERMAN MERINO JN573922.1	GERM01
	AKKARAMAN JN574147.1	AKKA17		GERMAN MERINO JN573923.1	GERM02
	AKKARAMAN KF677049.1	AKKA18		GERMAN MERINO JN573924.1	GERM03
Churra Algarvia	CHURRA ALGARVIA DQ491580.1	ALGA01		GERMAN MERINO JN573925.1	GERM04
	CHURRA ALGARVIA DQ491589.1	ALGA02		GERMAN MERINO JN573926.1	GERM05
	CHURRA ALGARVIA DQ491591.1	ALGA03		GERMAN MERINO JN573927.1	GERM06
	CHURRA ALGARVIA DQ491646.1	ALGA04		GERMAN MERINO JN573928.1	GERM07
	CHURRA ALGARVIA DQ491649.1	ALGA05		GERMAN MERINO JN573929.1	GERM08
	CHURRA ALGARVIA DQ491652.1	ALGA06		GERMAN MERINO JN573930.1	GERM09
	CHURRA ALGARVIA DQ491654.1	ALGA07		GERMAN MERINO JN573931.1	GERM10
	CHURRA ALGARVIA DQ491656.1	ALGA08		GERMAN MERINO JN573932.1	GERM11
	CHURRA ALGARVIA DQ491658.1	ALGA09		GERMAN MERINO JN573933.1	GERM12
	CHURRA ALGARVIA DQ491664.1	ALGA10		GERMAN MERINO JN573934.1	GERM13
	CHURRA ALGARVIA DQ491678.1	ALGA11		GERMAN MERINO JN573935.1	GERM14
	CHURRA ALGARVIA DQ491687.1	ALGA12		GERMAN MERINO JN573936.1	GERM15
	CHURRA ALGARVIA DQ491699.1	ALGA13		GERMAN MERINO JN573937.1	GERM16
	CHURRA ALGARVIA DQ491706.1	ALGA14		GERMAN MERINO JN573938.1	GERM17
	CHURRA ALGARVIA DQ491708.1	ALGA15		GERMAN MERINO JN573939.1	GERM18
	CHURRA ALGARVIA DQ491712.1	ALGA16		GERMAN MERINO JN573940.1	GERM19
	CHURRA ALGARVIA DQ491713.1	ALGA17		GERMAN MERINO JN573941.1	GERM20
	CHURRA ALGARVIA DQ491714.1	ALGA18		GERMAN MERINO JN573942.1	GERM21
	CHURRA ALGARVIA DQ491719.1	ALGA19		GERMAN MERINO JN573943.1	GERM22
	CHURRA ALGARVIA DQ491720.1	ALGA20		GERMAN MERINO JN573944.1	GERM23
	CHURRA ALGARVIA DQ491722.1	ALGA21		GERMAN MERINO JN573945.1	GERM24
	CHURRA ALGARVIA DQ491723.1	ALGA22		GERMAN MERINO JN573946.1	GERM25
	CHURRA ALGARVIA DQ491724.1	ALGA23		GERMAN MERINO JN573947.1	GERM26
	CHURRA ALGARVIA DQ491725.1	ALGA24		GERMAN MERINO JN573948.1	GERM27
	CHURRA ALGARVIA DQ491728.1	ALGA25		GERMAN MERINO JN574174.1	GERM28
	CHURRA ALGARVIA DQ491730.1	ALGA26		GERMAN MERINO JN574209.1	GERM29
	CHURRA ALGARVIA DQ491732.1	ALGA27	Herik	HERIK KF677119.1	HERI01
	CHURRA ALGARVIA DQ491733.1	ALGA28		HERIK KF677120.1	HERI02
	CHURRA ALGARVIA DQ491735.1	ALGA29		HERIK KF677121.1	HERI03
	CHURRA ALGARVIA DQ491736.1	ALGA30		HERIK KF677122.1	HERI04
Appenninica	APPENNINICA KF228617.1	APPE01		HERIK KF677123.1	HERI05
	APPENNINICA KF228618.1	APPE02		HERIK KF677124.1	HERI06
	APPENNINICA KF228612.1	APPE03		HERIK KF677125.1	HERI07
	APPENNINICA KF228613.1	APPE04		HERIK KF677126.1	HERI08
	APPENNINICA KF228614.1	APPE05		HERIK KF677127.1	HERI09
	APPENNINICA KF228615.1	APPE06		HERIK KF677128.1	HERI10
	APPENNINICA KF228616.1	APPE07		HERIK KF677129.1	HERI11
	APPENNINICA KF228619.1	APPE08		HERIK KF677130.1	HERI12
	APPENNINICA KF228620.1	APPE09		HERIK KF677131.1	HERI13
	APPENNINICA KF228621.1	APPE10		HERIK KF677132.1	HERI14
	APPENNINICA KF228622.1	APPE11		HERIK KF677133.1	HERI15
	APPENNINICA KF228623.1	APPE12	Karakas	KARAKAS DQ852282.1	KARK1
	APPENNINICA KF228624.1	APPE13		KARAKAS DQ852283.1	KARK2
	APPENNINICA KF228625.1	APPE14		KARAKAS DQ852284.1	KARK3
	APPENNINICA KF228626.1	APPE15		KARAKAS DQ852285.1	KARK4
	APPENNINICA KF228627.1	APPE16		KARAKAS DQ852286.1	KARK5
	APPENNINICA KF228628.1	APPE17		KARAKAS DQ852287.1	KARK6
	APPENNINICA KF228629.1	APPE18		KARAKAS DQ852288.1 D	KARK7
	APPENNINICA KF228630.1	APPE19		KARAKAS DQ852289.1	KARK8
	APPENNINICA KF228631.1	APPE20		KARAKAS HM236176.1	KARK9
	APPENNINICA KF228632.1	APPE21		KARAKAS HM236177.1	KARK10
	APPENNINICA KF228633.1	APPE22		KARAKAS HM236178.1	KARK11
	APPENNINICA KF228634.1	APPE23		KARAYAKA JN574154.1	KARY01
	APPENNINICA KF228636.1	APPE24		KARAYAKA JN574155.1	KARY02
	APPENNINICA KF228637.1	APPE25		KARAYAKA JN574156.1	KARY03
	APPENNINICA KF228638.1	APPE26		KARAYAKA JN574157.1	KARY04
	APPENNINICA KF228639.1	APPE27		KARAYAKA JN574158.1	KARY05
	APPENNINICA KF302450.1	APPE28		KARAYAKA JN574159.1	KARY06
	APPENNINICA KF302451.1	APPE29		KARAYAKA JN574265.1	KARY07

Awassi	Awassi HM236182.1 E	Aw_H01
	Awassi JX235859.1	Aw_J02
	Awassi JX235861.1	Aw_J03
Churra Badana	CHURRA BADANA DQ491625.1	BADD01
	CHURRA BADANA DQ491626.1	BADD02
	CHURRA BADANA DQ491629.1	BADD03
	CHURRA BADANA DQ491647.1	BADD04
	CHURRA BADANA DQ491653.1	BADD05
	CHURRA BADANA DQ491660.1	BADD06
	CHURRA BADANA DQ491661.1	BADD07
	CHURRA BADANA DQ491663.1	BADD08
	CHURRA BADANA DQ491671.1	BADD09
	CHURRA BADANA DQ491683.1	BADD10
	CHURRA BADANA DQ491684.1	BADD11
	CHURRA BADANA DQ491685.1	BADD12
	CHURRA BADANA DQ491688.1	BADD13
	CHURRA BADANA DQ491690.1	BADD14
	CHURRA BADANA DQ491692.1	BADD15
	CHURRA BADANA DQ491704.1	BADD16
	CHURRA BADANA DQ491721.1	BADD17
	CHURRA BADANA DQ491726.1	BADD18
Baruwal	BARUWAL KR610850.1	BAR_01
	BARUWAL KR610851.1	BAR_02
	BARUWAL KR610852.1	BAR_03
	BARUWAL KR610853.1	BAR_04
	BARUWAL KR610854.1	BAR_05
	BARUWAL KR610855.1	BAR_06
	BARUWAL KR610856.1	BAR_07
	BARUWAL KR610857.1	BAR_08
	BARUWAL KR610858.1	BAR_09
	BARUWAL KR610859.1	BAR_10
	BARUWAL KR610860.1	BAR_11
	BARUWAL KR610861.1	BAR_12
	BARUWAL KR610862.1	BAR_13
	BARUWAL KR610863.1	BAR_14
	BARUWAL KR610864.1	BAR_15
	BARUWAL KR610865.1	BAR_16
	BARUWAL KR610866.1	BAR_17
	BARUWAL KR610867.1	BAR_18
	BARUWAL KR610868.1	BAR_19
	BARUWAL KR610869.1	BAR_20
	BARUWAL KR610870.1	BAR_21
	BARUWAL KR610871.1	BAR_22
	BARUWAL KR610872.1	BAR_23
	BARUWAL KR610873.1	BAR_24
	BARUWAL KR610874.1	BAR_25
	BARUWAL KR610875.1	BAR_26
	BARUWAL KR610876.1	BAR_27
Boujaad	Boujaad MN229085	BJD01
	Boujaad MN229086	BJD02
	Boujaad MN229087	BJD03
	Boujaad MN229088	BJD04
	Boujaad MN229089	BJD05
	Boujaad MN229090	BJD06
	Boujaad MN229091	BJD07
	Boujaad MN229092	BJD08
	Boujaad MN229093	BJD09
	Boujaad MN229094	BJD10
	Boujaad MN229095	BJD11
	Boujaad MN229096	BJD12
	Boujaad MN229097	BJD13
	Boujaad MN229098	BJD14
	Boujaad MN229099	BJD15
	Boujaad MN229100	BJD16
	Boujaad MN229101	BJD17
	Boujaad MN229102	BJD18
	Boujaad MN229103	BJD19
	Boujaad MN229104	BJD20
	Boujaad MN229105	BJD21
	Boujaad MN229106	BJD22
	Boujaad MN229107	BJD23
	Boujaad MN229108	BJD24
	Boujaad MN229109	BJD25
	Boujaad MN229110	BJD26
	Boujaad MN229111	BJD27
	Boujaad MN229112	BJD28
	Boujaad MN229113	BJD29
	Boujaad MN229114	BJD30
	Boujaad MN229115	BJD31
	LATXA BLONDE FACE KT879022.1	BLDF01
	LATXA BLONDE FACE KT879030.1	BLDF02
	LATXA BLONDE FACE KT879036.1	BLDF03
	LATXA BLONDE FACE KT879040.1	BLDF04
	LATXA BLONDE FACE KT879042.1	BLDF05
	LATXA BLONDE FACE KT879043.1	BLDF06
	LATXA BLONDE FACE KT879046.1	BLDF07
	LATXA BLONDE FACE KT879083.1	BLDF08
	LATXA BLONDE FACE KT879085.1	BLDF09
	LATXA BLONDE FACE KT879095.1	BLDF10
	LATXA BLONDE FACE KT879101.1	BLDF11
	LATXA BLONDE FACE KT879118.1	BLDF12
	LATXA BLONDE FACE KT879126.1	BLDF13
	LATXA BLONDE FACE KT879134.1	BLDF14

Karayaka	KARAYAKA JN574266.1	KARY08
	KARAYAKA JN574267.1	KARY09
	KARAYAKA JN574268.1	KARY10
	KARAYAKA JN574269.1	KARY11
	KARAYAKA KF677024.1	KARY12
	KARAYAKA KF677025.1	KARY13
	KARAYAKA KF677026.1	KARY14
	KARAYAKA KF677028.1	KARY15
	KARAYAKA KF677029.1	KARY16
	KARAYAKA KF677030.1	KARY17
	KARAYAKA KF677031.1	KARY18
	KARAYAKA KF677032.1	KARY19
	KARAYAKA KF677033.1	KARY20
	KARAYAKA KF677034.1	KARY21
	KARAYAKA KF677035.1	KARY22
	KARAYAKA KF677036.1	KARY23
	KARAYAKA KF677037.1	KARY24
	KARAYAKA KF677038.1	KARY25
	KARAYAKA MG209458.1	KARY26
	KARAYAKA MG209461.1	KARY27
	KARAYAKA MG209462.1	KARY28
	KARAYAKA MG209465.1	KARY29
	KARAYAKA MG209466.1	KARY30
Lacaune of Italy	LACAUNE OF ITALY KF228745.1	LACA01
	LACAUNE OF ITALY KF228746.1	LACA02
	LACAUNE OF ITALY KF228763.1	LACA03
	LACAUNE OF ITALY KF228766.1	LACA04
	LACAUNE OF ITALY KF228769.1	LACA05
	LACAUNE OF ITALY KF228770.1	LACA06
	LACAUNE OF ITALY KF228771.1	LACA07
	LACAUNE OF ITALY KF228772.1	LACA08
	LACAUNE OF ITALY KF228773.1	LACA09
	LACAUNE OF ITALY KF228774.1	LACA10
	LACAUNE OF ITALY KF228775.1	LACA11
	LACAUNE OF ITALY KF228776.1	LACA12
	LACAUNE OF ITALY KF228777.1	LACA13
	LACAUNE OF ITALY KF228779.1	LACA14
	LACAUNE OF ITALY KF228785.1	LACA15
	LACAUNE OF ITALY KF228786.1	LACA16
	LACAUNE OF ITALY KF228787.1	LACA17
	LACAUNE OF ITALY KF228788.1	LACA18
	LACAUNE OF ITALY KF228789.1	LACA19
	LACAUNE OF ITALY KF228790.1	LACA20
	LACAUNE OF ITALY KF228791.1	LACA21
	LACAUNE OF ITALY KF228792.1	LACA22
	LACAUNE OF ITALY KF228793.1	LACA23
	LACAUNE OF ITALY KF228794.1	LACA24
	LACAUNE OF ITALY KF228795.1	LACA25
	LACAUNE OF ITALY KF228796.1	LACA26
	LACAUNE OF ITALY KF228797.1	LACA27
	LACAUNE OF ITALY KF302447.1	LACA28
	LACAUNE OF ITALY KF302453.1	LACA29
	LACAUNE OF ITALY KF302460.1	LACA30
Merinizzata Italiana	MERINIZZATA ITALIANA KF228640.1	MRNZ01
	MERINIZZATA ITALIANA KF228641.1	MRNZ02
	MERINIZZATA ITALIANA KF228642.1	MRNZ03
	MERINIZZATA ITALIANA KF228643.1	MRNZ04
	MERINIZZATA ITALIANA KF228644.1	MRNZ05
	MERINIZZATA ITALIANA KF228645.1	MRNZ06
	MERINIZZATA ITALIANA KF228646.1	MRNZ07
	MERINIZZATA ITALIANA KF228647.1	MRNZ08
	MERINIZZATA ITALIANA KF228648.1	MRNZ09
	MERINIZZATA ITALIANA KF228649.1	MRNZ10
	MERINIZZATA ITALIANA KF228650.1	MRNZ11
	MERINIZZATA ITALIANA KF228651.1	MRNZ12
	MERINIZZATA ITALIANA KF228652.1	MRNZ13
	MERINIZZATA ITALIANA KF228653.1	MRNZ14
	MERINIZZATA ITALIANA KF228654.1	MRNZ15
	MERINIZZATA ITALIANA KF228655.1	MRNZ16
	MERINIZZATA ITALIANA KF228656.1	MRNZ17
	MERINIZZATA ITALIANA KF228657.1	MRNZ18
	MERINIZZATA ITALIANA KF228658.1	MRNZ19
	MERINIZZATA ITALIANA KF228659.1	MRNZ20
	MERINIZZATA ITALIANA KF228660.1	MRNZ21
	MERINIZZATA ITALIANA KF228661.1	MRNZ22
	MERINIZZATA ITALIANA KF228662.1	MRNZ23
	MERINIZZATA ITALIANA KF228663.1	MRNZ24
	MERINIZZATA ITALIANA KF228664.1	MRNZ25
	MERINIZZATA ITALIANA KF228665.1	MRNZ26
	MERINIZZATA ITALIANA KF228666.1	MRNZ27
	MERINIZZATA ITALIANA KF228667.1	MRNZ28
	MERINIZZATA ITALIANA KF302446.1	MRNZ29
	MERINIZZATA ITALIANA KF302459.1	MRNZ30
	MORKARAMAN HM236179.1 C	MORK01
	MORKARAMAN HM236181.1 D	MORK02
	MORKARAMAN JN574160.1	MORK03
	MORKARAMAN JN574161.1	MORK04
	MORKARAMAN JN574162.1	MORK05
	MORKARAMAN JN574163.1	MORK06
	MORKARAMAN JN574164.1	MORK07
	MORKARAMAN JN574165.1	MORK08
	MORKARAMAN JN574166.1	MORK09
	MORKARAMAN JN574167.1	MORK10

Latxa Blonde Face	LATXA BLONDE FACE KT879138.1	BLDF15
	LATXA BLONDE FACE KT879141.1	BLDF16
	LATXA BLONDE FACE KT879147.1	BLDF17
	LATXA BLONDE FACE KT879148.1	BLDF18
	LATXA BLONDE FACE KT879149.1	BLDF19
	LATXA BLONDE FACE KT879150.1	BLDF20
	LATXA BLONDE FACE KT879151.1	BLDF21
	LATXA BLONDE FACE KT879152.1	BLDF22
	LATXA BLONDE FACE KT879153.1	BLDF23
	LATXA BLONDE FACE KT879155.1	BLDF24
	LATXA BLONDE FACE KT879169.1	BLDF25
	LATXA BLONDE FACE KT879170.1	BLDF26
	LATXA BLONDE FACE KT879171.1	BLDF27
	LATXA BLONDE FACE KT879172.1	BLDF28
	LATXA BLONDE FACE KT879173.1	BLDF29
	LATXA BLONDE FACE KT879184.1	BLDF30
	LATXA BLONDE FACE KT879185.1	BLDF31
Latxa Black Face	LATXA BLACK FACE KT879022.1	BLKF01
	LATXA BLACK FACE KT879038.1	BLKF02
	LATXA BLACK FACE KT879073.1	BLKF03
	LATXA BLACK FACE KT879099.1	BLKF04
	LATXA BLACK FACE KT879134.1	BLKF05
	LATXA BLACK FACE KT879136.1	BLKF06
	LATXA BLACK FACE KT879146.1	BLKF07
	LATXA BLACK FACE KT879154.1	BLKF08
	LATXA BLACK FACE KT879156.1	BLKF09
	LATXA BLACK FACE KT879157.1	BLKF10
	LATXA BLACK FACE KT879158.1	BLKF11
	LATXA BLACK FACE KT879159.1	BLKF12
	LATXA BLACK FACE KT879160.1	BLKF13
	LATXA BLACK FACE KT879161.1	BLKF14
	LATXA BLACK FACE KT879162.1	BLKF15
	LATXA BLACK FACE KT879163.1	BLKF16
	LATXA BLACK FACE KT879164.1	BLKF17
	LATXA BLACK FACE KT879165.1	BLKF18
	LATXA BLACK FACE KT879166.1	BLKF19
	LATXA BLACK FACE KT879167.1	BLKF20
	LATXA BLACK FACE KT879168.1	BLKF21
	LATXA BLACK FACE KT879174.1	BLKF22
	LATXA BLACK FACE KT879175.1	BLKF23
	LATXA BLACK FACE KT879176.1	BLKF24
	LATXA BLACK FACE KT879178.1	BLKF25
	LATXA BLACK FACE KT879179.1	BLKF26
	LATXA BLACK FACE KT879180.1	BLKF27
	LATXA BLACK FACE KT879181.1	BLKF28
	LATXA BLACK FACE KT879182.1	BLKF29
	LATXA BLACK FACE KT879183.1	BLKF30
	LATXA BLACK FACE KT879186.1	BLKF31
Blanche de Montagne	BLANCHE DE MONTAGNE MN229116	BLM01
	BLANCHE DE MONTAGNE MN229117	BLM02
	BLANCHE DE MONTAGNE MN229118	BLM03
	BLANCHE DE MONTAGNE MN229119	BLM04
	BLANCHE DE MONTAGNE MN229120	BLM05
	BLANCHE DE MONTAGNE MN229121	BLM06
	BLANCHE DE MONTAGNE MN229122	BLM07
	BLANCHE DE MONTAGNE MN229123	BLM08
	BLANCHE DE MONTAGNE MN229124	BLM09
	BLANCHE DE MONTAGNE MN229125	BLM10
	BLANCHE DE MONTAGNE MN229126	BLM11
	BLANCHE DE MONTAGNE MN229127	BLM12
	BLANCHE DE MONTAGNE MN229128	BLM13
	BLANCHE DE MONTAGNE MN229129	BLM14
	BLANCHE DE MONTAGNE MN229130	BLM15
	BLANCHE DE MONTAGNE MN229131	BLM16
	BLANCHE DE MONTAGNE MN229132	BLM17
	BLANCHE DE MONTAGNE MN229133	BLM18
	BLANCHE DE MONTAGNE MN229134	BLM19
	BLANCHE DE MONTAGNE MN229135	BLM20
	BLANCHE DE MONTAGNE MN229136	BLM21
	BLANCHE DE MONTAGNE MN229137	BLM22
	BLANCHE DE MONTAGNE MN229138	BLM23
	BLANCHE DE MONTAGNE MN229139	BLM24
	BLANCHE DE MONTAGNE MN229140	BLM25
	BLANCHE DE MONTAGNE MN229141	BLM26
	BLANCHE DE MONTAGNE MN229142	BLM27
	BLANCHE DE MONTAGNE MN229143	BLM28
	BLANCHE DE MONTAGNE MN229144	BLM29
	BLANCHE DE MONTAGNE MN229145	BLM30
	BLANCHE DE MONTAGNE MN229146	BLM31
	BLANCHE DE MONTAGNE MN229147	BLM32
	BENI-GUIL MN229148	BNG01
	BENI-GUIL MN229149	BNG02
	BENI-GUIL MN229150	BNG03
	BENI-GUIL MN229151	BNG04
	BENI-GUIL MN229152	BNG05
	BENI-GUIL MN229153	BNG06
	BENI-GUIL MN229154	BNG07
	BENI-GUIL MN229155	BNG08
	BENI-GUIL MN229156	BNG09
	BENI-GUIL MN229157	BNG10
	BENI-GUIL MN229158	BNG11
	BENI-GUIL MN229159	BNG12
	BENI-GUIL MN229160	BNG13

Morkaraman	MORKARAMAN KF677238.1	MORK11
	MORKARAMAN KF677239.1	MORK12
	MORKARAMAN KF677240.1	MORK13
	MORKARAMAN KF677241.1	MORK14
	MORKARAMAN KF677242.1	MORK15
	MORKARAMAN KF677243.1	MORK16
	MORKARAMAN KF677244.1	MORK17
	MORKARAMAN KF677245.1	MORK18
	MORKARAMAN KF677246.1	MORK19
	MORKARAMAN KF677247.1	MORK20
	MORKARAMAN KF677248.1	MORK21
	MORKARAMAN KF677249.1	MORK22
	MORKARAMAN KF677250.1	MORK23
	MORKARAMAN KF677251.1	MORK24
	MORKARAMAN KF677252.1	MORK25
	MORKARAMAN KF677253.1	MORK26
	MORKARAMAN KF677254.1	MORK27
	MORKARAMAN KF677255.1	MORK28
	MORKARAMAN KF677256.1	MORK29
	MORKARAMAN KF677257.1	MORK30
	MORKARAMAN KF677258.1	MORK31
	MORKARAMAN KF677259.1	MORK32
	MORKARAMAN KF677260.1	MORK33
	MORKARAMAN KF677261.1	MORK34
	MORKARAMAN KF677262.1	MORK35
	MORKARAMAN KF677263.1	MORK36
Norduz	NORDUZ KF677193.1	NORD01
	NORDUZ KF677199.1	NORD02
	NORDUZ KF677200.1	NORD03
	NORDUZ KF677201.1	NORD04
	NORDUZ KF677202.1	NORD05
	NORDUZ KF677203.1	NORD06
	NORDUZ KF677204.1	NORD07
	NORDUZ KF677205.1	NORD08
	NORDUZ KF677206.1	NORD09
	NORDUZ KF677207.1	NORD10
	NORDUZ KF677208.1	NORD11
	NORDUZ KF677209.1	NORD12
	NORDUZ KF677210.1	NORD13
Saloia	SALOIA DQ491579.1	SALO01
	SALOIA DQ491597.1	SALO02
	SALOIA DQ491599.1	SALO03
	SALOIA DQ491604.1	SALO04
	SALOIA DQ491608.1	SALO05
	SALOIA DQ491610.1	SALO06
	SALOIA DQ491615.1	SALO07
	SALOIA DQ491617.1	SALO08
	SALOIA DQ491622.1	SALO09
	SALOIA DQ491623.1	SALO10
	SALOIA DQ491627.1	SALO11
	SALOIA DQ491628.1	SALO12
	SALOIA DQ491630.1	SALO13
	SALOIA DQ491632.1	SALO14
	SALOIA DQ491634.1	SALO15
	SALOIA DQ491637.1	SALO16
	SALOIA DQ491644.1	SALO17
	SALOIA DQ491648.1	SALO18
	SALOIA DQ491669.1	SALO19
	SALOIA DQ491670.1	SALO20
	SALOIA DQ491676.1	SALO21
	SALOIA DQ491677.1	SALO22
	SALOIA DQ491679.1	SALO23
	SALOIA DQ491691.1	SALO24
	SALOIA DQ491693.1	SALO25
	SALOIA DQ491694.1	SALO26
	SALOIA DQ491697.1	SALO27
	SALOIA DQ491703.1	SALO28
	SALOIA DQ491710.1	SALO29
	SALOIA DQ491715.1	SALO30
Sarda	SARDA KF228824.1	SRDA01
	SARDA KF228825.1	SRDA02
	SARDA KF228826.1	SRDA03
	SARDA KF228827.1	SRDA04
	SARDA KF228828.1	SRDA05
	SARDA KF228829.1	SRDA06
	SARDA KF228830.1	SRDA07
	SARDA KF228831.1	SRDA08
	SARDA KF228832.1	SRDA09
	SARDA KF228833.1	SRDA10
	SARDA KF228834.1	SRDA11
	SARDA KF228835.1	SRDA12
	SARDA KF228836.1	SRDA13
	SARDA KF228837.1	SRDA14
	SARDA KF228838.1	SRDA15
	SARDA KF228839.1	SRDA16
	SARDA KF228840.1	SRDA17
	SARDA KF228841.1	SRDA18
	SARDA KF228842.1	SRDA19
	SARDA KF228843.1	SRDA20
	SARDA KF228844.1	SRDA21
	SARDA KF228845.1	SRDA22
	SARDA KF228846.1	SRDA23
	SARDA KF228847.1	SRDA24

Beni-Guil	BENI-GUIL MN229161	BNG14
	BENI-GUIL MN229162	BNG15
	BENI-GUIL MN229163	BNG16
	BENI-GUIL MN229164	BNG17
	BENI-GUIL MN229165	BNG18
	BENI-GUIL MN229166	BNG19
	BENI-GUIL MN229167	BNG20
	BENI-GUIL MN229168	BNG21
	BENI-GUIL MN229169	BNG22
	BENI-GUIL MN229170	BNG23
	BENI-GUIL MN229171	BNG24
	BENI-GUIL MN229172	BNG25
	BENI-GUIL MN229173	BNG26
	BENI-GUIL MN229174	BNG27
	BENI-GUIL MN229175	BNG28
	BENI-GUIL MN229176	BNG29
	BENI-GUIL MN229177	BNG30
	BENI-GUIL MN229178	BNG31
hurra de Terra Quent	CHURRA DE TERRA QUENTE DQ491706.1	CTQ01
	CHURRA DE TERRA QUENTE DQ491581.1	CTQ02
	CHURRA DE TERRA QUENTE DQ491583.1	CTQ03
	CHURRA DE TERRA QUENTE DQ491584.1	CTQ04
	CHURRA DE TERRA QUENTE DQ491588.1	CTQ05
	CHURRA DE TERRA QUENTE DQ491596.1	CTQ06
	CHURRA DE TERRA QUENTE DQ491609.1	CTQ07
	CHURRA DE TERRA QUENTE DQ491614.1	CTQ08
	CHURRA DE TERRA QUENTE DQ491620.1	CTQ09
	CHURRA DE TERRA QUENTE DQ491624.1	CTQ10
	CHURRA DE TERRA QUENTE DQ491631.1	CTQ11
	CHURRA DE TERRA QUENTE DQ491636.1	CTQ12
	CHURRA DE TERRA QUENTE DQ491638.1	CTQ13
	CHURRA DE TERRA QUENTE DQ491640.1	CTQ14
	CHURRA DE TERRA QUENTE DQ491645.1	CTQ15
	CHURRA DE TERRA QUENTE DQ491650.1	CTQ16
	CHURRA DE TERRA QUENTE DQ491666.1	CTQ17
	CHURRA DE TERRA QUENTE DQ491672.1	CTQ18
	CHURRA DE TERRA QUENTE DQ491673.1	CTQ19
	CHURRA DE TERRA QUENTE DQ491674.1	CTQ20
	CHURRA DE TERRA QUENTE DQ491696.1	CTQ21
	CHURRA DE TERRA QUENTE DQ491701.1	CTQ22
	CHURRA DE TERRA QUENTE DQ491705.1	CTQ23
	CHURRA DE TERRA QUENTE DQ491707.1	CTQ24
	CHURRA DE TERRA QUENTE DQ491716.1	CTQ25
Campaniça	CAMPANICA DQ491595.1	CAMP01
	CAMPANICA DQ491602.1	CAMP02
	CAMPANICA DQ491611.1	CAMP03
	CAMPANICA DQ491612.1	CAMP04
	CAMPANICA DQ491618.1	CAMP05
	CAMPANICA DQ491619.1	CAMP06
	CAMPANICA DQ491621.1	CAMP07
	CAMPANICA DQ491655.1	CAMP08
	CAMPANICA DQ491657.1	CAMP09
	CAMPANICA DQ491659.1	CAMP10
	CAMPANICA DQ491662.1	CAMP11
	CAMPANICA DQ491667.1	CAMP12
	CAMPANICA DQ491668.1	CAMP13
	CAMPANICA DQ491680.1	CAMP14
	CAMPANICA DQ491681.1	CAMP15
	CAMPANICA DQ491695.1	CAMP16
	CAMPANICA DQ491698.1	CAMP17
	CAMPANICA DQ491709.1	CAMP18
	CAMPANICA DQ491729.1	CAMP19
	CAMPANICA DQ491731.1	CAMP20
Comisana	COMISANA KF228724.1	COMI01
	COMISANA KF302445.1	COMI02
	COMISANA KF302452.1	COMI03
	COMISANA KF302462.1	COMI04
	COMISANA KF228695.1	COMI05
	COMISANA KF228696.1	COMI06
	COMISANA KF228698.1	COMI07
	COMISANA KF228699.1	COMI08
	COMISANA KF228700.1	COMI09
	COMISANA KF228703.1	COMI10
	COMISANA KF228704.1	COMI11
	COMISANA KF228705.1	COMI12
	COMISANA KF228706.1	COMI13
	COMISANA KF228709.1	COMI14
	COMISANA KF228710.1	COMI15
	COMISANA KF228711.1	COMI16
	COMISANA KF228713.1	COMI17
	COMISANA KF228714.1	COMI18
	COMISANA KF228715.1	COMI19
	COMISANA KF228717.1	COMI20
	COMISANA KF228718.1	COMI21
	COMISANA KF228719.1	COMI22
	COMISANA KF228720.1	COMI23
	COMISANA KF228721.1	COMI24
	COMISANA KF228722.1	COMI25
	COMISANA KF228723.1	COMI26
	COMISANA KF228725.1	COMI27
	COMISANA KF228726.1	COMI28
	COMISANA KF228727.1	COMI29
	COMISANA KF228730.1	COMI30

	SARDA KF228848.1	SRDA25
	SARDA KF228849.1	SRDA26
	SARDA KF228850.1	SRDA27
	SARDA KF228851.1	SRDA28
	SARDA KF228852.1	SRDA29
	SARDA KF228853.1	SRDA30
Sadri	SARDI MN229208	SRDI01
	SARDI MN229209	SRDI02
	SARDI MN229210	SRDI03
	SARDI MN229211	SRDI04
	SARDI MN229212	SRDI05
	SARDI MN229213	SRDI06
	SARDI MN229214	SRDI07
	SARDI MN229215	SRDI08
	SARDI MN229216	SRDI09
	SARDI MN229217	SRDI10
	SARDI MN229218	SRDI11
	SARDI MN229219	SRDI12
	SARDI MN229220	SRDI13
	SARDI MN229221	SRDI14
	SARDI MN229222	SRDI15
	SARDI MN229223	SRDI16
	SARDI MN229224	SRDI17
	SARDI MN229225	SRDI18
	SARDI MN229226	SRDI19
	SARDI MN229227	SRDI20
	SARDI MN229228	SRDI21
	SARDI MN229229	SRDI22
	SARDI MN229230	SRDI23
	SARDI MN229231	SRDI24
	SARDI MN229232	SRDI25
	SARDI MN229233	SRDI26
	SARDI MN229234	SRDI27
	SARDI MN229235	SRDI28
	SARDI MN229236	SRDI29
	SARDI MN229237	SRDI30
	SARDI MN229238	SRDI31
	SARDI MN229239	SRDI32
	SARDI MN229240	SRDI33
Sopravissana	SOPRAVISSANA KF228586.1	SOPR01
	SOPRAVISSANA KF228587.1	SOPR02
	SOPRAVISSANA KF228588.1	SOPR03
	SOPRAVISSANA KF228589.1	SOPR04
	SOPRAVISSANA KF228590.1	SOPR05
	SOPRAVISSANA KF228591.1	SOPR06
	SOPRAVISSANA KF228592.1	SOPR07
	SOPRAVISSANA KF228593.1	SOPR08
	SOPRAVISSANA KF228594.1	SOPR09
	SOPRAVISSANA KF228595.1	SOPR10
	SOPRAVISSANA KF228596.1	SOPR11
	SOPRAVISSANA KF228597.1	SOPR12
	SOPRAVISSANA KF228598.1	SOPR13
	SOPRAVISSANA KF228599.1	SOPR14
	SOPRAVISSANA KF228600.1	SOPR15
	SOPRAVISSANA KF228601.1	SOPR16
	SOPRAVISSANA KF228602.1	SOPR17
	SOPRAVISSANA KF228603.1	SOPR18
	SOPRAVISSANA KF228604.1	SOPR19
	SOPRAVISSANA KF228605.1	SOPR20
	SOPRAVISSANA KF228606.1	SOPR21
	SOPRAVISSANA KF228607.1	SOPR22
	SOPRAVISSANA KF228608.1	SOPR23
	SOPRAVISSANA KF228609.1	SOPR24
	SOPRAVISSANA KF228610.1	SOPR25
	SOPRAVISSANA KF228611.1	SOPR26
	SOPRAVISSANA KF302449.1	SOPR27
	SOPRAVISSANA KF302454.1	SOPR28
	SOPRAVISSANA KF302456.1	SOPR29
	SOPRAVISSANA KF302458.1	SOPR30
Spanish Merino	SPANISH MERINI KF228798.1	SPAN01
	SPANISH MERINI KF228799.1	SPAN02
	SPANISH MERINI KF228800.1	SPAN03
	SPANISH MERINI KF228801.1	SPAN04
	SPANISH MERINI KF228802.1	SPAN05
	SPANISH MERINI KF228803.1	SPAN06
	SPANISH MERINI KF228804.1	SPAN07
	SPANISH MERINI KF228805.1	SPAN08
	SPANISH MERINI KF228806.1	SPAN09
	SPANISH MERINI KF228807.1	SPAN10
	SPANISH MERINI KF228808.1	SPAN11
	SPANISH MERINI KF228809.1	SPAN12
	SPANISH MERINI KF228810.1	SPAN13
	SPANISH MERINI KF228811.1	SPAN14
	SPANISH MERINI KF228812.1	SPAN15
	SPANISH MERINI KF228813.1	SPAN16
	SPANISH MERINI KF228814.1	SPAN17
	SPANISH MERINI KF228815.1	SPAN18
	SPANISH MERINI KF228816.1	SPAN19
	SPANISH MERINI KF228817.1	SPAN20
	SPANISH MERINI KF228818.1	SPAN21
	SPANISH MERINI KF228819.1	SPAN22
	SPANISH MERINI KF228820.1	SPAN23
	SPANISH MERINI KF228821.1	SPAN24

Daglic	DAGLIC KF677087.1	DAGL01
	DAGLIC JN574148.1	DAGL02
	DAGLIC JN574149.1	DAGL03
	DAGLIC JN574150.1	DAGL04
	DAGLIC JN574151.1	DAGL05
	DAGLIC JN574152.1	DAGL06
	DAGLIC JN574153.1	DAGL07
	DAGLIC JN574262.1	DAGL08
	DAGLIC JN574263.1	DAGL09
	DAGLIC JN574264.1	DAGL10
	DAGLIC JN574285.1	DAGL11
	DAGLIC JN574286.1	DAGL13
	DAGLIC KF677071.1	DAGL14
	DAGLIC KF677072.1	DAGL15
	DAGLIC KF677073.1	DAGL16
	DAGLIC KF677074.1	DAGL17
	DAGLIC KF677075.1	DAGL18
	DAGLIC KF677076.1	DAGL19
	DAGLIC KF677077.1	DAGL20
	DAGLIC KF677078.1	DAGL21
	DAGLIC KF677079.1	DAGL22
	DAGLIC KF677080.1	DAGL23
	DAGLIC KF677081.1	DAGL24
	DAGLIC KF677082.1	DAGL25
	DAGLIC KF677083.1	DAGL26
	DAGLIC KF677084.1	DAGL27
	DAGLIC KF677085.1	DAGL28
	DAGLIC KF677086.1	DAGL29
	DAGLIC KF677088.1	DAGL30
	DAGLIC KF677089.1	DAGL31
	DAGLIC KF677090.1	DAGL32
	DAGLIC KF677091.1	DAGL33
	DAGLIC KF677092.1	DAGL34
D'man	DMAN MN229179	DMAN01
	DMAN MN229180	DMAN02
	DMAN MN229181	DMAN03
	DMAN MN229182	DMAN04
	DMAN MN229183	DMAN05
	DMAN MN229184	DMAN06
	DMAN MN229185	DMAN07
	DMAN MN229186	DMAN08
	DMAN MN229187	DMAN09
	DMAN MN229188	DMAN10
	DMAN MN229189	DMAN11
	DMAN MN229190	DMAN12
	DMAN MN229191	DMAN13
	DMAN MN229192	DMAN14
	DMAN MN229193	DMAN15
	DMAN MN229194	DMAN16
	DMAN MN229195	DMAN17
	DMAN MN229196	DMAN18
	DMAN MN229197	DMAN19
	DMAN MN229198	DMAN20
	DMAN MN229199	DMAN21
	DMAN MN229200	DMAN22
	DMAN MN229201	DMAN23
	DMAN MN229202	DMAN24
	DMAN MN229203	DMAN25
	DMAN MN229204	DMAN26
	DMAN MN229205	DMAN27
	DMAN MN229206	DMAN28
	DMAN MN229207	DMAN29

	SPANISH MERINI KF228822.1	SPAN25
	SPANISH MERINI KF228823.1	SPAN26
Timahdite	TIMAHDITE MN229241	TIM01
	TIMAHDITE MN229242	TIM02
	TIMAHDITE MN229243	TIM03
	TIMAHDITE MN229244	TIM04
	TIMAHDITE MN229245	TIM05
	TIMAHDITE MN229246	TIM06
	TIMAHDITE MN229247	TIM07
	TIMAHDITE MN229248	TIM08
	TIMAHDITE MN229249	TIM09
	TIMAHDITE MN229250	TIM10
	TIMAHDITE MN229251	TIM11
	TIMAHDITE MN229252	TIM12
	TIMAHDITE MN229253	TIM13
	TIMAHDITE MN229254	TIM14
	TIMAHDITE MN229255	TIM15
	TIMAHDITE MN229256	TIM16
	TIMAHDITE MN229257	TIM17
	TIMAHDITE MN229258	TIM18
	TIMAHDITE MN229259	TIM19
	TIMAHDITE MN229260	TIM20
	TIMAHDITE MN229261	TIM21
	TIMAHDITE MN229262	TIM22
	TIMAHDITE MN229263	TIM23
	TIMAHDITE MN229264	TIM24
	TIMAHDITE MN229265	TIM25
	TIMAHDITE MN229266	TIM26
	TIMAHDITE MN229267	TIM27
	TIMAHDITE MN229268	TIM28
	TIMAHDITE MN229269	TIM29
	TIMAHDITE MN229270	TIM30
	TIMAHDITE MN229271	TIM31
	TIMAHDITE MN229272	TIM32
	TIMAHDITE MN229273	TIM33
	TIMAHDITE MN229274	TIM34
	TIMAHDITE MN229275	TIM35
	TIMAHDITE MN229276	TIM36
	TIMAHDITE MN229277	TIM37

6. ANOVA results

Source	Type III SS	df	Mean Squares	F-Ratio	p-Value
**√(Alti)**					
**REGION**	10 788.328	2.000	5 394.164	84.356	0.000
**Error**	14 131.844	221.000	63.945		
**lg(T max)**					
**REGION**	0.575	2.000	0.287	109.465	0.000
**Error**	0.580	221.000	0.003		
**T min**					
**REGION**	29.570	2.000	14.785	2.415	0.092
**Error**	1 352.903	221.000	6.122		
**lg(M)**					
**REGION**	0.484	2.000	0.242	125.528	0.000
**Error**	0.426	221.000	0.002		
**M - m**					
**REGION**	4 509.628	2.000	2 254.814	135.978	0.000
**Error**	3 664.675	221.000	16.582		
**m**					
**REGION**	286.814	2.000	143.407	19.051	0.000
**Error**	1 663.605	221.000	7.528		
**R**					
**REGION**	10 785 437.156	2.000	5 392 718.578	115.215	0.000
**Error**	10 344 017.654	221.000	46 805.510		
**Q3**					
**REGION**	341 021.988	2.000	170 510.994	113.805	0.000
**Error**	331 119.717	221.000	1 498.279		

7. Climatic and genetic parameter

Data

	Climatic parameters								Genetic parameters							
Breeds	Alti	T max	T min	M	M - m	m	R	Q3	Hd	π	Fu and Li's F*	Fu and Li's D*	Fu's Fs	D of Tajima	τ	Shannon
Boujaad	648.333	26.390	11.087	38.933	35.500	3.433	461.244	44.575	0.994	0.02256	-3.280	-3.10524	-18.224	-2.111	7.48047	0.239
Blanche de Montagne	1430.867	23.907	7.804	35.193	35.827	-0.900	321.400	30.986	0.97	0.0133	-3.016	-2.9434	-13.24	-1.766	8.09375	0.139
Beni-Guil	924.400	22.267	9.277	32.507	30.533	1.973	263.324	30.029	0.966	0.0164	-2.931	-2.74106	-22.713	-1.953	7.58984	0.143
D'man	998.250	28.099	12.448	40.400	38.513	1.888	110.227	9.859	0.963	0.01514	-0.880	-0.63376	-7.424	-1.744	9.5293	0.000
Sardi	430.158	25.299	11.667	34.868	30.021	4.847	334.088	38.380	0.994	0.02028	-1.821	-1.45748	-18.582	-1.692	7.12305	0.229
Timahdite	1010.000	22.281	8.439	33.683	32.448	1.235	578.634	60.947	0.988	0.02151	-2.502	-2.2419	-25.845	-1.855	6.25391	0.281
Campaniça	226.600	21.443	11.105	30.870	24.950	5.920	607.800	83.759	0.968	0.0207	-1.04632	-0.67904	-2.651	-1.29999	8.50977	0.000
Churra Algarvia	82.900	21.347	12.271	28.520	20.920	7.600	509.100	83.758	0.98	0.01419	-0.67085	-0.41733	-4.627	-0.96957	9.32422	0.389
Churra Badana	525.300	18.423	7.663	28.980	27.540	1.440	743.700	92.716	0.972	0.00924	-2.42002	-2.1754	-15.157	-1.80017	8.97656	0.000
Churra de terra Quente	515.600	18.529	7.956	28.980	27.140	1.840	839.600	106.337	0.994	0.01465	-2.39266	-2.12604	-18.069	-1.817	8.81836	0.168
Latxa Black Face	328.364	16.988	8.642	24.682	21.345	3.336	1086.182	179.734	0.993	0.01783	-2.57006	-2.31506	-11.269	-1.90879	6.9082	0.143
Latxa Blonde Face	222.818	17.770	9.216	25.327	21.173	4.155	1150.818	189.761	0.989	0.01325	-1.76605	-1.4832	-8.782	-1.58999	6.05469	0.000
Saloia	169.727	19.686	11.842	26.709	19.473	7.236	742.091	136.118	0.995	0.01737	-1.35743	-0.92374	-18.953	-1.60424	8.1875	0.000
Spanish Merino	404.750	22.340	10.570	34.313	30.650	3.663	533.625	60.140	1	0.01391	-2.52843	-2.26675	-22.036	-1.89837	8.23633	0.245
Appenninica	214.429	18.435	9.136	28.950	27.393	1.557	797.643	99.953	0.98	0.01436	-1.82815	-1.52735	-8.197	-1.59373	8.82812	0.000
Comisana	328.650	19.586	12.040	28.815	23.140	5.675	693.750	103.559	0.94	0.03278	0.17612	0.00182	-0.655	0.4461	5.25	0.580
Gentile di Puglia	333.600	20.047	11.876	29.513	24.540	4.973	685.200	98.107	0.995	0.01466	-2.17776	-1.92966	-19.863	-1.66853	8.58398	0.000
Merinizzata Italiana	370.800	18.745	10.276	28.675	25.765	2.910	686.350	91.774	1	0.01247	-2.15818	-1.81381	-29.76	-1.85405	8.80664	0.146
Sarda	267.525	18.208	9.649	28.425	26.170	2.255	765.450	100.102	0.989	0.01723	-2.12499	-1.77277	-12.742	-1.84739	8.40234	0.000
Sopravissana	264.962	18.254	9.268	28.631	26.835	1.796	831.308	106.283	0.977	0.0126	-1.72346	-1.45472	-8.867	-1.47031	9.17969	0.000

Normalized Data

	Climatic parameters								Genetic parameters							
Breeds	√(Alti)	lg(T max)	T min	lg(M)	M - m	m	R	Q3	Hd	lg(π)	Fu and Li's F*	Fu and Li's D*	Fu's Fs	D of Tajima	τ	Shannon
Boujaad	25.462	1.421	11.087	1.590	35.500	3.433	461.244	44.575	0.994	-1.647	-3.280	-3.105	-18.224	-2.111	7.480	0.239
Blanche de Montagne	37.827	1.379	7.804	1.546	35.827	-0.900	321.400	30.986	0.970	-1.876	-3.016	-2.943	-13.240	-1.766	8.094	0.139
Beni-Guil	30.404	1.348	9.277	1.512	30.533	1.973	263.324	30.029	0.966	-1.785	-2.931	-2.741	-22.713	-1.953	7.590	0.143
D'man	31.595	1.449	12.448	1.606	38.513	1.888	110.227	9.859	0.963	-1.820	-0.880	-0.634	-7.424	-1.744	9.529	0.000
Sardi	20.740	1.403	11.667	1.542	30.021	4.847	334.088	38.380	0.994	-1.693	-1.821	-1.457	-18.582	-1.692	7.123	0.229
Timahdite	31.780	1.348	8.439	1.527	32.448	1.235	578.634	60.947	0.988	-1.667	-2.502	-2.242	-25.845	-1.855	6.254	0.281
Campaniça	15.053	1.331	11.105	1.490	24.950	5.920	607.800	83.759	0.968	-1.684	-1.046	-0.679	-2.651	-1.300	8.510	0.000
Churra Algarvia	9.105	1.329	12.271	1.455	20.920	7.600	509.100	83.758	0.980	-1.848	-0.671	-0.417	-4.627	-0.970	9.324	0.389
Churra Badana	22.919	1.265	7.663	1.462	27.540	1.440	743.700	92.716	0.972	-2.034	-2.420	-2.175	-15.157	-1.800	8.977	0.000
Churra de terra Quente	22.707	1.268	7.956	1.462	27.140	1.840	839.600	106.337	0.994	-1.834	-2.393	-2.126	-18.069	-1.817	8.818	0.168
Latxa Black Face	18.121	1.230	8.642	1.392	21.345	3.336	1086.182	179.734	0.993	-1.749	-2.570	-2.315	-11.269	-1.909	6.908	0.143
Latxa Blonde Face	14.927	1.250	9.216	1.404	21.173	4.155	1150.818	189.761	0.989	-1.878	-1.766	-1.483	-8.782	-1.590	6.055	0.000
Saloia	13.028	1.294	11.842	1.427	19.473	7.236	742.091	136.118	0.995	-1.760	-1.357	-0.924	-18.953	-1.604	8.188	0.245
Spanish Merino	20.118	1.349	10.570	1.535	30.650	3.663	533.625	60.140	1.000	-1.857	-2.528	-2.267	-22.036	-1.898	8.236	0.000
Appenninica	12.617	1.276	9.188	1.468	27.380	2.020	781.800	97.739	0.980	-1.843	-1.828	-1.527	-8.197	-1.594	8.828	0.000
Comisana	19.154	1.294	12.507	1.458	22.488	6.213	545.500	83.862	0.940	-1.484	0.176	0.002	-0.655	0.446	5.250	0.580
Gentile di Puglia	16.544	1.260	11.064	1.446	22.986	4.957	540.571	110.600	0.995	-1.834	-2.178	-1.930	-19.863	-1.669	8.584	0.000
Merinizzata Italiana	19.256	1.273	10.276	1.458	25.765	2.910	686.350	91.774	1.000	-1.904	-2.158	-1.814	-29.760	-1.854	8.807	0.146
Sarda	11.421	1.301	12.360	1.459	22.286	6.500	548.143	83.938	0.989	-1.764	-2.125	-1.773	-12.742	-1.847	8.402	0.000
Sopravissana	17.163	1.242	9.524	1.445	26.286	1.586	824.143	107.514	0.977	-1.900	-1.723	-1.455	-8.867	-1.470	9.180	0.000

Correlation table

	Climatic parameters								Genetic parameters							
	√(Alti)	lg(T max)	T min	lg(M)	M - m	m	R	Q3	Hd	lg(π)	Fu and Li's F*	Fu and Li's D*	Fu's Fs	D of Tajima	τ	Shannon
√(Alti)	0.000	0.010	0.071	0.001	0.000	0.000	0.019	0.004	0.285	0.925	0.046	0.020	0.151	0.217	0.563	0.849
lg(T max)	0.559	0.000	0.129	0.000	0.000	0.650	0.000	0.000	0.499	0.185	0.798	0.745	0.757	0.522	0.909	0.517
T min	-0.412	0.351	0.000	0.498	0.423	0.000	0.068	0.373	0.606	0.036	0.003	0.002	0.224	0.066	0.886	0.227
lg(M)	0.677	0.939	0.161	0.000	0.000	0.127	0.000	0.000	0.524	0.416	0.369	0.310	0.453	0.300	0.645	0.966
M - m	0.839	0.775	-0.190	0.920	0.000	0.001	0.002	0.000	0.557	0.941	0.076	0.052	0.287	0.092	0.593	0.570
m	-0.758	-0.108	0.777	-0.353	-0.688	0.000	0.759	0.220	0.756	0.076	0.007	0.004	0.205	0.046	0.629	0.158
R	-0.519	-0.841	-0.416	-0.817	-0.650	0.073	0.000	0.000	0.146	0.347	0.895	0.994	0.651	0.986	0.425	0.501
Q3	-0.617	-0.846	-0.210	-0.903	-0.800	0.287	0.939	0.000	0.174	0.494	0.663	0.556	0.479	0.731	0.381	0.632
Hd	-0.251	-0.160	-0.123	-0.151	-0.140	0.074	0.337	0.316	0.000	0.224	0.017	0.057	0.002	0.001	0.514	0.222
lg(π)	0.023	0.309	0.472	0.193	-0.018	0.406	-0.222	-0.162	-0.285	0.000	0.164	0.248	0.388	0.028	0.002	0.002
Fu and Li's F*	-0.451	-0.061	0.629	-0.212	-0.405	0.585	-0.032	0.104	-0.525	0.324	0.000	0.000	0.001	0.000	0.871	0.112
Fu and Li's D*	-0.516	-0.078	0.642	-0.239	-0.440	0.621	0.002	0.140	-0.432	0.271	0.989	0.000	0.003	0.000	0.900	0.212
Fu's Fs	-0.333	-0.074	0.285	-0.178	-0.250	0.296	0.108	0.168	-0.639	0.204	0.674	0.636	0.000	0.002	0.942	0.713
D of Tajima	-0.289	-0.152	0.419	-0.244	-0.387	0.451	-0.004	0.082	-0.672	0.490	0.824	0.744	0.638	0.000	0.128	0.003
τ	-0.138	0.027	0.034	0.110	0.127	-0.115	-0.189	-0.207	0.155	-0.655	-0.039	0.030	-0.017	-0.352	0.000	0.021
Shannon	0.045	0.154	0.283	0.010	-0.135	0.328	-0.160	-0.114	-0.286	0.646	0.367	0.292	0.088	0.624	-0.512	0.000

8. Calibration and dating

	Breeds	τ	τ qt 5%	τ qt 95%	Date	5%	95%
Morocco	Blanche de Monatgne	8.1	5.6	9.3	7665	6373	8303
	Boujaad	7.5	4.9	15.3	7352	6015	11350
	Beni-Guil	7.6	5.6	12.7	7408	6391	10035
	D'man	9.5	5.4	11.8	8398	6310	9539
	Sardi	7.1	5.5	11.9	7170	6330	9612
	Timahdite	6.3	3.6	13.9	6726	5365	10641
Iberia	Campaniça	8.5	6.0	10.9	7877	6618	9088
	Churra Algarvia	9.3	6.3	10.8	8293	6770	9020
	Churra Badana	9.0	6.4	10.4	8116	6810	8838
	Churra de Terra Quente	8.8	5.9	10.9	8035	6526	9076
	Latxa blonde face	6.1	4.6	7.2	6625	5859	7214
	Latxa Black Face	6.9	4.5	8.9	7060	5815	8102
	Saloia	8.2	6.7	9.2	7713	6950	8243
	Spanish Merino	8.2	6.6	9.5	7738	6885	8390
Italy	Appenninica	8.8	5.7	10.1	8040	6456	8701
	Gentile de Puglia	8.6	7.1	9.6	7915	7134	8415
	Merinizzata Italiana	8.8	6.2	9.8	8029	6721	8554
	Lacaune of Italy	7.6	6.2	8.6	7414	6720	7928
	Sopravissana	9.2	7.3	10.7	8219	7242	9011
	Sarda	8.4	5.0	10.7	7823	6100	9011
Morocan group 1		7.0	5.3	11.4	7131	6224	9351
Moroccan group 2		9.9	6.8	11.6	8605	7024	9453
Iberian group		7.8	7.1	8.1	7503	7134	7688
Italian group		8.8	8.0	9.2	8002	7638	8229

Supplementary Data 2: Phylogenetic tree

Evolutionary analysis by Maximum Likelihood method

The evolutionary history was inferred by using the Maximum Likelihood method and Hasegawa-Kishino-Yano mode[40]. The tree with the highest log likelihood (-23,775.91) is shown. Initial tree(s) for the heuristic search were obtained automatically by applying Neighbor- Join and BioNJ algorithms to a matrix of pairwise distances estimated using the Maximum Composite Likelihood (MCL) approach, and then selecting the topology with superior log likelihood value. A discrete Gamma distribution was used to model evolutionary rate differences among sites (5 categories (+*G*, parameter = 0.4180)). The rate variation model allowed for some sites to be evolutionarily invariable ([+*I*], 21.18% sites). The tree is drawn to scale, with branch lengths measured in the number of substitutions per site. This analysis involved 845 nucleotide sequences. There was a total of 628 positions in the final dataset. Evolutionary analyses were conducted in MEGA X[32].

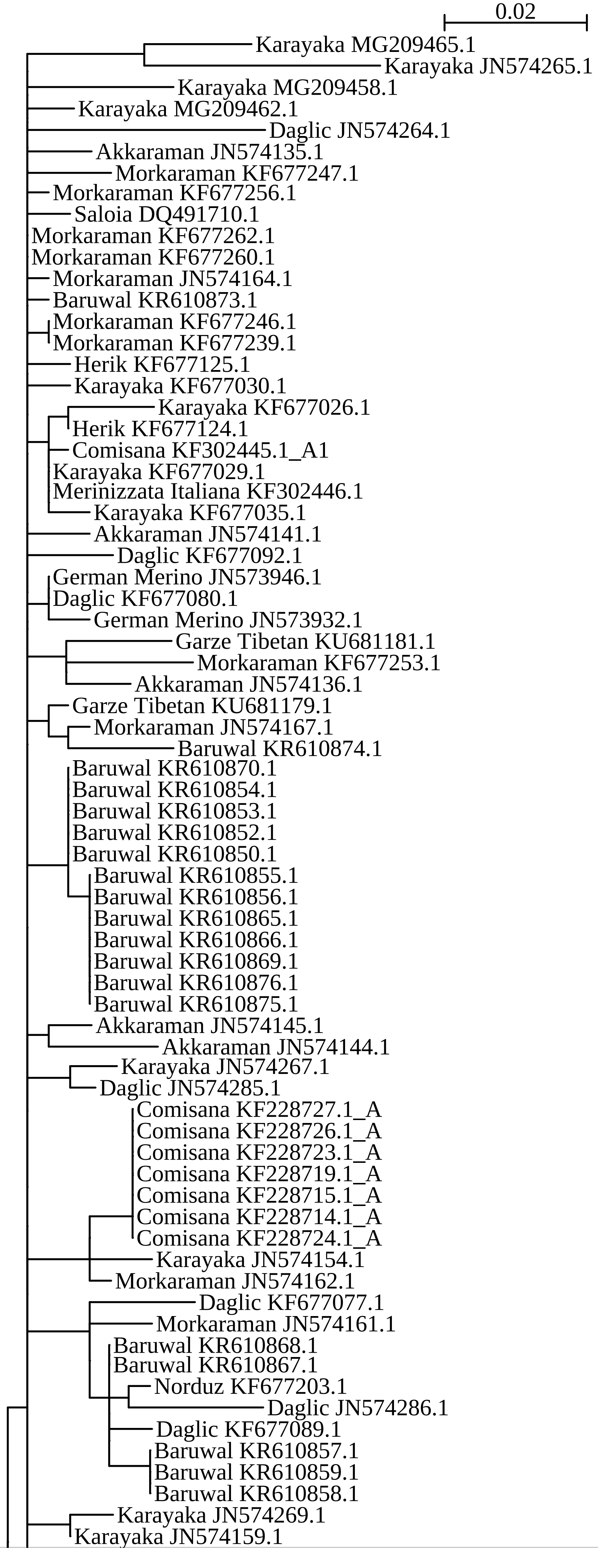
0.02
Karayaka MG209465.1
Karayaka JN574265.1
Karayaka MG209458.1
Karayaka MG209462.1
Daglic JN574264.1
Akkaraman JN574135.1
Morkaraman KF677247.1
Morkaraman KF677256.1
Saloia DQ491710.1
Morkaraman KF677262.1
Morkaraman KF677260.1
Morkaraman JN574164.1
Baruwal KR610873.1
Morkaraman KF677246.1
Morkaraman KF677239.1
Herik KF677125.1
Karayaka KF677030.1
Karayaka KF677026.1
Herik KF677124.1
Comisana KF302445.1_A1
Karayaka KF677029.1
Merinizzata Italiana KF302446.1
Karayaka KF677035.1
Akkaraman JN574141.1
Daglic KF677092.1
German Merino JN573946.1
Daglic KF677080.1
German Merino JN573932.1
Garze Tibetan KU681181.1
Morkaraman KF677253.1
Akkaraman JN574136.1
Garze Tibetan KU681179.1
Morkaraman JN574167.1
Baruwal KR610874.1
Baruwal KR610870.1
Baruwal KR610854.1
Baruwal KR610853.1
Baruwal KR610852.1
Baruwal KR610850.1
Baruwal KR610855.1
Baruwal KR610856.1
Baruwal KR610865.1
Baruwal KR610866.1
Baruwal KR610869.1
Baruwal KR610876.1
Baruwal KR610875.1
Akkaraman JN574145.1
Akkaraman JN574144.1
Karayaka JN574267.1
Daglic JN574285.1
Comisana KF228727.1_A
Comisana KF228726.1_A
Comisana KF228723.1_A
Comisana KF228719.1_A
Comisana KF228715.1_A
Comisana KF228714.1_A
Comisana KF228724.1_A
Karayaka JN574154.1
Morkaraman JN574162.1
Daglic KF677077.1
Morkaraman JN574161.1
Baruwal KR610868.1
Baruwal KR610867.1
Norduz KF677203.1
Daglic JN574286.1
Daglic KF677089.1
Baruwal KR610857.1
Baruwal KR610859.1
Baruwal KR610858.1
Karayaka JN574269.1
Karayaka JN574159.1

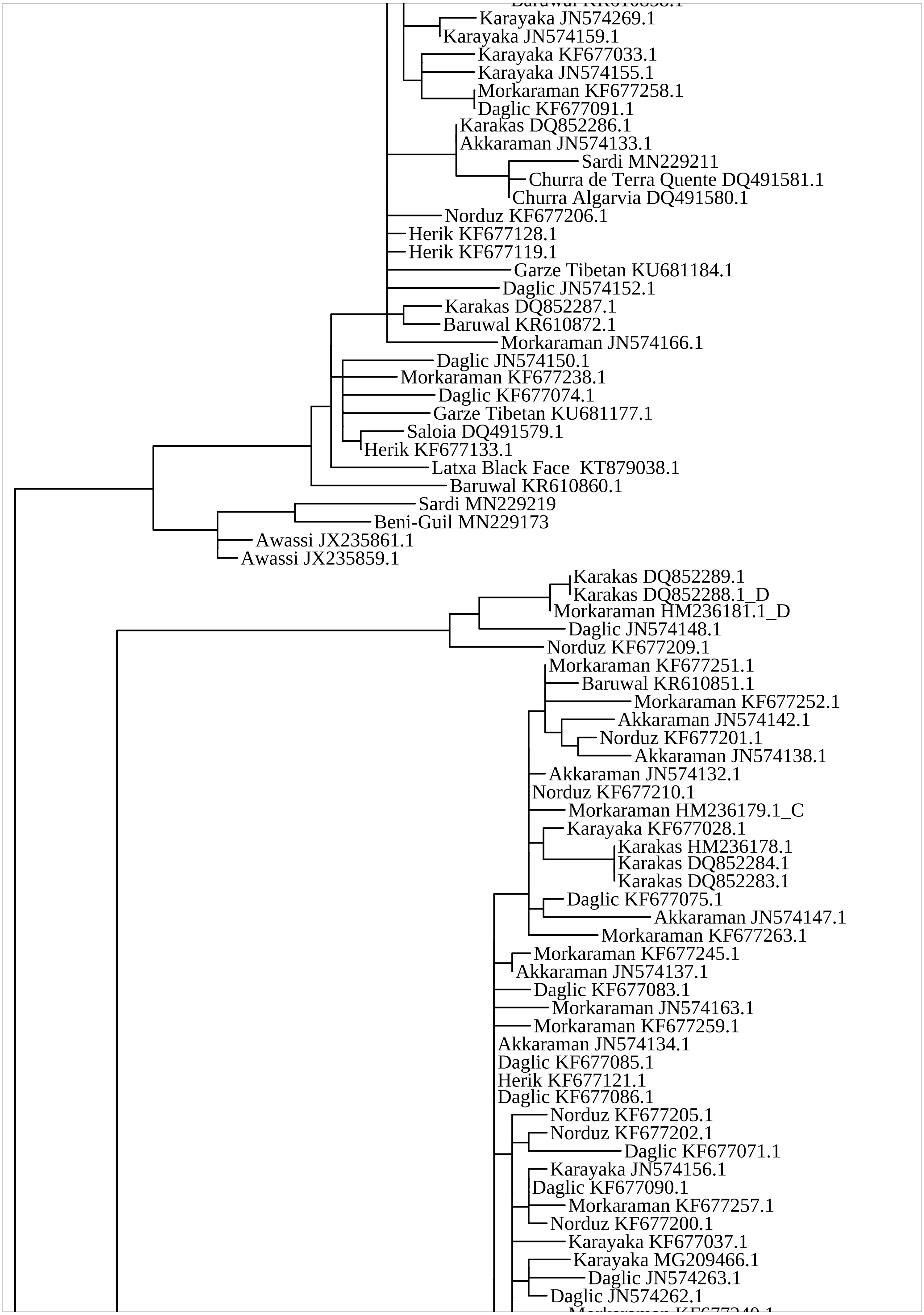
Karayaka JN574269.1
Karayaka JN574159.1
Karayaka KF677033.1
Karayaka JN574155.1
Morkaraman KF677258.1
Daglic KF677091.1
Karakas DQ852286.1
Akkaraman JN574133.1
Sardi MN229211
Churra de Terra Quente DQ491581.1
Churra Algarvia DQ491580.1
Norduz KF677206.1
Herik KF677128.1
Herik KF677119.1
Garze Tibetan KU681184.1
Daglic JN574152.1
Karakas DQ852287.1
Baruwal KR610872.1
Morkaraman JN574166.1
Daglic JN574150.1
Morkaraman KF677238.1
Daglic KF677074.1
Garze Tibetan KU681177.1
Saloia DQ491579.1
Herik KF677133.1
Latxa Black Face KT879038.1
Baruwal KR610860.1
Sardi MN229219
Beni-Guil MN229173
Awassi JX235861.1
Awassi JX235859.1
Karakas DQ852289.1
Karakas DQ852288.1_D
Morkaraman HM236181.1_D
Daglic JN574148.1
Norduz KF677209.1
Morkaraman KF677251.1
Baruwal KR610851.1
Morkaraman KF677252.1
Akkaraman JN574142.1
Norduz KF677201.1
Akkaraman JN574138.1
Akkaraman JN574132.1
Norduz KF677210.1
Morkaraman HM236179.1_C
Karayaka KF677028.1
Karakas HM236178.1
Karakas DQ852284.1
Karakas DQ852283.1
Daglic KF677075.1
Akkaraman JN574147.1
Morkaraman KF677263.1
Morkaraman KF677245.1
Akkaraman JN574137.1
Daglic KF677083.1
Morkaraman JN574163.1
Morkaraman KF677259.1
Akkaraman JN574134.1
Daglic KF677085.1
Herik KF677121.1
Daglic KF677086.1
Norduz KF677205.1
Norduz KF677202.1
Daglic KF677071.1
Karayaka JN574156.1
Daglic KF677090.1
Morkaraman KF677257.1
Norduz KF677200.1
Karayaka KF677037.1
Karayaka MG209466.1
Daglic JN574263.1
Daglic JN574262.1

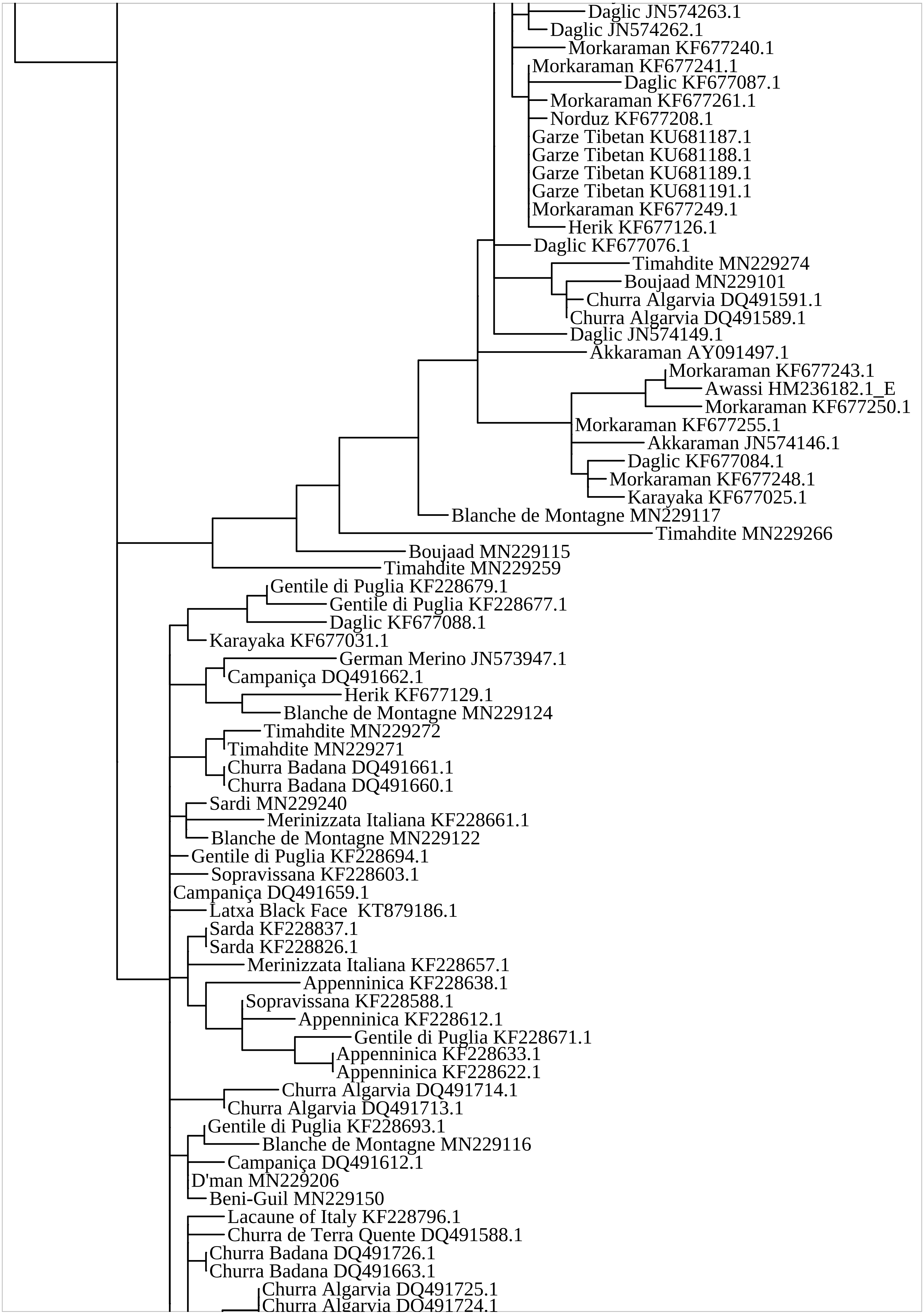
Daglic JN574263.1
Daglic JN574262.1
Morkaraman KF677240.1
Morkaraman KF677241.1
Daglic KF677087.1
Morkaraman KF677261.1
Norduz KF677208.1
Garze Tibetan KU681187.1
Garze Tibetan KU681188.1
Garze Tibetan KU681189.1
Garze Tibetan KU681191.1
Morkaraman KF677249.1
Herik KF677126.1
Daglic KF677076.1
Timahdite MN229274
Boujaad MN229101
Churra Algarvia DQ491591.1
Churra Algarvia DQ491589.1
Daglic JN574149.1
Akkaraman AY091497.1
Morkaraman KF677243.1
Awassi HM236182.1_E
Morkaraman KF677250.1
Morkaraman KF677255.1
Akkaraman JN574146.1
Daglic KF677084.1
Morkaraman KF677248.1
Karayaka KF677025.1
Blanche de Montagne MN229117
Timahdite MN229266
Boujaad MN229115
Timahdite MN229259
Gentile di Puglia KF228679.1
Gentile di Puglia KF228677.1
Daglic KF677088.1
Karayaka KF677031.1
German Merino JN573947.1
Campaniça DQ491662.1
Herik KF677129.1
Blanche de Montagne MN229124
Timahdite MN229272
Timahdite MN229271
Churra Badana DQ491661.1
Churra Badana DQ491660.1
Sardi MN229240
Merinizzata Italiana KF228661.1
Blanche de Montagne MN229122
Gentile di Puglia KF228694.1
Sopravissana KF228603.1
Campaniça DQ491659.1
Latxa Black Face KT879186.1
Sarda KF228837.1
Sarda KF228826.1
Merinizzata Italiana KF228657.1
Appenninica KF228638.1
Sopravissana KF228588.1
Appenninica KF228612.1
Gentile di Puglia KF228671.1
Appenninica KF228633.1
Appenninica KF228622.1
Churra Algarvia DQ491714.1
Churra Algarvia DQ491713.1
Gentile di Puglia KF228693.1
Blanche de Montagne MN229116
Campaniça DQ491612.1
D'man MN229206
Beni-Guil MN229150
Lacaune of Italy KF228796.1
Churra de Terra Quente DQ491588.1
Churra Badana DQ491726.1
Churra Badana DQ491663.1
Churra Algarvia DQ491725.1
Churra Algarvia DQ491724.1

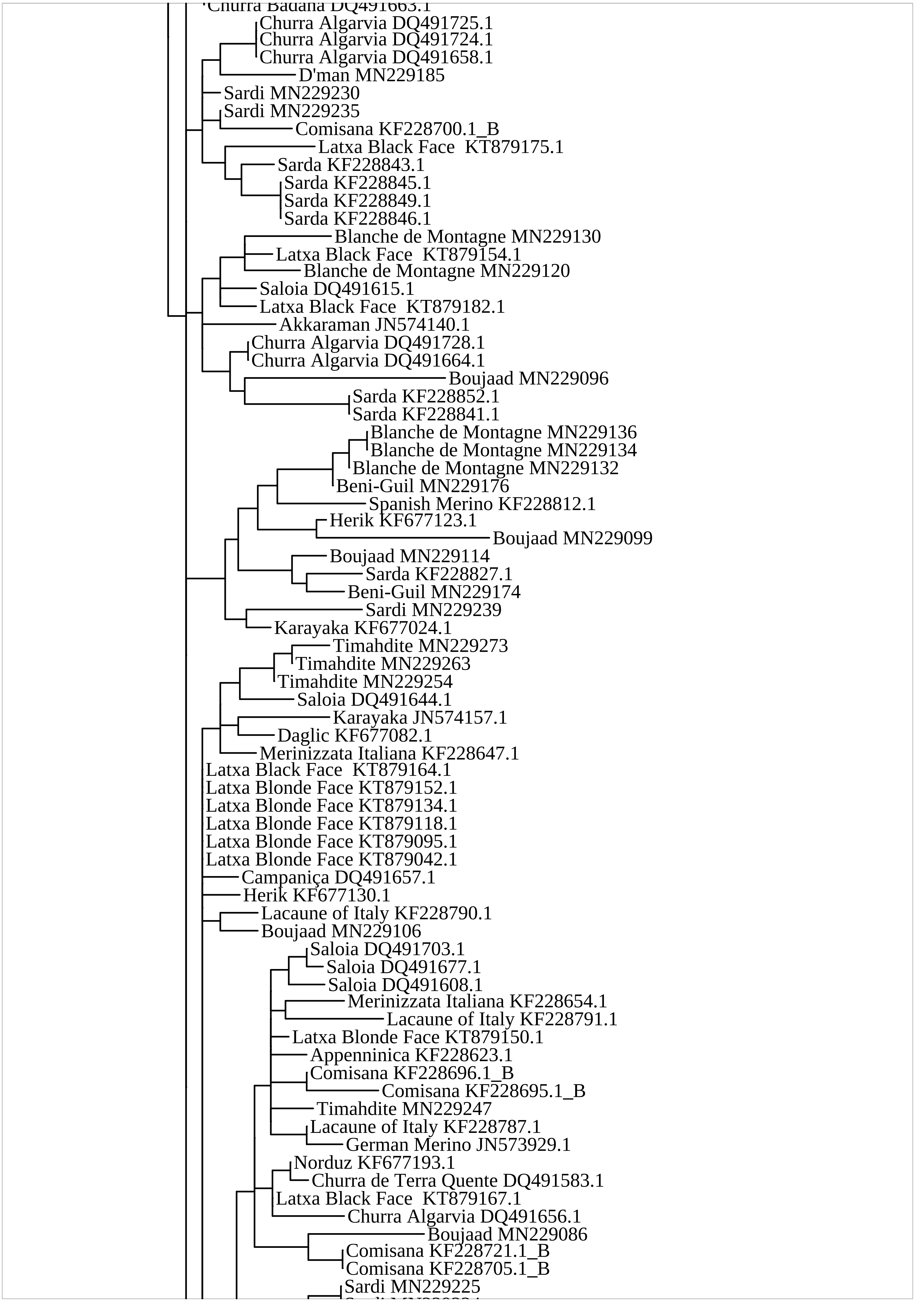
Churra Algarvia DQ491725.1
Churra Algarvia DQ491724.1
Churra Algarvia DQ491658.1
D'man MN229185
Sardi MN229230
Sardi MN229235
Comisana KF228700.1_B
Latxa Black Face KT879175.1
Sarda KF228843.1
Sarda KF228845.1
Sarda KF228849.1
Sarda KF228846.1
Blanche de Montagne MN229130
Latxa Black Face KT879154.1
Blanche de Montagne MN229120
Saloia DQ491615.1
Latxa Black Face KT879182.1
Akkaraman JN574140.1
Churra Algarvia DQ491728.1
Churra Algarvia DQ491664.1
Boujaad MN229096
Sarda KF228852.1
Sarda KF228841.1
Blanche de Montagne MN229136
Blanche de Montagne MN229134
Blanche de Montagne MN229132
Beni-Guil MN229176
Spanish Merino KF228812.1
Herik KF677123.1
Boujaad MN229099
Boujaad MN229114
Sarda KF228827.1
Beni-Guil MN229174
Sardi MN229239
Karayaka KF677024.1
Timahdite MN229273
Timahdite MN229263
Timahdite MN229254
Saloia DQ491644.1
Karayaka JN574157.1
Daglic KF677082.1
Merinizzata Italiana KF228647.1
Latxa Black Face KT879164.1
Latxa Blonde Face KT879152.1
Latxa Blonde Face KT879134.1
Latxa Blonde Face KT879118.1
Latxa Blonde Face KT879095.1
Latxa Blonde Face KT879042.1
Campaniça DQ491657.1
Herik KF677130.1
Lacaune of Italy KF228790.1
Boujaad MN229106
Saloia DQ491703.1
Saloia DQ491677.1
Saloia DQ491608.1
Merinizzata Italiana KF228654.1
Lacaune of Italy KF228791.1
Latxa Blonde Face KT879150.1
Appenninica KF228623.1
Comisana KF228696.1_B
Comisana KF228695.1_B
Timahdite MN229247
Lacaune of Italy KF228787.1
German Merino JN573929.1
Norduz KF677193.1
Churra de Terra Quente DQ491583.1
Latxa Black Face KT879167.1
Churra Algarvia DQ491656.1
Boujaad MN229086
Comisana KF228721.1_B
Comisana KF228705.1_B
Sardi MN229225

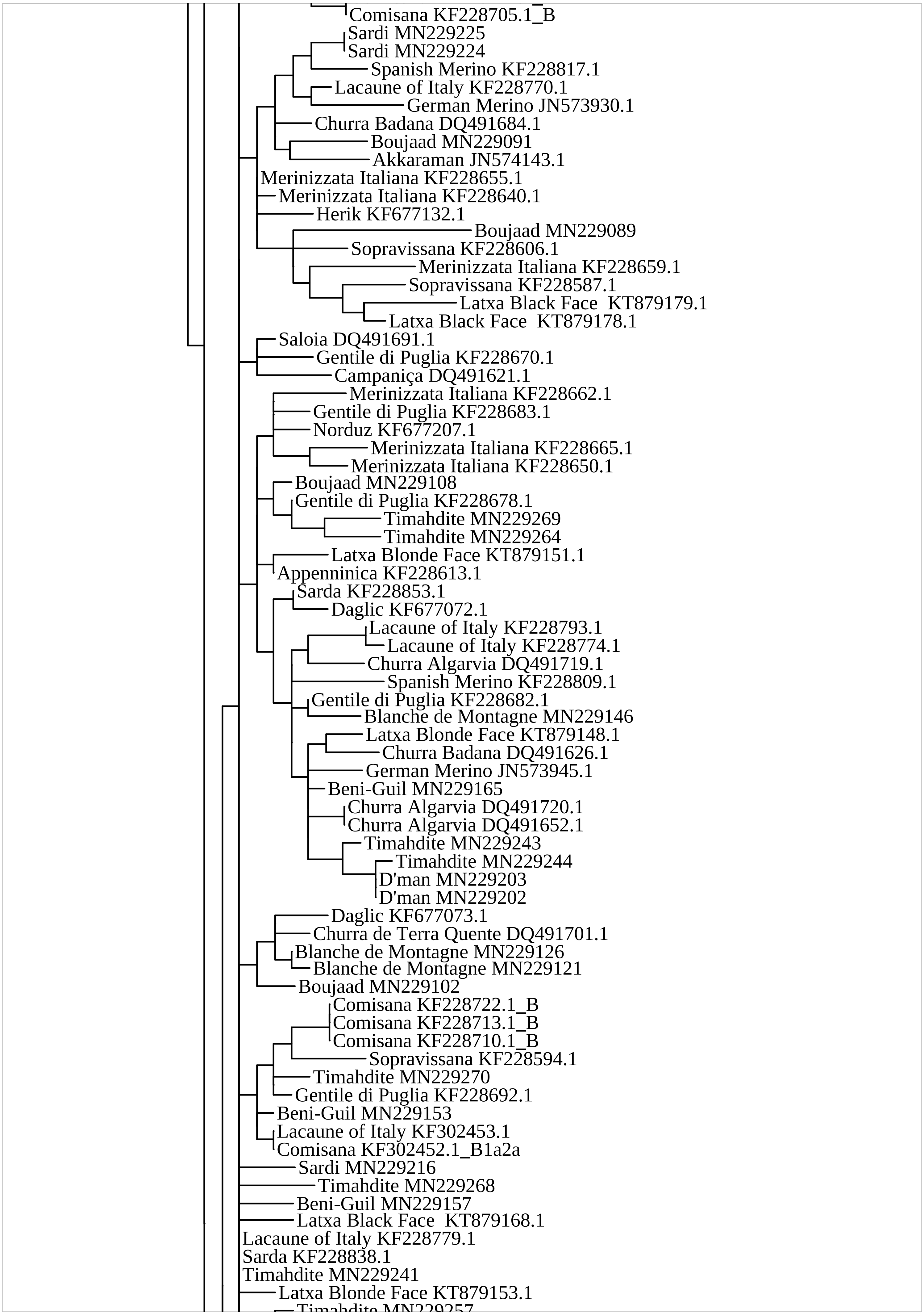
Comisana KF228705.1_B
Sardi MN229225
Sardi MN229224
Spanish Merino KF228817.1
Lacaune of Italy KF228770.1
German Merino JN573930.1
Churra Badana DQ491684.1
Boujaad MN229091
Akkaraman JN574143.1
Merinizzata Italiana KF228655.1
Merinizzata Italiana KF228640.1
Herik KF677132.1
Boujaad MN229089
Sopravissana KF228606.1
Merinizzata Italiana KF228659.1
Sopravissana KF228587.1
Latxa Black Face KT879179.1
Latxa Black Face KT879178.1
Saloia DQ491691.1
Gentile di Puglia KF228670.1
Campaniça DQ491621.1
Merinizzata Italiana KF228662.1
Gentile di Puglia KF228683.1
Norduz KF677207.1
Merinizzata Italiana KF228665.1
Merinizzata Italiana KF228650.1
Boujaad MN229108
Gentile di Puglia KF228678.1
Timahdite MN229269
Timahdite MN229264
Latxa Blonde Face KT879151.1
Appenninica KF228613.1
Sarda KF228853.1
Daglic KF677072.1
Lacaune of Italy KF228793.1
Lacaune of Italy KF228774.1
Churra Algarvia DQ491719.1
Spanish Merino KF228809.1
Gentile di Puglia KF228682.1
Blanche de Montagne MN229146
Latxa Blonde Face KT879148.1
Churra Badana DQ491626.1
German Merino JN573945.1
Beni-Guil MN229165
Churra Algarvia DQ491720.1
Churra Algarvia DQ491652.1
Timahdite MN229243
Timahdite MN229244
D'man MN229203
D'man MN229202
Daglic KF677073.1
Churra de Terra Quente DQ491701.1
Blanche de Montagne MN229126
Blanche de Montagne MN229121
Boujaad MN229102
Comisana KF228722.1_B
Comisana KF228713.1_B
Comisana KF228710.1_B
Sopravissana KF228594.1
Timahdite MN229270
Gentile di Puglia KF228692.1
Beni-Guil MN229153
Lacaune of Italy KF302453.1
Comisana KF302452.1_B1a2a
Sardi MN229216
Timahdite MN229268
Beni-Guil MN229157
Latxa Black Face KT879168.1
Lacaune of Italy KF228779.1
Sarda KF228838.1
Timahdite MN229241
Latxa Blonde Face KT879153.1

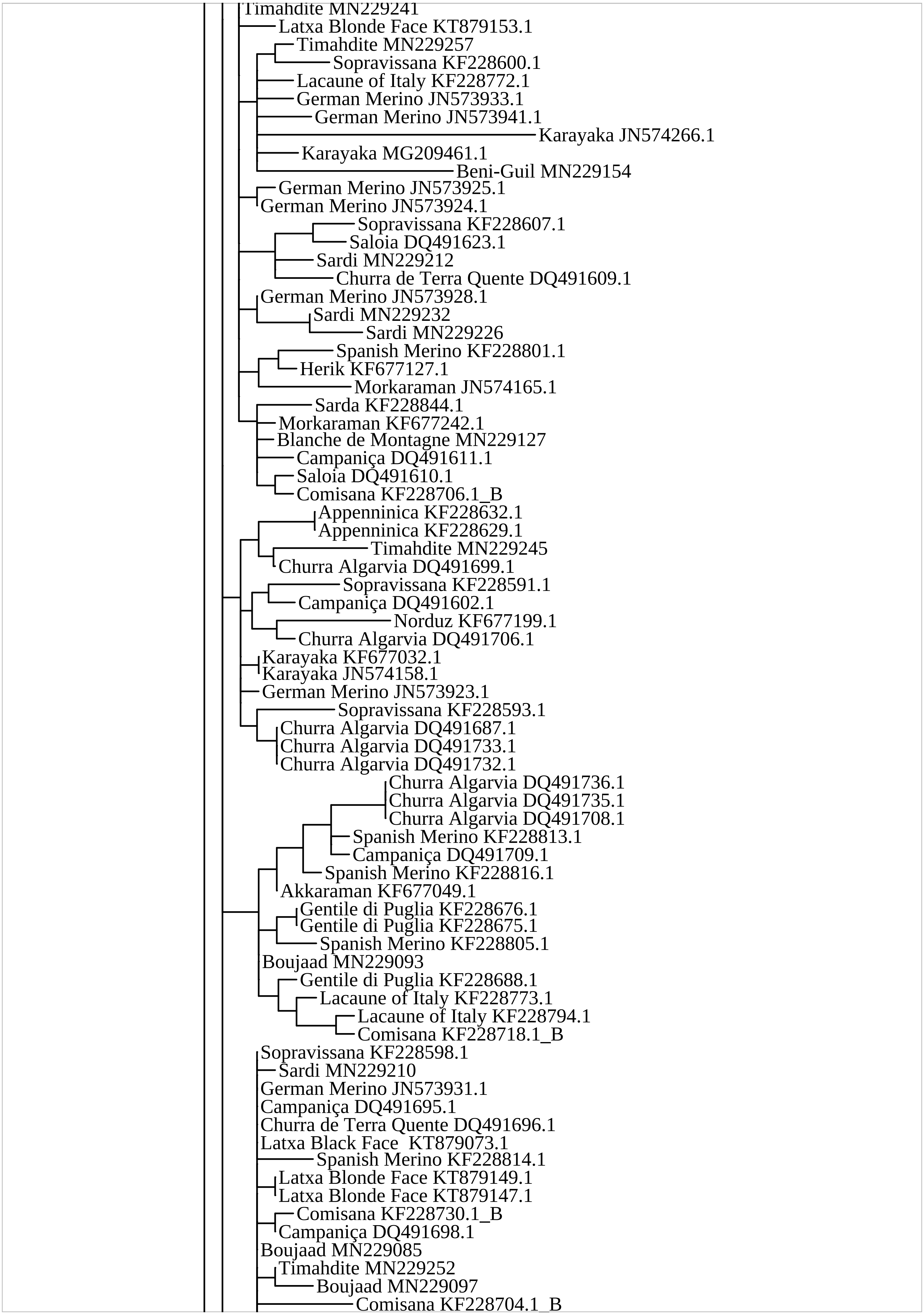
Timahdite MN229241
Latxa Blonde Face KT879153.1
Timahdite MN229257
Sopravissana KF228600.1
Lacaune of Italy KF228772.1
German Merino JN573933.1
German Merino JN573941.1
Karayaka JN574266.1
Karayaka MG209461.1
Beni-Guil MN229154
German Merino JN573925.1
German Merino JN573924.1
Sopravissana KF228607.1
Saloia DQ491623.1
Sardi MN229212
Churra de Terra Quente DQ491609.1
German Merino JN573928.1
Sardi MN229232
Sardi MN229226
Spanish Merino KF228801.1
Herik KF677127.1
Morkaraman JN574165.1
Sarda KF228844.1
Morkaraman KF677242.1
Blanche de Montagne MN229127
Campaniça DQ491611.1
Saloia DQ491610.1
Comisana KF228706.1_B
Appenninica KF228632.1
Appenninica KF228629.1
Timahdite MN229245
Churra Algarvia DQ491699.1
Sopravissana KF228591.1
Campaniça DQ491602.1
Norduz KF677199.1
Churra Algarvia DQ491706.1
Karayaka KF677032.1
Karayaka JN574158.1
German Merino JN573923.1
Sopravissana KF228593.1
Churra Algarvia DQ491687.1
Churra Algarvia DQ491733.1
Churra Algarvia DQ491732.1
Churra Algarvia DQ491736.1
Churra Algarvia DQ491735.1
Churra Algarvia DQ491708.1
Spanish Merino KF228813.1
Campaniça DQ491709.1
Spanish Merino KF228816.1
Akkaraman KF677049.1
Gentile di Puglia KF228676.1
Gentile di Puglia KF228675.1
Spanish Merino KF228805.1
Boujaad MN229093
Gentile di Puglia KF228688.1
Lacaune of Italy KF228773.1
Lacaune of Italy KF228794.1
Comisana KF228718.1_B
Sopravissana KF228598.1
Sardi MN229210
German Merino JN573931.1
Campaniça DQ491695.1
Churra de Terra Quente DQ491696.1
Latxa Black Face KT879073.1
Spanish Merino KF228814.1
Latxa Blonde Face KT879149.1
Latxa Blonde Face KT879147.1
Comisana KF228730.1_B
Campaniça DQ491698.1
Boujaad MN229085
Timahdite MN229252
Boujaad MN229097
Comisana KF228704.1_B

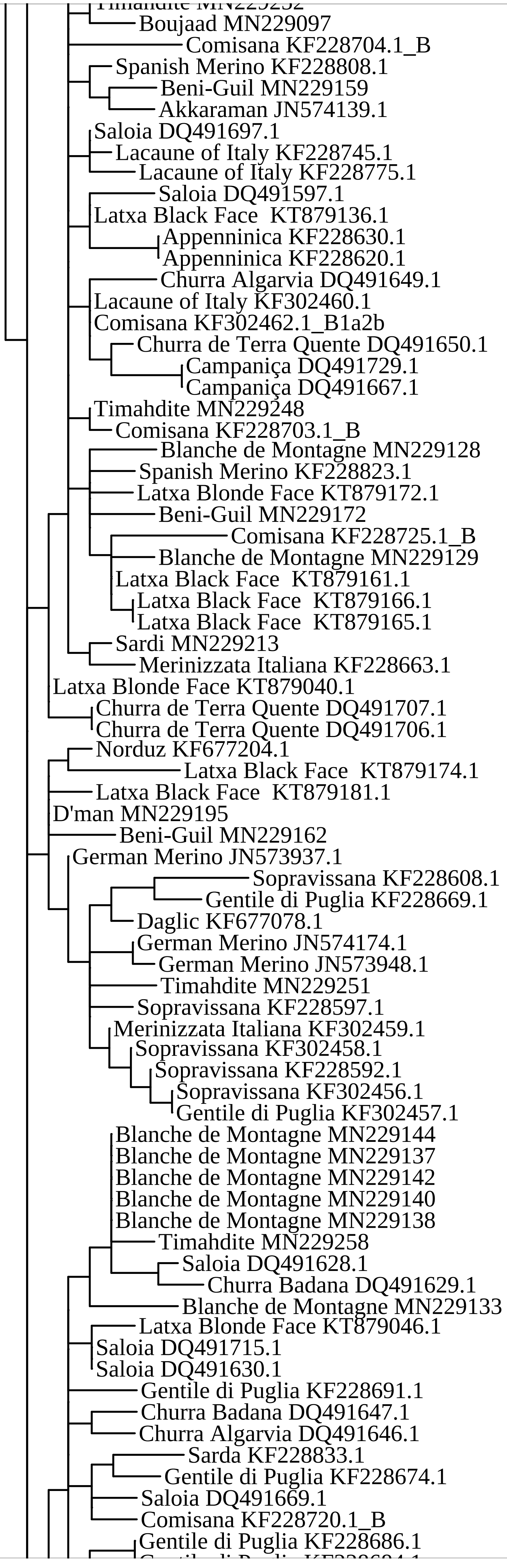
Boujaad MN229097
Comisana KF228704.1_B
Spanish Merino KF228808.1
Beni-Guil MN229159
Akkaraman JN574139.1
Saloia DQ491697.1
Lacaune of Italy KF228745.1
Lacaune of Italy KF228775.1
Saloia DQ491597.1
Latxa Black Face KT879136.1
Appenninica KF228630.1
Appenninica KF228620.1
Churra Algarvia DQ491649.1
Lacaune of Italy KF302460.1
Comisana KF302462.1_B1a2b
Churra de Terra Quente DQ491650.1
Campaniça DQ491729.1
Campaniça DQ491667.1
Timahdite MN229248
Comisana KF228703.1_B
Blanche de Montagne MN229128
Spanish Merino KF228823.1
Latxa Blonde Face KT879172.1
Beni-Guil MN229172
Comisana KF228725.1_B
Blanche de Montagne MN229129
Latxa Black Face KT879161.1
Latxa Black Face KT879166.1
Latxa Black Face KT879165.1
Sardi MN229213
Merinizzata Italiana KF228663.1
Latxa Blonde Face KT879040.1
Churra de Terra Quente DQ491707.1
Churra de Terra Quente DQ491706.1
Norduz KF677204.1
Latxa Black Face KT879174.1
Latxa Black Face KT879181.1
D'man MN229195
Beni-Guil MN229162
German Merino JN573937.1
Sopravissana KF228608.1
Gentile di Puglia KF228669.1
Daglic KF677078.1
German Merino JN574174.1
German Merino JN573948.1
Timahdite MN229251
Sopravissana KF228597.1
Merinizzata Italiana KF302459.1
Sopravissana KF302458.1
Sopravissana KF228592.1
Sopravissana KF302456.1
Gentile di Puglia KF302457.1
Blanche de Montagne MN229144
Blanche de Montagne MN229137
Blanche de Montagne MN229142
Blanche de Montagne MN229140
Blanche de Montagne MN229138
Timahdite MN229258
Saloia DQ491628.1
Churra Badana DQ491629.1
Blanche de Montagne MN229133
Latxa Blonde Face KT879046.1
Saloia DQ491715.1
Saloia DQ491630.1
Gentile di Puglia KF228691.1
Churra Badana DQ491647.1
Churra Algarvia DQ491646.1
Sarda KF228833.1
Gentile di Puglia KF228674.1
Saloia DQ491669.1
Comisana KF228720.1_B
Gentile di Puglia KF228686.1

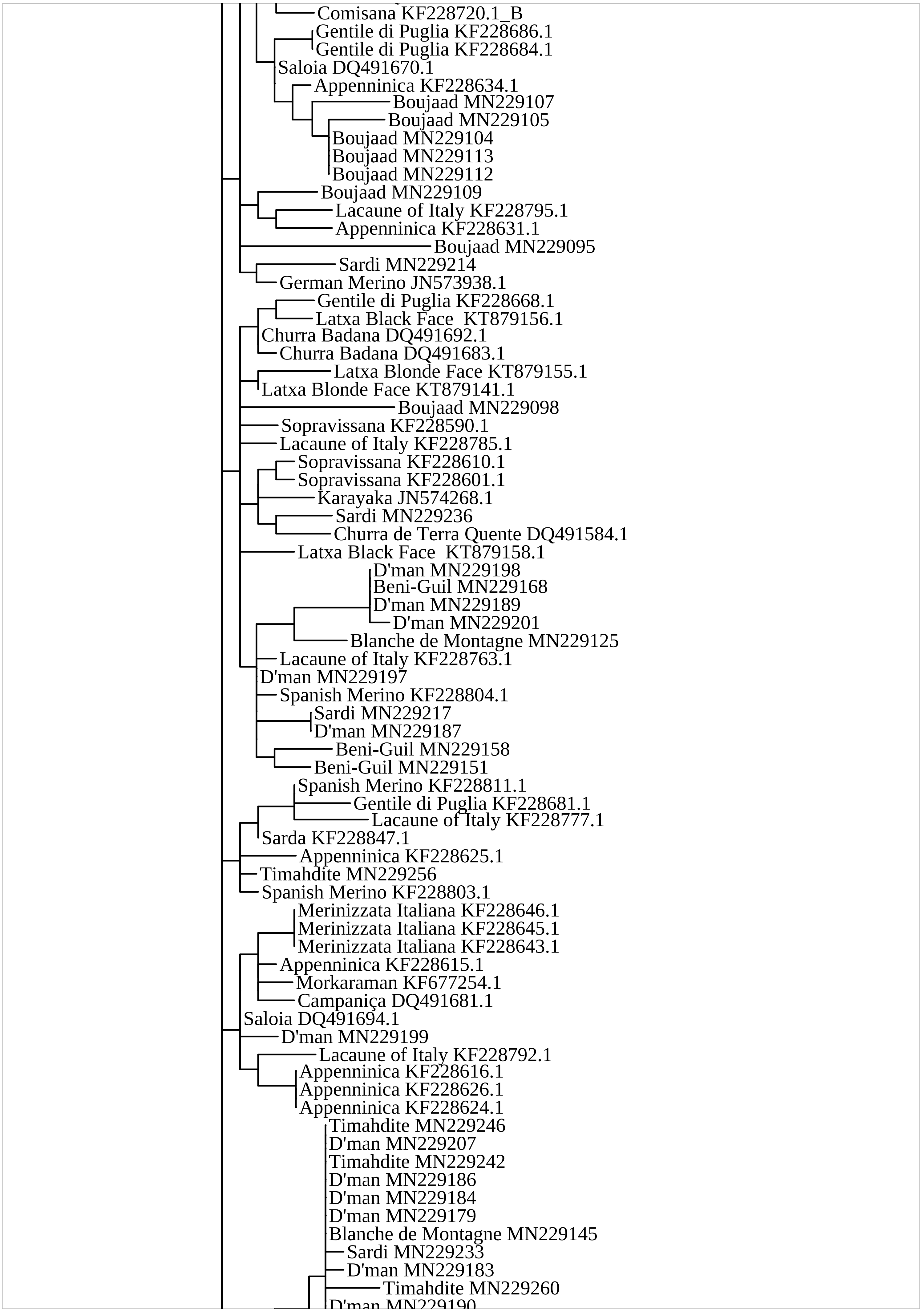
Comisana KF228720.1_B
Gentile di Puglia KF228686.1
Gentile di Puglia KF228684.1
Saloia DQ491670.1
Appenninica KF228634.1
Boujaad MN229107
Boujaad MN229105
Boujaad MN229104
Boujaad MN229113
Boujaad MN229112
Boujaad MN229109
Lacaune of Italy KF228795.1
Appenninica KF228631.1
Boujaad MN229095
Sardi MN229214
German Merino JN573938.1
Gentile di Puglia KF228668.1
Latxa Black Face KT879156.1
Churra Badana DQ491692.1
Churra Badana DQ491683.1
Latxa Blonde Face KT879155.1
Latxa Blonde Face KT879141.1
Boujaad MN229098
Sopravissana KF228590.1
Lacaune of Italy KF228785.1
Sopravissana KF228610.1
Sopravissana KF228601.1
Karayaka JN574268.1
Sardi MN229236
Churra de Terra Quente DQ491584.1
Latxa Black Face KT879158.1
D'man MN229198
Beni-Guil MN229168
D'man MN229189
D'man MN229201
Blanche de Montagne MN229125
Lacaune of Italy KF228763.1
D'man MN229197
Spanish Merino KF228804.1
Sardi MN229217
D'man MN229187
Beni-Guil MN229158
Beni-Guil MN229151
Spanish Merino KF228811.1
Gentile di Puglia KF228681.1
Lacaune of Italy KF228777.1
Sarda KF228847.1
Appenninica KF228625.1
Timahdite MN229256
Spanish Merino KF228803.1
Merinizzata Italiana KF228646.1
Merinizzata Italiana KF228645.1
Merinizzata Italiana KF228643.1
Appenninica KF228615.1
Morkaraman KF677254.1
Campaniça DQ491681.1
Saloia DQ491694.1
D'man MN229199
Lacaune of Italy KF228792.1
Appenninica KF228616.1
Appenninica KF228626.1
Appenninica KF228624.1
Timahdite MN229246
D'man MN229207
Timahdite MN229242
D'man MN229186
D'man MN229184
D'man MN229179
Blanche de Montagne MN229145
Sardi MN229233
D'man MN229183
Timahdite MN229260
D'man MN229190

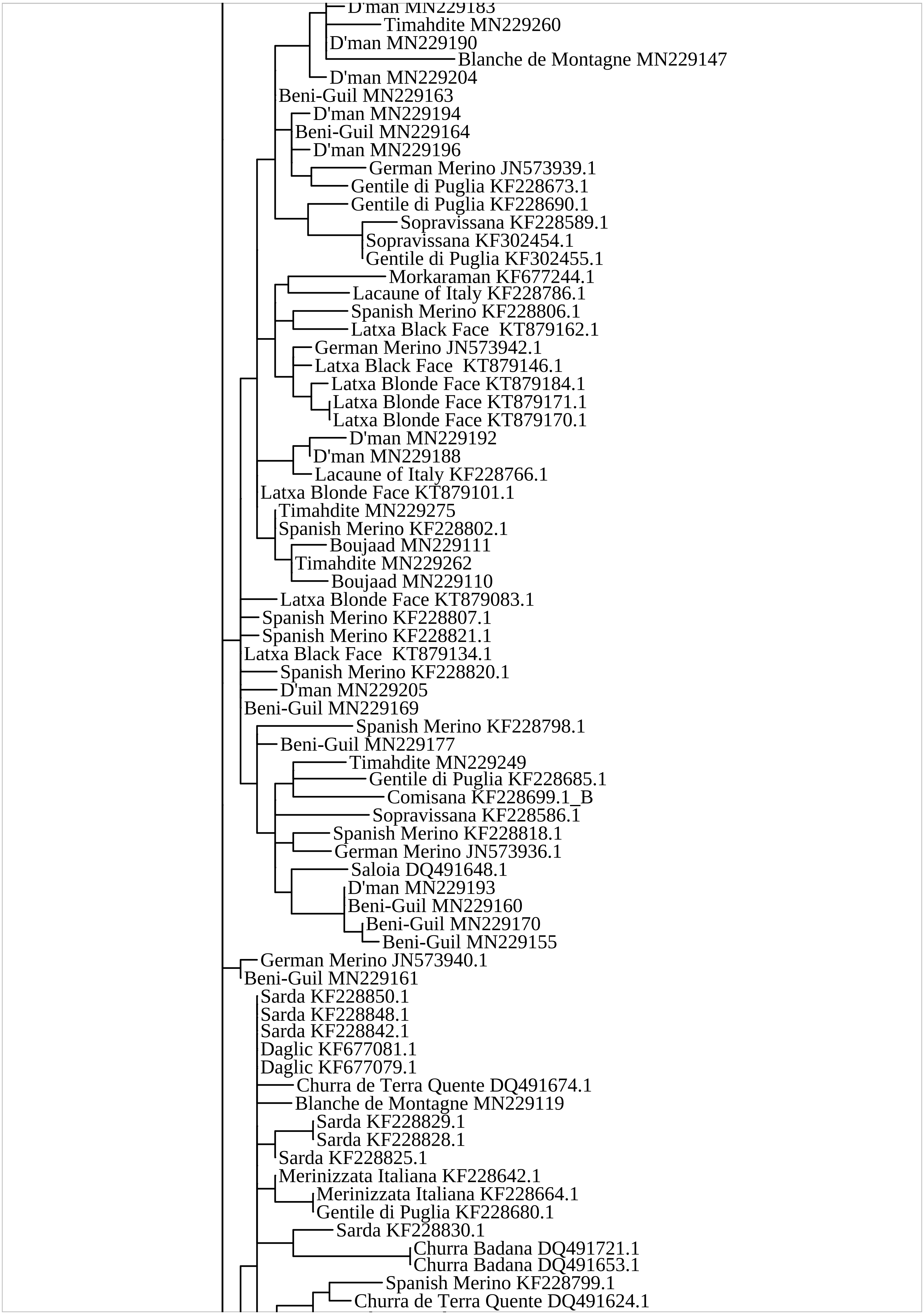
D'man MN229183
Timahdite MN229260
D'man MN229190
Blanche de Montagne MN229147
D'man MN229204
Beni-Guil MN229163
D'man MN229194
Beni-Guil MN229164
D'man MN229196
German Merino JN573939.1
Gentile di Puglia KF228673.1
Gentile di Puglia KF228690.1
Sopravissana KF228589.1
Sopravissana KF302454.1
Gentile di Puglia KF302455.1
Morkaraman KF677244.1
Lacaune of Italy KF228786.1
Spanish Merino KF228806.1
Latxa Black Face KT879162.1
German Merino JN573942.1
Latxa Black Face KT879146.1
Latxa Blonde Face KT879184.1
Latxa Blonde Face KT879171.1
Latxa Blonde Face KT879170.1
D'man MN229192
D'man MN229188
Lacaune of Italy KF228766.1
Latxa Blonde Face KT879101.1
Timahdite MN229275
Spanish Merino KF228802.1
Boujaad MN229111
Timahdite MN229262
Boujaad MN229110
Latxa Blonde Face KT879083.1
Spanish Merino KF228807.1
Spanish Merino KF228821.1
Latxa Black Face KT879134.1
Spanish Merino KF228820.1
D'man MN229205
Beni-Guil MN229169
Spanish Merino KF228798.1
Beni-Guil MN229177
Timahdite MN229249
Gentile di Puglia KF228685.1
Comisana KF228699.1_B
Sopravissana KF228586.1
Spanish Merino KF228818.1
German Merino JN573936.1
Saloia DQ491648.1
D'man MN229193
Beni-Guil MN229160
Beni-Guil MN229170
Beni-Guil MN229155
German Merino JN573940.1
Beni-Guil MN229161
Sarda KF228850.1
Sarda KF228848.1
Sarda KF228842.1
Daglic KF677081.1
Daglic KF677079.1
Churra de Terra Quente DQ491674.1
Blanche de Montagne MN229119
Sarda KF228829.1
Sarda KF228828.1
Sarda KF228825.1
Merinizzata Italiana KF228642.1
Merinizzata Italiana KF228664.1
Gentile di Puglia KF228680.1
Sarda KF228830.1
Churra Badana DQ491721.1
Churra Badana DQ491653.1
Spanish Merino KF228799.1
Churra de Terra Quente DQ491624.1

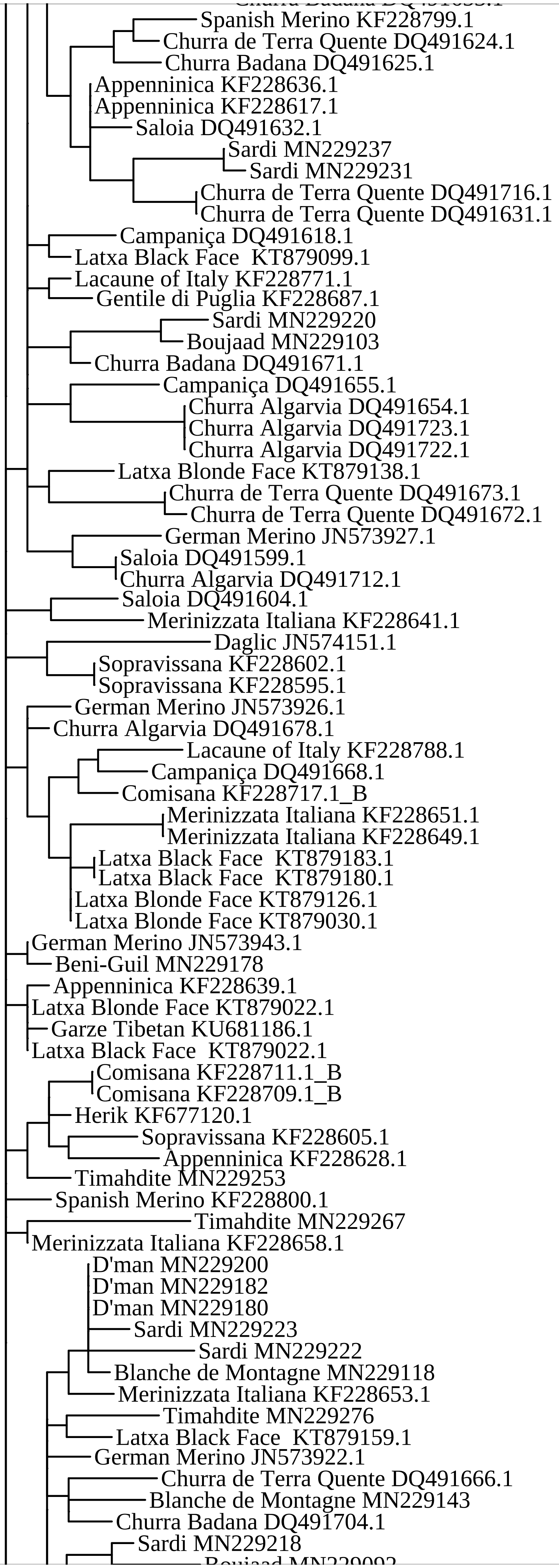
Spanish Merino KF228799.1
Churra de Terra Quente DQ491624.1
Churra Badana DQ491625.1
Appenninica KF228636.1
Appenninica KF228617.1
Saloia DQ491632.1
Sardi MN229237
Sardi MN229231
Churra de Terra Quente DQ491716.1
Churra de Terra Quente DQ491631.1
Campaniça DQ491618.1
Latxa Black Face KT879099.1
Lacaune of Italy KF228771.1
Gentile di Puglia KF228687.1
Sardi MN229220
Boujaad MN229103
Churra Badana DQ491671.1
Campaniça DQ491655.1
Churra Algarvia DQ491654.1
Churra Algarvia DQ491723.1
Churra Algarvia DQ491722.1
Latxa Blonde Face KT879138.1
Churra de Terra Quente DQ491673.1
Churra de Terra Quente DQ491672.1
German Merino JN573927.1
Saloia DQ491599.1
Churra Algarvia DQ491712.1
Saloia DQ491604.1
Merinizzata Italiana KF228641.1
Daglic JN574151.1
Sopravissana KF228602.1
Sopravissana KF228595.1
German Merino JN573926.1
Churra Algarvia DQ491678.1
Lacaune of Italy KF228788.1
Campaniça DQ491668.1
Comisana KF228717.1_B
Merinizzata Italiana KF228651.1
Merinizzata Italiana KF228649.1
Latxa Black Face KT879183.1
Latxa Black Face KT879180.1
Latxa Blonde Face KT879126.1
Latxa Blonde Face KT879030.1
German Merino JN573943.1
Beni-Guil MN229178
Appenninica KF228639.1
Latxa Blonde Face KT879022.1
Garze Tibetan KU681186.1
Latxa Black Face KT879022.1
Comisana KF228711.1_B
Comisana KF228709.1_B
Herik KF677120.1
Sopravissana KF228605.1
Appenninica KF228628.1
Timahdite MN229253
Spanish Merino KF228800.1
Timahdite MN229267
Merinizzata Italiana KF228658.1
D'man MN229200
D'man MN229182
D'man MN229180
Sardi MN229223
Sardi MN229222
Blanche de Montagne MN229118
Merinizzata Italiana KF228653.1
Timahdite MN229276
Latxa Black Face KT879159.1
German Merino JN573922.1
Churra de Terra Quente DQ491666.1
Blanche de Montagne MN229143
Churra Badana DQ491704.1
Sardi MN229218

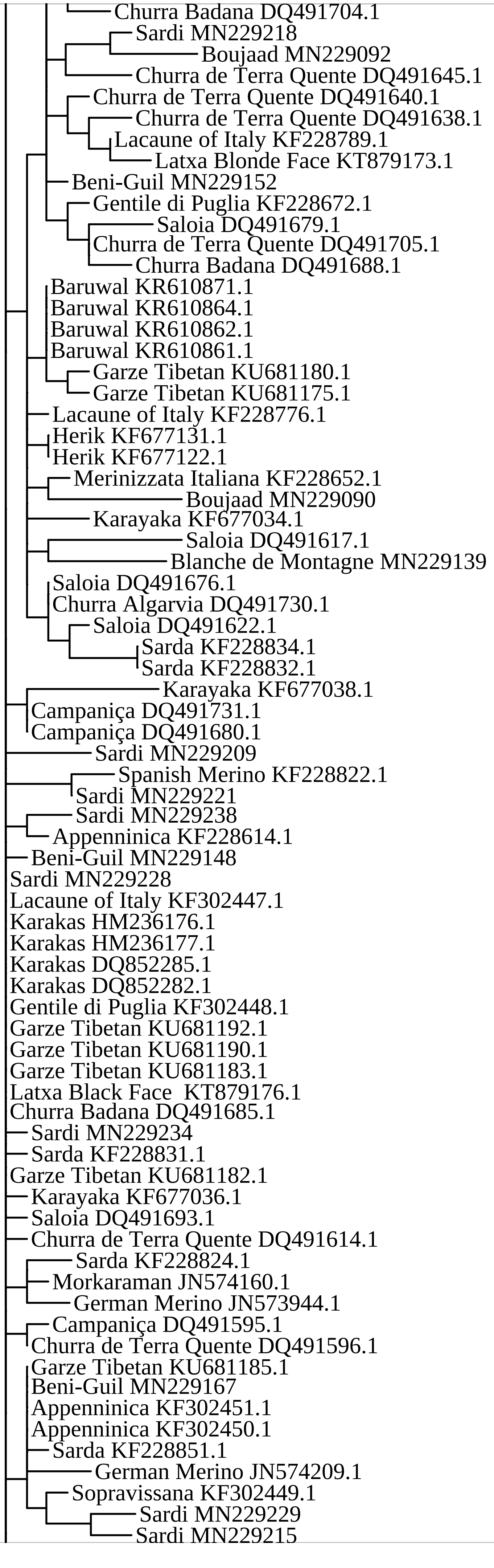
Churra Badana DQ491704.1
Sardi MN229218
Boujaad MN229092
Churra de Terra Quente DQ491645.1
Churra de Terra Quente DQ491640.1
Churra de Terra Quente DQ491638.1
Lacaune of Italy KF228789.1
Latxa Blonde Face KT879173.1
Beni-Guil MN229152
Gentile di Puglia KF228672.1
Saloia DQ491679.1
Churra de Terra Quente DQ491705.1
Churra Badana DQ491688.1
Baruwal KR610871.1
Baruwal KR610864.1
Baruwal KR610862.1
Baruwal KR610861.1
Garze Tibetan KU681180.1
Garze Tibetan KU681175.1
Lacaune of Italy KF228776.1
Herik KF677131.1
Herik KF677122.1
Merinizzata Italiana KF228652.1
Boujaad MN229090
Karayaka KF677034.1
Saloia DQ491617.1
Blanche de Montagne MN229139
Saloia DQ491676.1
Churra Algarvia DQ491730.1
Saloia DQ491622.1
Sarda KF228834.1
Sarda KF228832.1
Karayaka KF677038.1
Campaniça DQ491731.1
Campaniça DQ491680.1
Sardi MN229209
Spanish Merino KF228822.1
Sardi MN229221
Sardi MN229238
Appenninica KF228614.1
Beni-Guil MN229148
Sardi MN229228
Lacaune of Italy KF302447.1
Karakas HM236176.1
Karakas HM236177.1
Karakas DQ852285.1
Karakas DQ852282.1
Gentile di Puglia KF302448.1
Garze Tibetan KU681192.1
Garze Tibetan KU681190.1
Garze Tibetan KU681183.1
Latxa Black Face KT879176.1
Churra Badana DQ491685.1
Sardi MN229234
Sarda KF228831.1
Garze Tibetan KU681182.1
Karayaka KF677036.1
Saloia DQ491693.1
Churra de Terra Quente DQ491614.1
Sarda KF228824.1
Morkaraman JN574160.1
German Merino JN573944.1
Campaniça DQ491595.1
Churra de Terra Quente DQ491596.1
Garze Tibetan KU681185.1
Beni-Guil MN229167
Appenninica KF302451.1
Appenninica KF302450.1
Sarda KF228851.1
German Merino JN574209.1
Sopravissana KF302449.1
Sardi MN229229
Sardi MN229215

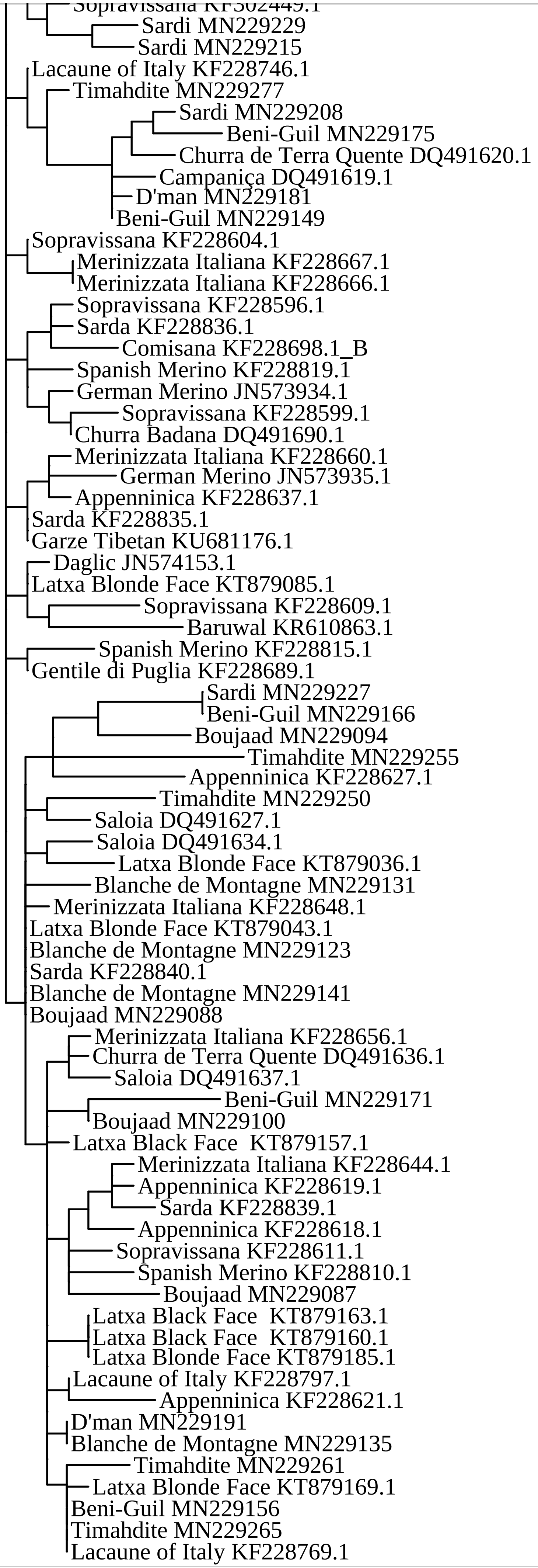
Sardi MN229229
Sardi MN229215
Lacaune of Italy KF228746.1
Timahdite MN229277
Sardi MN229208
Beni-Guil MN229175
Churra de Terra Quente DQ491620.1
Campaniça DQ491619.1
D'man MN229181
Beni-Guil MN229149
Sopravissana KF228604.1
Merinizzata Italiana KF228667.1
Merinizzata Italiana KF228666.1
Sopravissana KF228596.1
Sarda KF228836.1
Comisana KF228698.1_B
Spanish Merino KF228819.1
German Merino JN573934.1
Sopravissana KF228599.1
Churra Badana DQ491690.1
Merinizzata Italiana KF228660.1
German Merino JN573935.1
Appenninica KF228637.1
Sarda KF228835.1
Garze Tibetan KU681176.1
Daglic JN574153.1
Latxa Blonde Face KT879085.1
Sopravissana KF228609.1
Baruwal KR610863.1
Spanish Merino KF228815.1
Gentile di Puglia KF228689.1
Sardi MN229227
Beni-Guil MN229166
Boujaad MN229094
Timahdite MN229255
Appenninica KF228627.1
Timahdite MN229250
Saloia DQ491627.1
Saloia DQ491634.1
Latxa Blonde Face KT879036.1
Blanche de Montagne MN229131
Merinizzata Italiana KF228648.1
Latxa Blonde Face KT879043.1
Blanche de Montagne MN229123
Sarda KF228840.1
Blanche de Montagne MN229141
Boujaad MN229088
Merinizzata Italiana KF228656.1
Churra de Terra Quente DQ491636.1
Saloia DQ491637.1
Beni-Guil MN229171
Boujaad MN229100
Latxa Black Face KT879157.1
Merinizzata Italiana KF228644.1
Appenninica KF228619.1
Sarda KF228839.1
Appenninica KF228618.1
Sopravissana KF228611.1
Spanish Merino KF228810.1
Boujaad MN229087
Latxa Black Face KT879163.1
Latxa Black Face KT879160.1
Latxa Blonde Face KT879185.1
Lacaune of Italy KF228797.1
Appenninica KF228621.1
D'man MN229191
Blanche de Montagne MN229135
Timahdite MN229261
Latxa Blonde Face KT879169.1
Beni-Guil MN229156
Timahdite MN229265
Lacaune of Italy KF228769.1

Supplementary Data S3: Supplementary figures

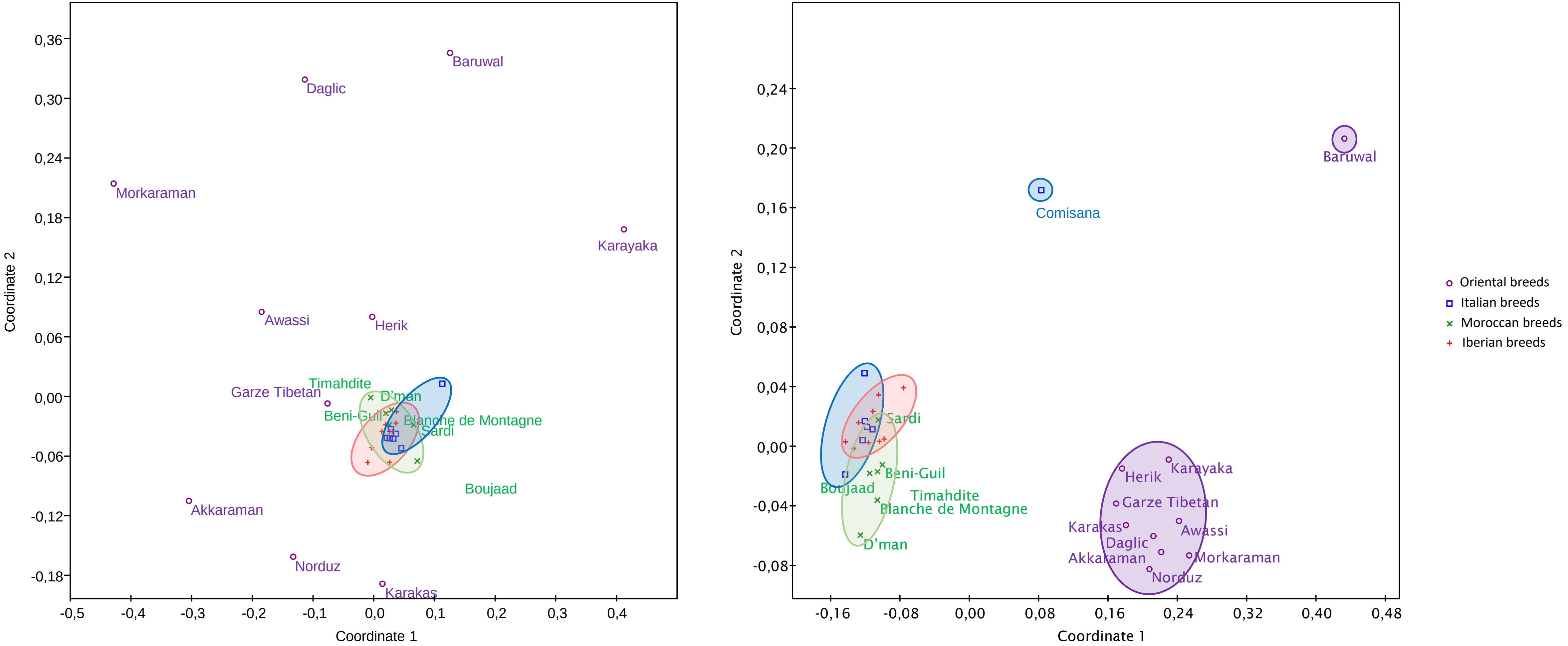

A: N-MDS of Dxy genetic distance

B: N-MDS of Da genetic distance

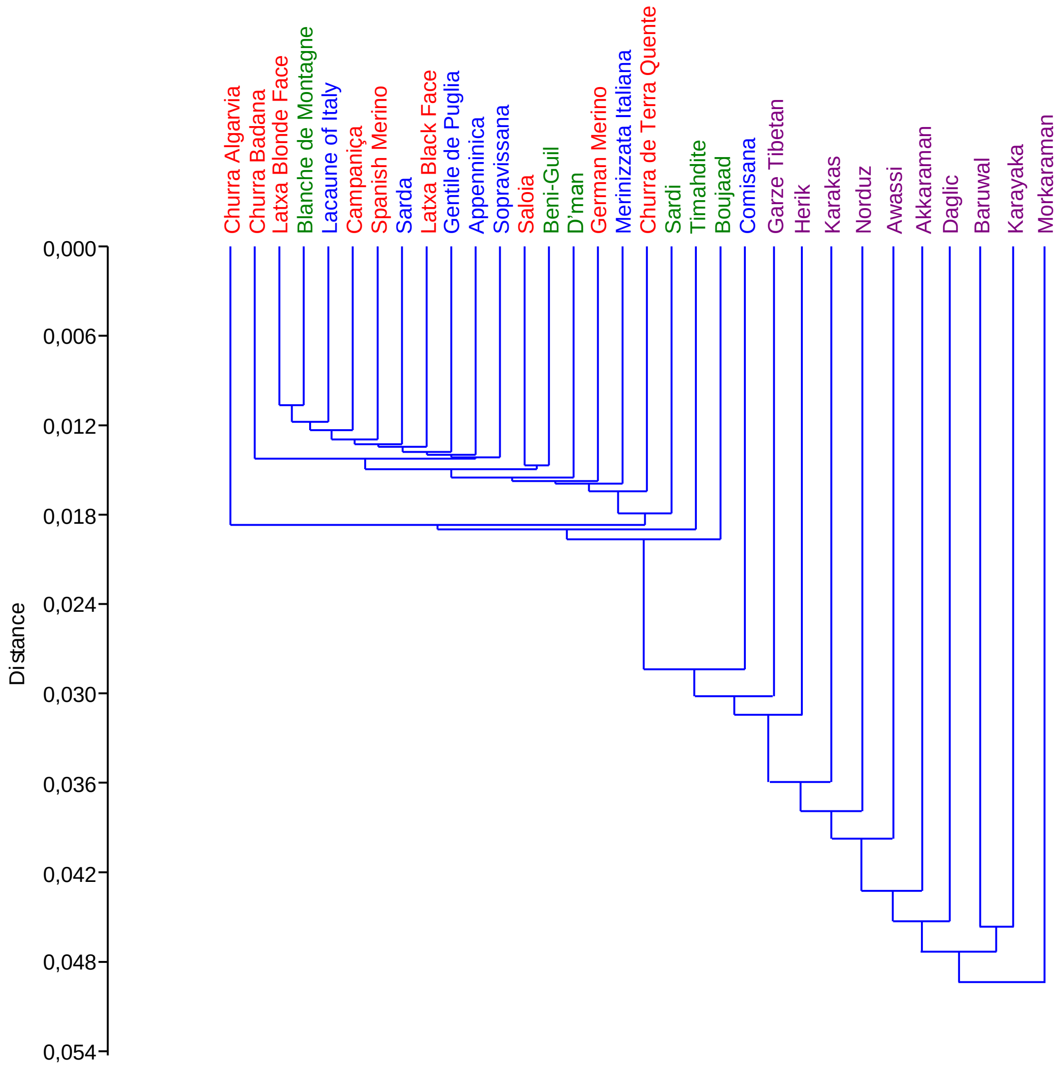

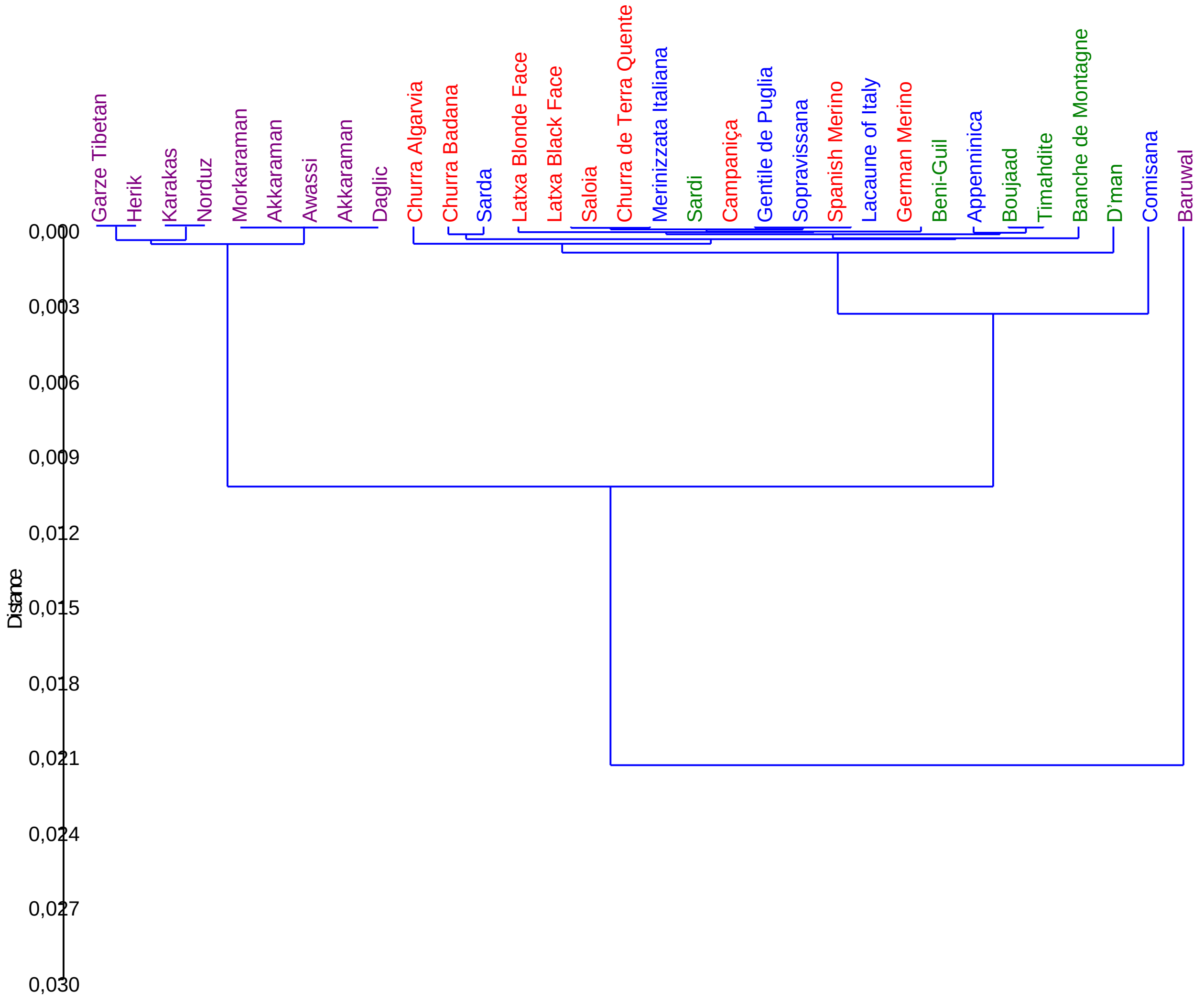

C: Cluster Analysis of Dxy genetic distance

D: Cluster Analysis of Da genetic distance

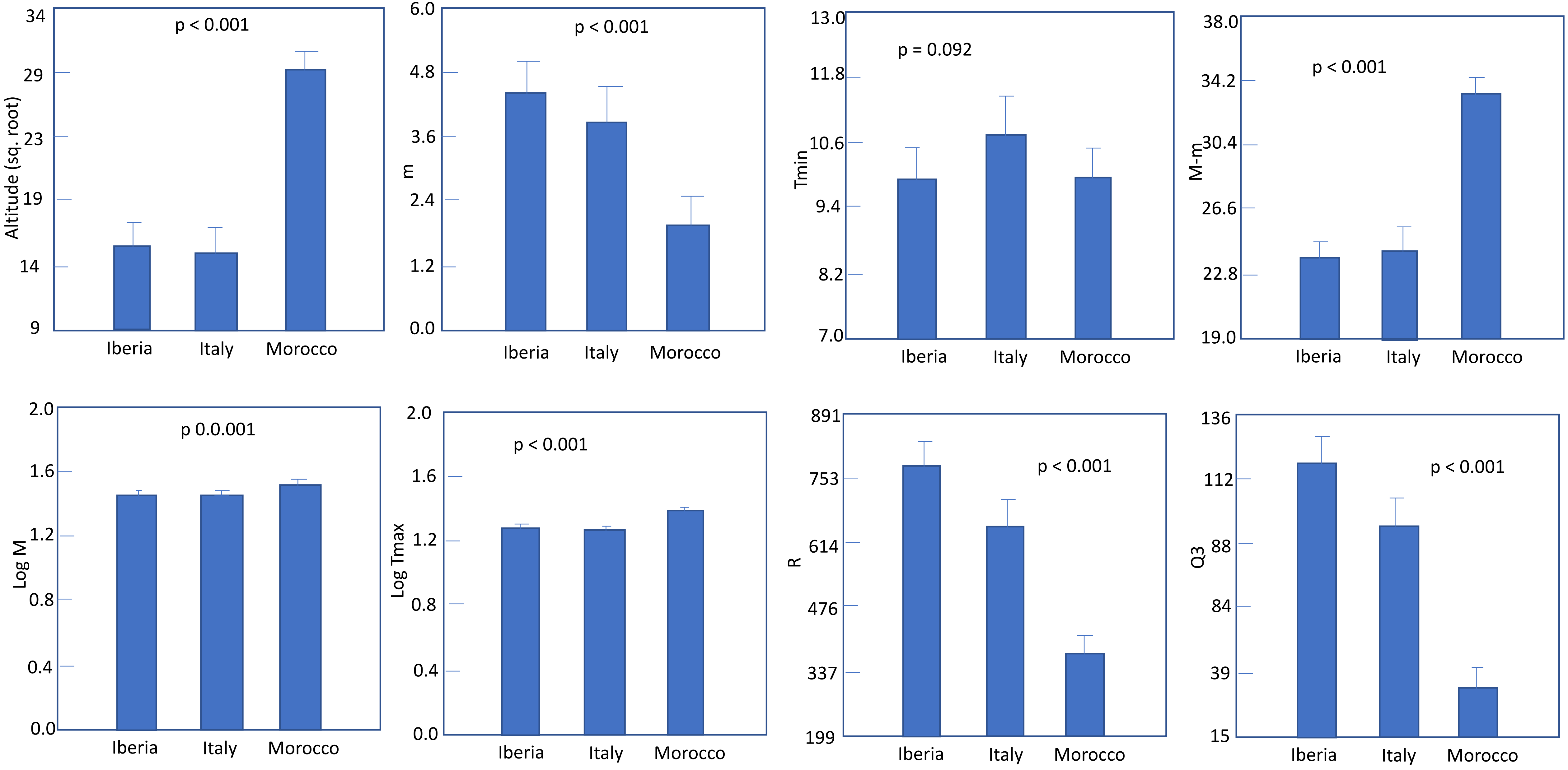

E: Variations in climatic parameters according to the three West Mediterranean regions. The p-value are given by ANOVA with 224 localities

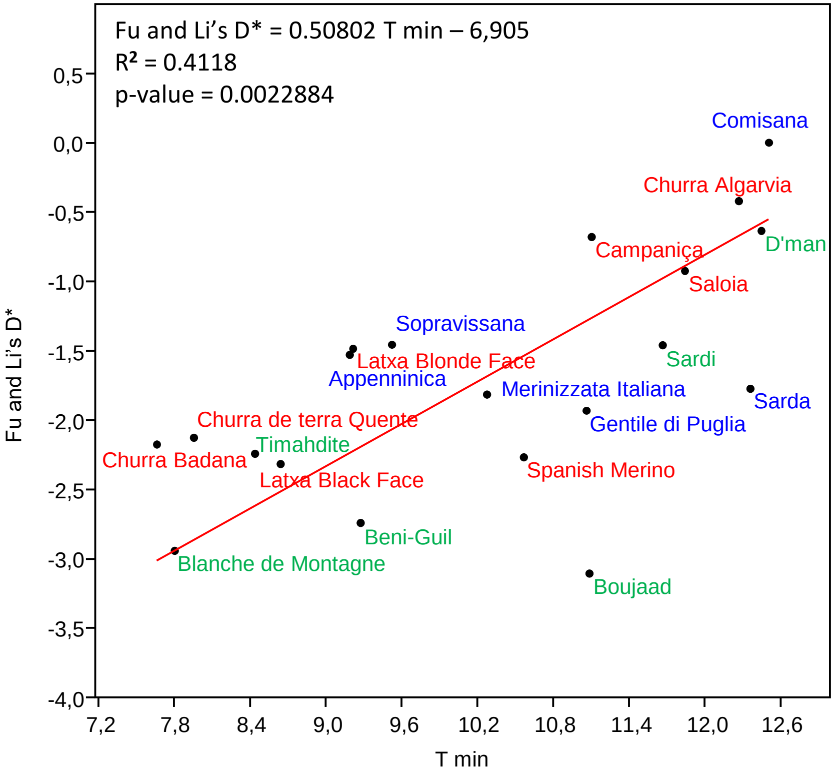

F: Relationship between T min and Fu and Li’s D*

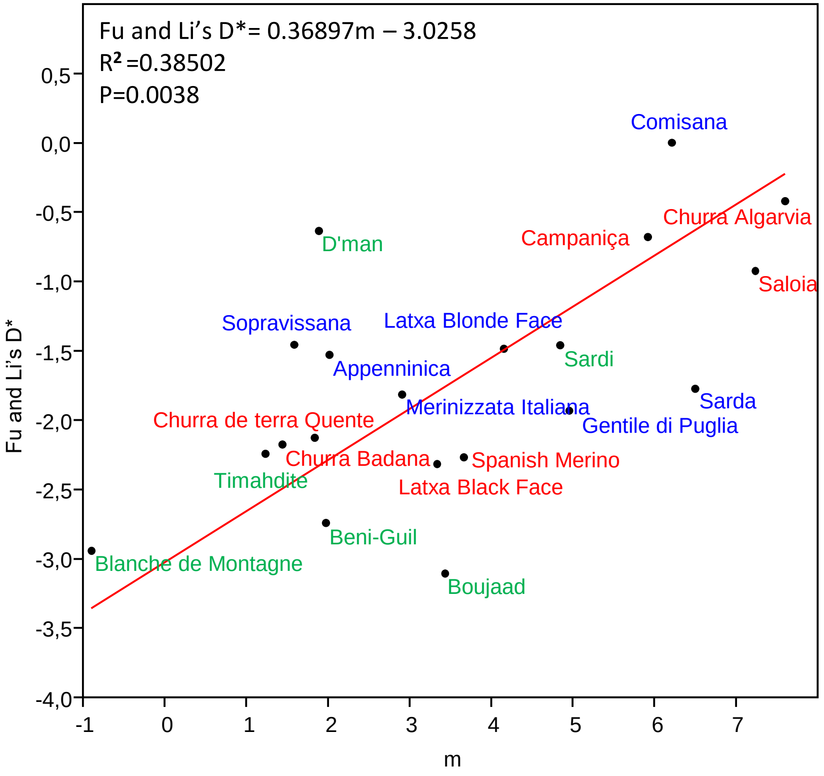

G: Relationship between m and Fu and Li’s D* (log transformed)

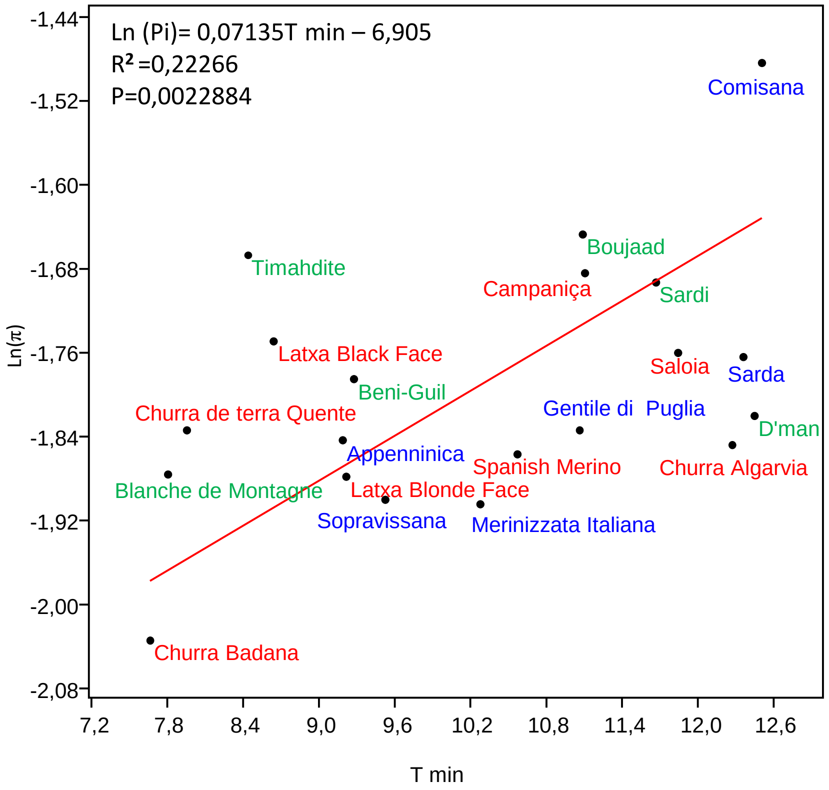

H: Relationship between Tmin and π (log transformed)

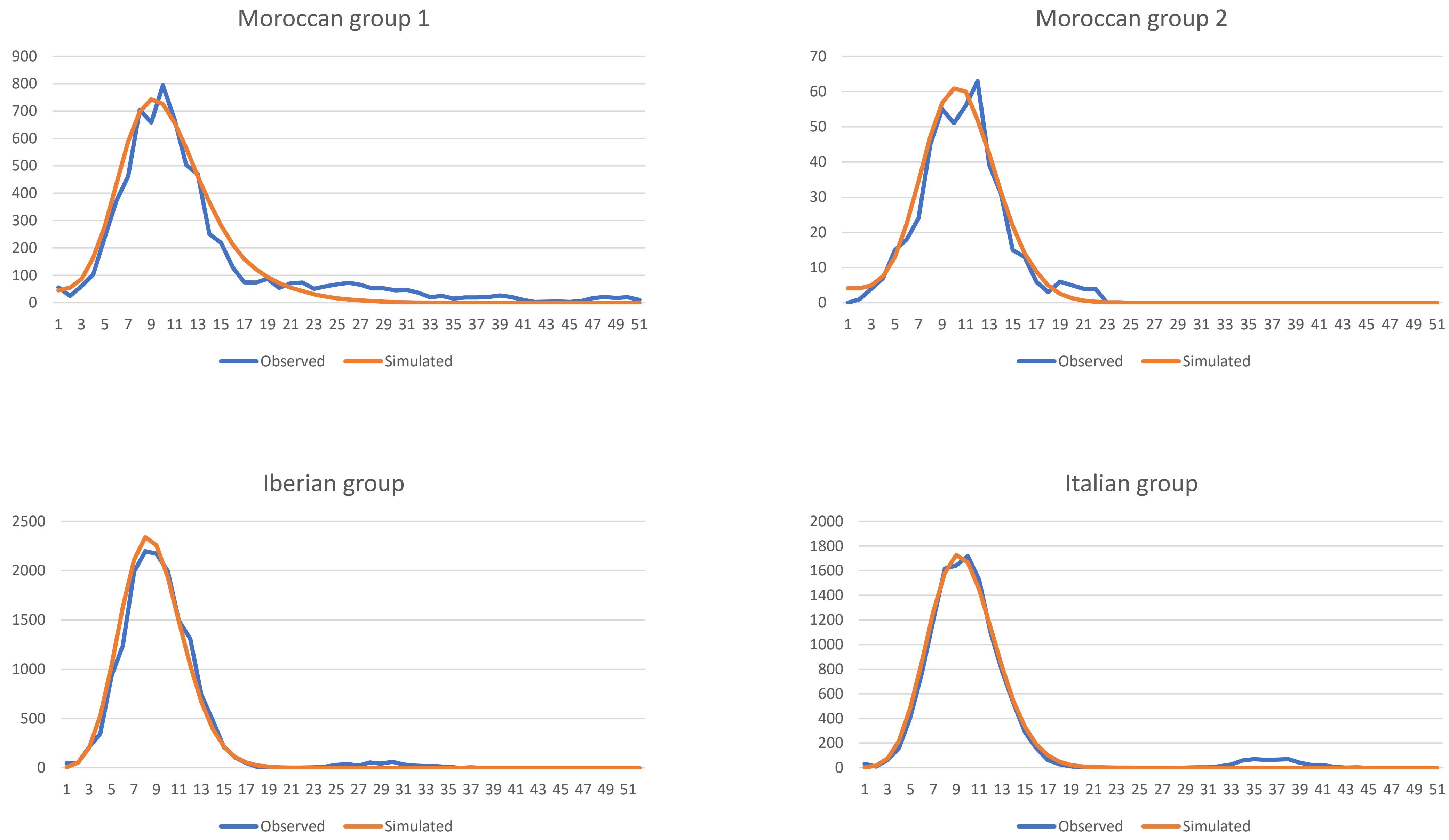

I: Mismatch Analysis for the groups of haplogroup B

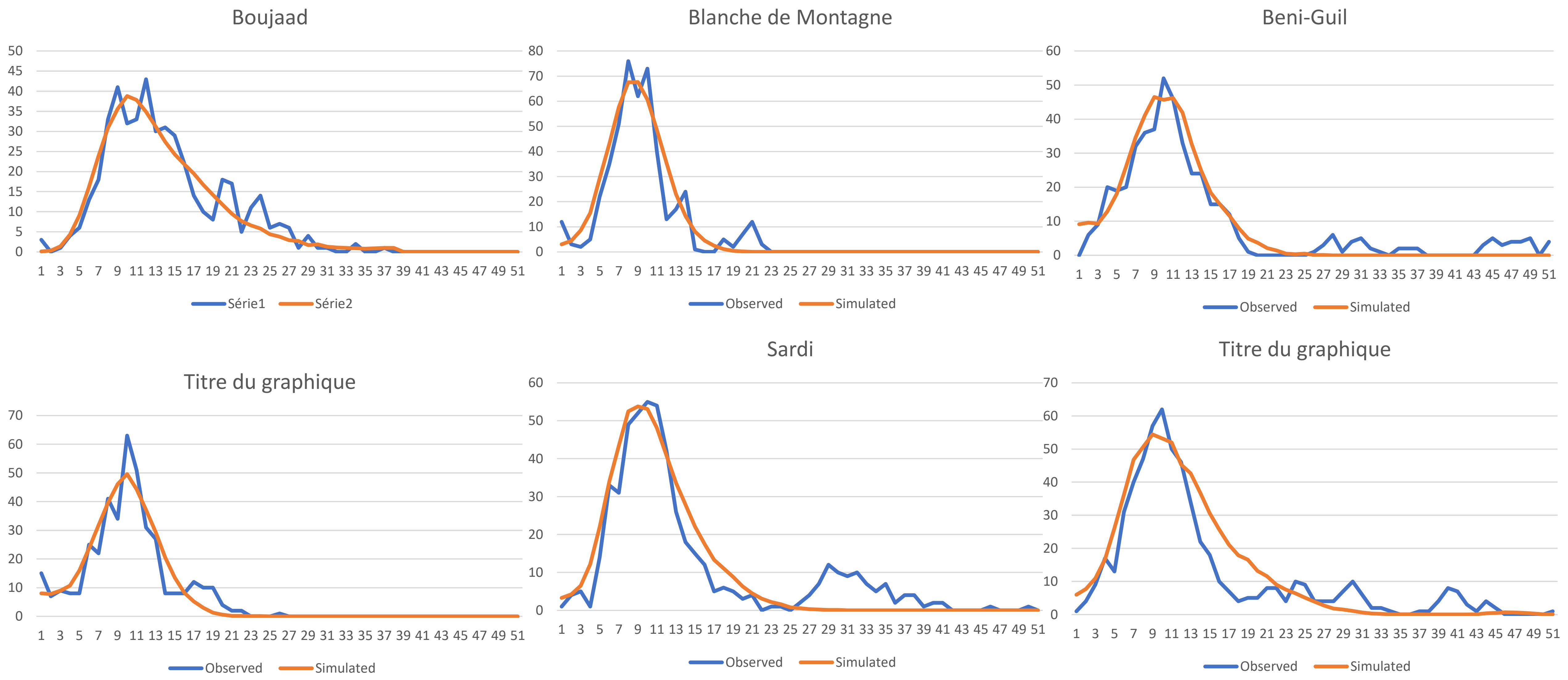

J: Mismatch Analysis for Moroccan breeds

Supplementary Data S4: Supplementary text

Suppl. S4. A. AMOVA results of the Moroccan, Iberian and Italian breed groups combinations comparisons.

	Among groups	Among populations within groups	Within populations
**Morocco/Iberia**	2.05***	0.67*	97.28***
**Morocco/Italy**	2.18***	1.64**	96.18***
**Italy/Iberia**	-0.26	3.26***	97***

Suppl. S4. B. Genetic diversity parameters and Fu's F statistics in analyzed Moroccan sheep breeds.

Breed	N[a]	H_n^b	$\mathrm{H_d^C}$ ± (SD)	Π[d] ± (SD)	S[e]	P[f]	$\mathrm{S_g^g}$	Fu Li F*	Fu Li D*	Fu's Fs	T[h]	Shannon	Haplogroup membership (%)				
													HA	HB	HC	HD	HE
**Blanche de Montagne**	32	24	0.970 ± (0.019)	0.013 ± (0.002)	44	16	28	-3.016*	-2.943*	-13.240***	-1.766	0.139	0	96.875	3.125	0	0
**Boujaad**	31	29	0.994 ± (0.011)	0.023 ± (0.002)	91	30	61	-3.280**	-3.105*	-18.224***	-2.111*	0.239	0	93.548	6.452	0	0
**Beni-Guil**	31	29	0.996 ± (0.009)	0.016 ± (0.003)	66	24	42	-2.091*	-2.741*	-22.713***	-1.953*	0.143	3.226	96.774	0	0	0
**D'man**	29	21	0.963 ± (0.023)	0.015 ± (0.001)	41	14	27	-0.880	-1.457	-7.424*	-1.744	0.000	0	100	0	0	0
**Sardi**	33	30	0.994 ± (0.009)	0.020 ± (0.003)	76	42	34	-1.821	-2.242	-18.582***	-1.692	0.229	6.060	93.940	0	0	0
**Timahdite**	37	32	0.988 ± (0.011)	0.022 ± (0.003)	56	27	29	-2.502	-0.679	-25.845***	-1.855*	0.281	0	91.892	8.108	0	0

* p <0.05; ** p <0.01. [a] Number of sequences. [b] Observed haplotypes (H_n). [c] Haplotype diversity (H_d). [d] Nucleotide diversity (Π). [e] Segregated sites (S). [f] Phylogenetically informative sites (P). [g] Singletons (S_g). [h] Tajima's D test.

The four breeds of North Morocco (Boujaad, Timahdite, Sardi and Beni-Guil) present the highest value of haplotypic and nucleotidic diversities (Hd, and π respectively). As regard haplogroup diversity measured by Shannon index, the D'man present the lowest value, followed by Blanche de Montagne and Beni-Guil. The neutrality test Fu's Fs indicates highly significant departure from neutrality for all the breeds, except for the D'man where its significant is moderate. Broadly speaking, Blanche de Montagne, Boujaad and Beni-Guil show significant value of Fu and Li F* and D*, and Tajima test

Suppl. S4.C. PCR amplification and sequencing of the mitochondrial DNA D-loop region.

The three primers used for both polymerase chain reaction (PCR) and sequencing of the D-loop region are indicated in Table 1. These primers were designed using the software Primer3 (at primer3.ut.ee), starting from the complete mtDNA sequence of reference *Ovis aries* in GenBank (NC_001941.1). The first coupleD1F/D1R allows the amplification of the first 655pb of mtDNA control region from 15391 to 16046, and the CR1F/D1R one allows the amplification of 634 pb from 15412 to 16046 (Suppl. S4.C. Table 1). Amplification reactions were carried out in a final volume of 20 µL PCR mix containing 70-80 ng of genomic DNA, 4 µl of buffer (High-Fidelity), 200 µM of each deoxynucleotide triphosphate (dATP, dCTP, dGTP and dTTP), 10 pmol of each primer and finally 0.2 µl of Taq polymerase (Phusion High-Fidelity from ThermoFisher scientific, Waltham, MA, USA). PCR thermal conditions were specified in Suppl. S4.C. Table 2. PCR products were checked by 1.5% agarose gel electrophoresis stained with ethidium bromide. Amplicons were treated with ExoSAP-IT in order to purified them from remaining dNTP and primers, as per manufactures instructions (ThermoFisher scientific, Waltham, MA, USA). Sequencing reactions were conducted using Big-Dye Terminator Cycle Sequencing kit (Applied Biosystems) according to the thermal conditions summarized in Suppl. S4.C. Table 3. The products were purified on Sephadex Columns (Sephadex™ LH-20, Sigma-Aldrich, Saint-Louis, MO, USA). Purified products were analyzed in ABI Prism 3100 Genetic Analyzer (Applied Biosystems).

Suppl. S4.C. Table 1: Primers used for the amplification and sequencing of the D-loop of Moroccan sheep.

Primer name	Primer sequence	Reference
**D1F**	**Forward** CCCCACTATCAACACCCAAAGC	Designed according to the NCBI sequence No.NC_001941.1
**CR1F**	**Forward** AGTTCTACTTAAACTATTCCCTG	
**D1R**	**Reverse** CAGGATACGCATGTTGACTAGA	

Suppl. S4.C. Table 2: PCR cycling conditions used for the amplification of the control region of sheep.

Step	Primer couple					
	D1F/D1R			CR1F/D1R		
	Temp[°C]	Time	N° of cycles	Temp[°C]	Time	N° of cycles
**Initial denaturation**	98	30 s	1	98	30 s	1
**Denaturation**	98	10 s		98	10 s	
**Annealing**	67.5	30 s	35	60.8	30 s	35
**Elongation**	72	20 s		72	20 s	
**Final elongation**	72	10 min	1	72	10 min	1

Suppl. S4.C. Table 3: Sequencing PCR conditions.

Step	Temp[°C]	Time	N° of cycle
**Initial denaturation**	94	4 min	1
**Denaturation**	96	10 s	
**Annealing**	58	5 s	25
**Elongation**	60	4 min	

Suppl. S4.D. Newick-Extra details.

To briefly explain our strategy, let's imagine an ancestral population composed by individuals belonging to two haplogroups A and B, including for example the individuals "a" and "b" belonging to the haplogroups A and B, respectively. It was assumed that this population split into subpopulations 1 and 2 containing the progeny a1 b1 and a2 b2, respectively. The individuals a1 and a2 share mutations acquired by their ancestor a, and ditto for b1 and b2. To quantify the proximity between subpopulations, we took into account the number of sister sequences retrieved in each pairwise combination, using the information contained in the topology of a phylogenetic tree.In order to entirely explore the phylogenetic tree, we constructed a program that (i) reads the topology expressed in the parenthetic tree (Newick) and (ii) records all the common points between the breeds in any combination, at the level of the terminal branches, and (iii) summarizes the data in an ExcelTM table. For example, in the simple parenthetic tree of figure 1 (A, B, C), (D, E), we retained the following information: A has one point in common with B and C, and ditto for B and C, and D and E. This program named Newick-Extra, written in R is available on request to the authors. Two levels of proximity have been considered. The first one corresponds to strict sister sequences (terminal branches), and the second one to two series of embedded sequences. The matrix gives for each breed pair the number of similarities that was later transformed into their proportions, giving an asymmetric matrix. This last one can be treated by distance measures applied to ordered variables.

Suppl. S4.D. Figure 1: Counting the two levels of connection numbers.

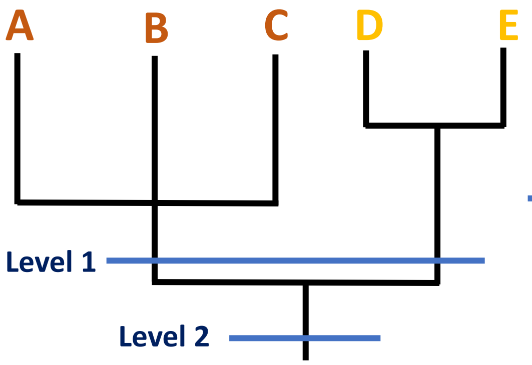

Matrix of Level 1

	A	B	C	D	E
**A**	0	1	1	0	0
**B**	1	0	1	0	0
**C**	1	1	0	0	0
**D**	0	0	0	0	1
**E**	0	0	0	1	0

Matrix of Level 2

	A	B	C	D	E
**A**	0	1	1	1	1
**B**	1	0	1	1	1
**C**	1	1	0	1	1
**D**	1	1	1	0	1
**E**	1	1	1	1	0

It should be noted that the treatment of the topology information at the level 2 produced a new table of affinities between breeds (data not shown) that generated a Cluster analysis in which the Moroccan breeds are scattered in three groups: among Italian and Iberian breeds. As a result, the level 2 gives a confusing view relatively to the Network analysis.
